# Methylomic analysis of monozygotic twins discordant for childhood psychotic symptoms

**DOI:** 10.1080/15592294.2015.1099797

**Published:** 2015-10-19

**Authors:** Helen L Fisher, Therese M Murphy, Louise Arseneault, Avshalom Caspi, Terrie E Moffitt, Joana Viana, Eilis Hannon, Ruth Pidsley, Joe Burrage, Emma L Dempster, Chloe C Y Wong, Carmine M Pariante, Jonathan Mill

**Affiliations:** 1 MRC Social, Genetic & Developmental Psychiatry Center; Institute of Psychiatry, Psychology & Neuroscience; King's College London; London, UK; 2 University of Exeter Medical School; University of Exeter; Exeter, Devon, UK; 3 Department of Psychology and Neuroscience; Duke University; Durham, NC, USA; 4 Department of Psychiatry and Behavioral Sciences; Duke University Medical School; Durham, NC, USA; 5 Garvan Institute of Medical Research; Darlinghurst, NSW, Australia; 6 Department of Psychological Medicine; Institute of Psychiatry, Psychology & Neuroscience; King's College London; London, UK

**Keywords:** biomarker, DNA methylation, epigenetics, longitudinal study, psychosis, schizophrenia, twins

## Abstract

Childhood psychotic symptoms are associated with increased rates of schizophrenia, other psychiatric disorders, and suicide attempts in adulthood; thus, elucidating early risk indicators is crucial to target prevention efforts. There is considerable discordance for psychotic symptoms between monozygotic twins, indicating that child-specific non-genetic factors must be involved. Epigenetic processes may constitute one of these factors and have not yet been investigated in relation to childhood psychotic symptoms. Therefore, this study explored whether differences in DNA methylation at age 10 were associated with monozygotic twin discordance for psychotic symptoms at age 12. The Environmental Risk (E-Risk) Longitudinal Twin Study cohort of 2,232 children (1,116 twin pairs) was assessed for age-12 psychotic symptoms and 24 monozygotic twin pairs discordant for symptoms were identified for methylomic comparison. Children provided buccal samples at ages 5 and 10. DNA was bisulfite modified and DNA methylation was quantified using the Infinium HumanMethylation450 array. Differentially methylated positions (DMPs) associated with psychotic symptoms were subsequently tested in post-mortem prefrontal cortex tissue from adult schizophrenia patients and age-matched controls. Site-specific DNA methylation differences were observed at age 10 between monozygotic twins discordant for age-12 psychotic symptoms. Similar DMPs were not found at age 5. The top-ranked psychosis-associated DMP (cg23933044), located in the promoter of the *C5ORF42* gene, was also hypomethylated in post-mortem prefrontal cortex brain tissue from schizophrenia patients compared to unaffected controls. These data tentatively suggest that epigenetic variation in peripheral tissue is associated with childhood psychotic symptoms and may indicate susceptibility to schizophrenia and other mental health problems.

## Introduction

Psychotic symptoms are reported by approximately 1 in 20 children at around 12 years of age^1^ and include paranoid thoughts, hearing or seeing things that others do not, and believing that others can read one's mind or vice versa. These symptoms often occur in children without a diagnosable illness but tend to be distressing^2^ and are highly predictive of schizophrenia, other psychiatric disorders, and suicide attempts in adulthood,^3^ as well as self-harm^1^ and suicide attempts^4^ in adolescence. Therefore, the etiology of these early psychotic symptoms requires further investigation. Understanding biological markers of childhood psychotic symptoms may facilitate the early detection of individuals at risk of developing severely disabling and life-threatening mental health problems and improve targeting of preventive interventions. Moreover, focusing on pre-clinical psychotic symptoms occurring early in life negates the effect of other potential confounders, including anti-psychotic medications, exposure to disorder-related traumatic events, such as forced hospitalization, and smoking, which are likely to influence epigenetic studies of adult schizophrenia patients.^5^

Quantitative genetic studies of childhood psychotic symptoms demonstrate higher concordance rates in monozygotic (MZ) (43%) than dizygotic (DZ) (22%) twins, highlighting a significant genetic influence on these symptoms.^1^ Despite this, there is still considerable discordance for psychotic symptoms within MZ twin-pairs,^1,6^ indicating that child-specific non-genetic factors are also important in mediating their onset. Epigenetic processes may constitute such a factor. Recent studies have started to investigate the role of epigenetic processes—acting to developmentally regulate gene expression via DNA, histone proteins, and chromatin modifications—in complex disease phenotypes. Epigenetic variation identified in post-mortem brain tissue has been associated with several neuropsychiatric conditions, including schizophrenia and other psychotic disorders in adults.^7-10^ Moreover, DNA methylation differences in peripheral tissue have been found in individuals with schizophrenia compared to unaffected controls.^11-13^

To date, epigenetic variation has not been explored in relation to childhood psychotic symptoms. Therefore, the aim of this study was to explore whether methylomic variation in early childhood (measured at ages 5 and 10) is associated with MZ twin discordance for psychotic symptoms at age 12. The use of symptom-discordant MZ twins represents a powerful strategy in epigenetic epidemiology because identical twins are matched for genotype, age, sex, paternal age, population cohort effects, and exposure to several shared environmental factors.^14^ DNA methylation differences are detectable between MZ twins in early childhood,^15^ and can change over a relatively short period of time,^15,16^ suggesting they may be markers of phenotypic variation. Indeed, DNA methylation differences in peripheral tissue samples have been associated with MZ twin discordance for several complex neuropsychiatric traits, including psychosis,^17^ autism,^18^ and depression.^19^ By utilizing prospectively collected epigenomic and phenotypic data we were able to examine the temporal relationship between epigenetic variation and onset of psychotic symptoms. Finally, given the tissue-specific nature of epigenetic processes, we aimed to explore whether differential methylation patterns associated with childhood psychotic symptoms in peripheral DNA samples reflect schizophrenia-associated variation in post-mortem brain tissue.

## Results

### DNA methylation differences at age 10 in MZ twins discordant for age-12 psychotic symptoms

**Figure 1.**
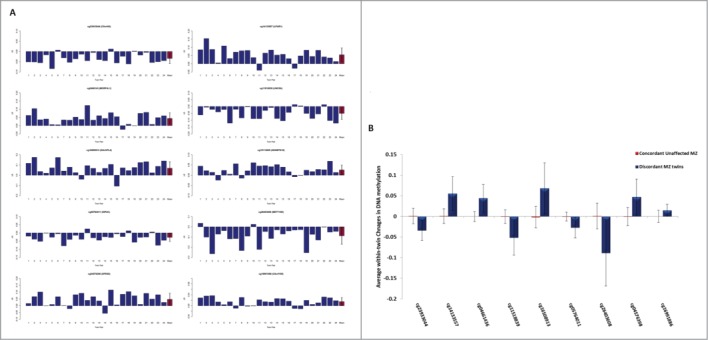
**(A)** Graphs showing the difference in DNA methylation (Δβ) at age 10 between each pair of monozygotic (MZ) twins discordant for psychotic symptoms at age 12 (affected twin – unaffected co-twin) for each of the 10 top-ranked probes. Mean within-twin pair Δβ across all 24 MZ twin-pairs is highlighted in red. Consistent within-twin pair differences in DNA methylation at age 10 are observed across discordant MZ twin pairs (n = 24) at the 10 top-ranked differentially methylated positions (DMPs). (**B)** Graph showing average within-twin β difference (Δβ) at age 10 of psychosis-discordant MZ twins and the age-matched concordant unaffected MZ twins (20 twin pairs). DNA methylation levels were available for 9/10 psychosis-associated DMPs. Average within-twin differences in DNA methylation are significantly larger (*P* < 0.005 for all comparisons) at 9 of the top-ranked DMPs in psychosis-discordant twins compared to twins concordant for no psychotic symptoms. Between groups comparison of mean within-twin β differences was examined using a 2-sample t-test. Error bars represent +/− the standard deviation of the mean within-twin pair Δβ.

**Figure 2.**
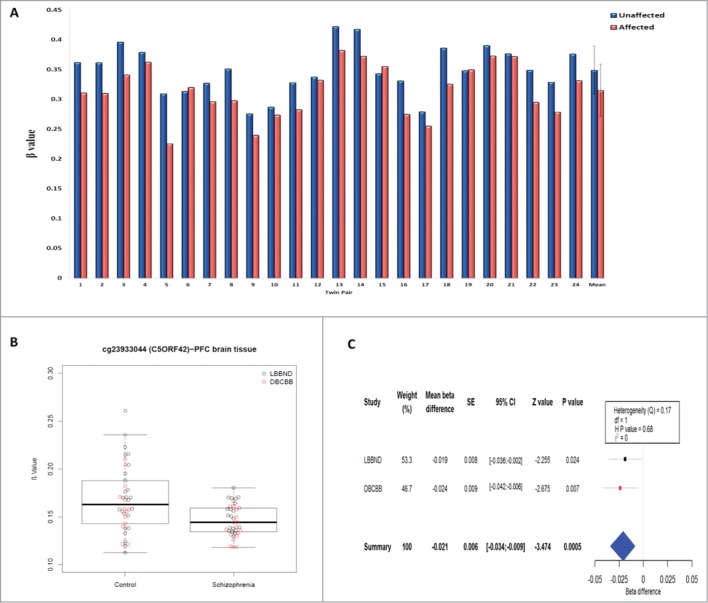
The top-ranked differentially methylated probe (cg23933044) is consistently hypomethylated at age 10 in monozygotic (MZ) twins with age-12 psychotic symptoms compared to their unaffected co-twin, and is also hypomethylated in adult prefrontal cortex (PFC) post-mortem tissue from schizophrenia patients compared to matched control subjects. (**A*****)*** CpG site, cg23933044, is consistently hypomethylated at age 10 across 24 MZ twins discordant for psychotic symptoms at age 12 (*P = 6.76×10*^*−7*^) **(B)** Comparison of DNA methylation at cg2393304 in 38 schizophrenia and 38 control post-mortem PFC samples confirms psychosis-associated hypomethylation (*P = 0.0005*). (**C**) Forest plot depicting a fixed-effects meta-analysis of a comparison of 2 independent schizophrenia brain cohorts: London Brain Bank for Neurodegenerative Disorders (LBBND) and Douglas Bell-Canada Brain Bank (DBCBB). Squares: mean β difference, Horizontal lines: 95% confidence intervals (CI): Blue diamond: overall mean β difference for meta-analysis, SE: Standard Error, df: degrees of freedom, H P value: Heterogeneity *P* value, Q: chi-squared statistic, I^2^: percentage of the variability in effect estimates that is due to heterogeneity rather than sampling error.

As expected, within-twin patterns of DNA methylation at age 10 were highly correlated across all MZ pairs (across all 391,565 probes included in the analysis, the average within-twin Pearson's *r* = 0.98) and no difference in overall mean DNA methylation (calculated by averaging across all analyzed probes) was observed between affected and unaffected twins (*P* = 0.61), indicating that childhood psychotic symptoms are not associated with any systemic changes in DNA methylation. In contrast, DNA methylation at individual CpG sites demonstrated considerable variability within discordant MZ twin pairs (**Fig. S1B**). **Table 1** shows the top ten DMPs at age 10, which were associated with psychotic symptoms at age 12 (nominal *P* < 5×10^−5^), with a more extensive list of DMPs (nominal *P* < 0.001) given in **Table S1**. The top-ranked DMPs were characterized by consistent psychosis-associated within-pair differences in DNA methylation across the twin pairs (**Fig. 1A**). The top-ranked DMP, cg23933044, located in the promoter regulatory region of *C5ORF42*, was characterized by reduced DNA methylation in affected twins compared with their unaffected co-twins (mean Δβ = −0.034, *P* = 6.76×10^−7^) (**Fig. 2A**). The analyses were re-run for the top-ranked DMPs (*P* < 5×10^−5^) adjusting for internalizing and externalizing problems at age 10 and depression symptoms at age 12, with minimal effect on the reported associations (see **Table S2**).

**Table 1.** The top-ranked DMPs at age 10 in monozygotic twin pairs discordant for psychotic symptoms at age 12

	Age 10					Age 5					
Probe ID	Affected Twin Mean	Co-Twin Mean	Mean Δβ	*P* value	Empirical *P* value^*^	Mean Δβ	*P*value	Hg19	Illumina Gene Annotation	Probe Type	Gene Annotation from GREAT(Distance from TSS)
cg23933044	0.315	0.349	−0.034	6.76E-07	<0.0001	0.008	0.518	Chr5:37249909	C5orf42	II	C5orf42 (−380)
cg14133557	0.796	0.742	0.055	1.65E-06	<0.0001	0.017	0.402	Chr9:113802005		II	LPAR1 (−1641)
cg04661436	0.795	0.751	0.044	1.81E-06	0.0001	0.023	0.057	Chr15:79169207	MORF4L1	II	MORF4L1 (+4036), CTSH (+68212)
cg11518839	0.378	0.428	−0.051	8.11E-06	<0.0001	−0.004	0.796	Chr5:176216711		II	UNC5A (−20848), TSPAN17 (+142324)
cg16508913	0.766	0.698	0.068	2.25E-05	0.0006	0.010	0.672	Chr11:11609419	GALNTL4	II	CSNK2A1 (−234516), GALNTL4 (+34141)
cg19115205	0.899	0.873	0.026	2.28E-05	0.0001	−0.003	0.741	Chr5:5062128	LOC340094	I	ADAMTS16 (−78314)
cg05764011	0.880	0.907	−0.027	2.55E-05	<0.0001	−0.003	0.824	Chr10:409541	DIP2C	II	ZMYND11 (+183608), DIP2C (+326066)
cg26403608	0.308	0.396	−0.088	2.58E-05	<0.0001	0.033	0.129	Chr17:2319719	METT10D	II	MNT (−15462), METTL16 (+95480)
cg04576398	0.747	0.700	0.047	3.10E-05	<0.0001	0.002	0.861	Chr12:132262872	SFRS8	II	MMP17 (−50068), SFSWAP (+67238)
cg16991886	0.903	0.888	0.015	3.58E-05	0.0003	−0.001	0.689	Chr6:11832079		I	HIVEP1 (−180644), C6orf105 (−52800)
cg08061902	0.848	0.818	0.0298	3.71E-05	0.0001	0.025	0.106	Chr19:40169418	LOC400696	II	LGALS14 (−25527), LGALS16 (+22861)
cg27418099	0.744	0.690	0.0541	3.92E-05	<0.0001	−0.009	0.757	Chr16:88941395	CBFA2T3	II	PABPN1L (−8328), CBFA2T3 (+102108)
cg20546928	0.068	0.057	0.0107	4.47E-05	<0.0001	0.001	0.767	Chr8:27167985	PTK2B;TRIM35	II	TRIM35 (+848)

*Note.* Ranked by age-10 *P* value. Δβ, difference in DNA methylation; DMPs, differentially methylated positions; GREAT, Genomic Regions Enrichment of Annotations Tool; Hg19, Human Genome build 19; TSS, transcription start site.

* An empirical *P* value was calculated by dividing the number of permutations that are at least as significant as the true result (*P*<0.00005) by the number of permutations performed (10,000).

We next tested the specificity of our psychosis-associated DMPs by comparing average within-twin DNA methylation differences at the top ten loci in 20 age-matched concordant unaffected MZ twin pairs where neither twin had psychotic symptoms at age 12. DNA methylation data was available for 9/10 top-ranked psychosis-associated DMPs. Average within-twin Δβ were significantly larger in the psychosis-discordant twins compared to twins concordant for no psychotic symptoms (*P* < 0.005 for all comparisons, see **Fig. 1B**).

Gene ontology (GO) enrichment analysis identified 128 nominally significant enriched terms (see **Table S3** for genes included in the analysis) for the DMPs (*P* < 0.001) identified in the age 10 analysis, including several related to the etiology of psychosis, such as glutamatergic synaptic transmission (GO:0051966, *P* = 0.0034) and neuron projection development (GO:0031175, *P* = 0.0064). However, no enrichment term remained significant after Bonferroni correction for multiple testing.

### Investigating top-ranked age-10 DMPs in schizophrenia post-mortem brain tissue

We examined DNA methylation at the top-ranked age-10 DMPs identified in our twin study (nominal *P* < 5×10^−5^) in prefrontal cortex (PFC) samples from two independent cohorts of adult schizophrenia patients and matched non-psychiatric control samples (total n = 38 schizophrenia and n = 38 control samples; see **Table S4**). The top-ranked DMP associated with childhood psychotic symptoms (cg2393304) was also significantly hypomethylated (Mean Δβ = −0.021, *P* = 0.0005, Bonferroni adjusted *P* value = 0.013) in a fixed-effects meta-analysis of the two independent PFC brain cohorts (**Fig. 2**). No significant differences in the PFC were observed for the other top-ranked age-10 discordant-twin DMPs.

### DNA methylation differences at age 5 in discordant MZ twins

We next assessed DNA methylation at age 5 in the majority of twin pairs profiled at age 10 (n = 18) in order to investigate whether site-specific DNA methylation differences between MZ twins discordant for age-12 psychotic symptoms were also present at this earlier age. **Table 2** shows within-pair DMPs at age 5 nominally associated with psychotic symptoms at age 12 (*P* < 5×10^−5^), with a more extensive list of DMPs (nominal *P* < 0.001) given in **Table S5**. None of the top-ranked age-10 DMPs (nominal *P* < 5×10^−5^) were characterized by significant within-pair differences at age 5 (**Table 1**). There was no correlation in effect sizes between ages 5 and 10 for these loci (r = 0.09, *P* = 0.76). Next, we examined within-pair differences at age 10 at the top-ranked DMPs identified at age 5. Top-ranking DMP (cg10377582), located in the *POU6F1* gene (Mean Δβ = −0.077, *P* = 3.03×10^−5^), identified in the age-5 analysis was also nominally significantly hypomethylated at age 10 (Mean Δβ = −0.04, *P* = 0.047). However, overall, there was no correlation in effect sizes between ages 5 and 10 for these loci (r = 0.2, *P* = 0.57). None of the top-ranked DMPs (*P* < 5×10^−5^) at age 5 were associated with schizophrenia in our PFC schizophrenia meta-analysis.

**Table 2.** The top ranked DMPs at age 5 in monozygotic twin pairs discordant for psychotic symptoms at age 12

	Age 5					Age 10					
Probe ID	Affected Twin Mean	Co-Twin Mean	Mean Δβ	*P* value	Empirical *P* value^*^	Mean Δβ	*P* value	Hg19	Illumina Gene Annotation	Probe Type	Gene Annotation from GREAT (Distance from TSS)
cg15031661	0.150	0.130	0.020	1.26E-05	0.0001	−0.001	0.805	Chr1:238323226	FMN2	I	FMN2 (+1419), GREM2 (+518858)
cg26432347	0.082	0.063	0.018	1.87E-05	0.0001	0.002	0.508	Chr6:30818615	FLOT1	I	FLOT1 (−184)
cg11356706	0.070	0.061	0.010	1.95E-05	<0.0001	0.006	0.113	Chr20:604240	SCRT2	I	SCRT2 (+582)
cg16011679	0.116	0.094	0.022	2.59E-05	0.0002	−0.008	0.190	Chr1:85497983	C1orf52	I	SYDE2 (−58668), BCL10 (+17191)
cg21480740	0.899	0.883	0.016	2.64E-05	0.0002	0.015	0.079	Chr7:158511954		II	ESYT2 (−196875), VIPR2 (+118455)
cg10377582	0.443	0.520	−0.077	3.03E-05	<0.0001	−0.040	0.047	Chr12:49899061	POU6F1	I	POU6F1 (−20845), DAZAP2(−19713)
cg24085426	0.863	0.839	0.024	3.05E-05	0.0001	0.006	0.506	Chr12:45869545		II	AMIGO2 (−109545), FAM113B(−26726)
cg03044239	0.072	0.080	−0.008	3.15E-05	<0.0001	−0.003	0.529	Chr3:139549743	MRAS	II	MRAS (−454)
cg06547771	0.044	0.052	−0.008	3.34E-05	0.0001	−0.002	0.366	Chr11:43336969	TTC17	I	TTC17 (−41)
cg14659771	0.138	0.114	0.024	3.59E-05	<0.0001	0.005	0.391	Chr2:231625606		I	PSMD1 (−4215)
cg24391460	0.525	0.462	0.062	4.12E-05	<0.0001	−0.005	0.793	Chr7:78922102	MAGI2	I	MAGI2 (−1277)

*Note.* Ranked by age-5 *P* value. Δβ, difference in DNA methylation; DMPs, differentially methylated positions; MZ, monozygotic; GREAT, Genomic Regions Enrichment of Annotations Tool; TSS, transcription start site.

* An empirical *P* value was calculated by dividing the number of permutations that are at least as significant as the true result (*P* < 0.00005) by the number of permutations performed (10,000).

### Changes in DNA methylation between ages 5 and 10 in discordant MZ twins

Finally, we examined CpG sites characterized by psychosis-associated intra-individual changes in DNA methylation between ages 5 and 10. For each probe, we calculated the change in DNA methylation from age 5 to age 10 (longitudinal Δβ) for each individual and examined the difference in longitudinal Δβ between affected twins and their unaffected co-twin. **Table 3** lists the top-ranked Δβ between ages 5 and 10 associated with age-12 psychotic symptoms (*P* < 5×10^−5^), with a more extensive list of DMPs (nominal *P* < 0.001) in **Table S6**. The top-ranked probe (cg15797527), located in the Abelson helper integration-1 (*AHI1*) gene, was characterized by increased DNA methylation from ages 5 to 10 in affected twins compared to their unaffected co-twin (longitudinal Δβ* = 0.084; P* = 4.30×10^−6^). However, DNA methylation at this position was not associated with schizophrenia in our PFC samples (*P* = 0.678). GO term enrichment analysis found 67 nominally significant enriched categories for the DMPs (*P* < 0.001) identified in the longitudinal analysis (see **Table S7** for full gene list), with the top enriched GO categories relating to the presynaptic membrane (GO:0042734, *P* = 0.0032) and the perikaryon (GO:0043204, *P* = 0.0035). However, no enrichment term remained significant after Bonferroni correction for multiple testing.

**Table 3.** The top ranked CpG sites that show changes in DNA methylation levels between ages 5 and age 10 in the monozygotic twins discordant for psychotic symptoms at age 12

Probe ID	Affected Twin Mean	Co-Twin Mean	Mean change in longitudinal Δβ	*P* value	Hg19	Illumina Gene Annotation	Probe Type	Gene Annotation from GREAT (Distance from TSS)
cg15797527	0.044	−0.040	0.084	4.30E-06	Chr6:135814781	AHI1	II	AHI1 (+4121), MYB (+312329)
cg10052038	0.040	−0.036	0.076	7.91E-06	Chr3:196293939	WDR53	II	FBXO45 (−1785)
cg27403609	−0.016	0.028	−0.044	1.14E-05	Chr2:11101403		I	PQLC3 (−194136), KCNF1 (+49341)
cg11556416	−0.034	0.041	−0.074	1.35E-05	Chr2:191879252	STAT1	I	STAT1 (−277)
cg13567282	0.023	−0.043	0.066	1.40E-05	Chr6:149653214	MAP3K7IP2	II	TAB2 (+13779), ZC3H12D (+152933)
cg19599395	0.066	−0.007	0.073	1.40E-05	Chr19:3837031	ZFR2	II	MATK (−50617), ZFR2 (+31995)
cg26998693	−0.024	0.013	−0.036	1.54E-05	Chr6:132834590	STX7	II	STX7 (−254)
cg19050351	0.037	−0.052	0.089	1.76E-05	Chr2:113820090	IL1F5	II	IL1F10 (−5456), IL36RN (+3406)
cg18260625	−0.011	0.015	−0.027	1.87E-05	Chr5:42951192		I	SEPP1 (−139169), C5orf39 (+89254)
cg14297966	−0.003	−0.057	0.055	2.40E-05	Chr9:35101708	STOML2	II	PIGO (−5163), STOML2 (+1445)
cg02524205	−0.038	0.054	−0.091	2.51E-05	Chr6:167559851		I	GPR31 (+11467), CCR6 (+34557)
cg12730562	−0.025	0.041	−0.067	2.87E-05	Chr11:44927876	TSPAN18	II	TP53I11 (+44731), TSPAN18 (+141901)
cg22827324	−0.017	0.022	−0.039	3.31E-05	Chr2:33612876	LTBP1	II	RASGRP3 (−126065), LTBP1 (+440508)
cg26613742	0.015	−0.053	0.068	3.42E-05	Chr19:14225000	PRKACA	II	SAMD1 (−23769), PRKACA (+3558)
cg03359468	0.014	−0.008	0.023	3.62E-05	Chr8:26240463	BNIP3L	I	BNIP3L (−59)
cg25367206	0.038	−0.020	0.059	3.82E-05	Chr8:142239055	SLC45A4	I	SLC45A4 (−383)
cg03916630	0.053	−0.040	0.092	4.01E-05	Chr10:45065415		II	TMEM72 (−341348), CXCL12 (−184871)

*Note.* Ranked by *P* value (*P* < 5×10^−05^). Δβ, difference in DNA methylation; DMPs, differentially methylated positions; GREAT, Genomic Regions Enrichment of Annotations Tool; Hg19, Human Genome build 19; TSS, transcription start site.

## Discussion

This is the first study to assess epigenetic variation in MZ twins discordant for childhood psychotic symptoms. We first examined genome-wide patterns of DNA methylation in buccal cell samples collected at age 10. The top-ranked DMP (cg23933044), which is hypomethylated in twins reporting psychotic symptoms at age 12 compared to their unaffected co-twin, is located in the promoter regulatory region of the protein coding gene *C5ORF42* overlapping a number of transcription factor binding sites (TFBSs). Interestingly, the same CpG site is significantly hypomethylated in post-mortem PFC samples from schizophrenia patients compared with matched control subjects. *C5ORF42* encodes a highly conserved 3,198 amino acid protein whose function is largely uncharacterized, although it is known to interact with genes important for neural development, including the p21-activating kinase 1 (*PAK1*) and the small ubiquitin-like modifier 1 (*SUMO1*).^20^ BioGPS, an online gene annotation resource, indicates that *C5ORF42* is widely expressed in a variety of tissues, including the brain and peripheral tissues.^21^ Of note, mutations in *C5ORF42* cause Joubert syndrome, a severe neurodevelopmental disorder,^20,22^ which can result in developmental delays similar to those seen in some individuals who have childhood psychotic symptoms or are later diagnosed with schizophrenia.^23^

Several of the other top-ranked DMPs identified at age 10 are located in the vicinity of genes that have previously been implicated in neurodevelopment and psychiatric disorders. For example, cg08263941, which is hypermethylated in affected twins compared to their unaffected co-twin, is located upstream of CHRM2, a gene implicated in memory and cognition and whose function is impaired in many neuropsychiatric disorders.^24-26^ Similarly, cg14133557, which is hypermethylated in affected twins compared to their unaffected co-twin, is located in the promoter of *LPAR1*, a gene important for cellular signaling; a *Lpar1* null mouse model has been shown to exhibit a schizophrenia-like pathology.^27^ Furthermore, DMPs cg05318275 and cg12252412 are associated with genes encoding two of the α_2_ adrenergic receptors (*ADRA1A* and *ADRA2A*, respectively*)*. These receptors have a role in regulating neurotransmitter release from sympathetic nerves and from adrenergic neurons in the central nervous system and have previously been implicated in psychiatric disorders.^28,29^ Finally, GO enrichment analysis highlighted significant enrichment for pathways associated with neurological processes, such as establishment of cell polarity and neuron projection development.

None of the top-ranked DMPs identified at age 10 show significant within-pair differences at age 5. We therefore examined psychosis-associated intra-individual changes in DNA methylation between ages 5 and 10. The top-ranked longitudinal DMP (cg15797527), which was significantly hypermethylated from age 5 to age 10 in the affected twins compared to their unaffected co-twins, is located in the *AHI1* gene. *AHI1* is required for both cerebellar and cortical development in humans and has previously been associated with schizophrenia,^30-32^ autism,^33^ and Joubert syndrome-related disorders.^34^ Genes annotated to the most DMPs between ages 5 and 10 in twins who went on to display psychotic symptoms highlighted significant GO term enrichment for neuronal cellular components including the bulbous end of neurons (perikaryon) and the presynaptic membrane, suggesting that epigenetic variation at these loci may be associated with neuronal dysfunction. Interestingly, previous imaging studies have reported dysregulated presynaptic membrane expression of key neurotransmitters in first-episode psychosis.^35^

### Methodological considerations

Despite the power of the discordant MZ twin approach for epigenetic epidemiology, there are several limitations to this study. First, only seven psychotic symptoms were assessed. However, the questions used are well-established and have been validated and used previously in studies of psychotic phenomena in children.^2,36-39^ Second, we could not ascertain the precise timing of the symptoms, as they were not inquired about until age 12. We therefore cannot conclude that the differences identified at age 10 predate the onset of psychotic symptoms, nor can we exclude the possibility of reverse causality. However, age 11-12 is thought to be the youngest age when children can understand the psychosis interview and provide valid responses.^1^ Third, our analysis utilized a small cohort of 48 twin children (24 MZ pairs) and is relatively underpowered to detect small changes in DNA methylation. Although no probe reached Bonferroni-corrected levels of significance, DNA methylation studies in other psychiatric phenotypes (and complex disorders in general) report similarly small absolute differences, and given the known non-independence of DNA methylation across the probes represented on the array,^40^ it is likely that conventional methods of global statistical significance are not optimal for these analyses. We also therefore calculated empirical *P* values for each of the top-ranked probes using a permutation testing approach as an alternative approach to assess significance.^41^ Fourth, as epigenetic studies cannot be performed in the brains of live children, the genome-wide data generated in this study are from peripheral buccal cell DNA rather than the brain. However, buccal cells were selected as the most suitable peripheral tissue because they derive from the same embryonic cells as brain tissue (ectoderm) and have less cellular heterogeneity compared with blood.^42^ Although there are well-documented tissue-specific differences in DNA methylation,^43^ we and others have shown that disease-associated changes in DNA methylation can be identified in peripheral tissues.^17-19,44-47^ Although these might not be directly involved in disease pathogenesis, they are of potential use as biomarkers of disease. Furthermore, in this study we confirmed disease-associated hypomethylation of our top-ranked DMP (cg23933044) using post-mortem PFC samples from schizophrenia patients, indicating that locus-specific methylation patterns identified in peripheral tissue may sometimes reflect disease-associated variation in the brain. Other DMPs identified in the buccal samples from our twins, however, were not confirmed in post-mortem brain tissue. Additionally, given that our sample were only aged 12 we cannot rule out the possibility that the unaffected co-twin might develop psychotic symptoms at a later age.

Technical validation, using bisulfite pyrosequencing, of our top-ranked DMP (cg23933044) was not performed in this study. We and others have previously successfully technically validated DNA methylation levels obtained from the 450K array using pyrosequencing, thus highlighting the reliability of this array.^18,19,48,49^ Moreover, the analytical sensitivity of bisulfite pyrosequencing is ~5–10% for individual CpG dinucleotides^50^ and, thus, does not have the sensitivity required to detect small (< 5%) changes in DNA methylation between cases and controls. Hence, we sought to replicate our DNA methylation findings using two independent brain cohorts instead of technically verifying our results. We believe this approach is more applicable to the current study given the small changes in DNA methylation (Δβ = 0.034) observed between discordant MZ twins. However, we only used brains from patients diagnosed with schizophrenia and, although childhood psychotic symptoms have been shown to be highly predictive of the later development of schizophrenia,^3,36^ they have also been shown to be associated with other mental health problems in adulthood.^3^ Thus, exploration of how our findings replicate in the brains of patients with other psychiatric disorders would be useful. One final caveat in this study is that chorionicity data were not available on these twins; a potential limitation, given that whether or not MZ twins share a placenta may influence epigenomic and transcriptional differences mediated by subtle differences in the prenatal environment.^51^

Despite these limitations, this paper provides a useful template for future epigenetic studies of psychiatric and behavioral phenotypes. First, employing a discordant twin design allowed us to control for genotype, age, sex, paternal age, population cohort effects, and exposure to several shared environmental factors (e.g., passive smoking within the home). Second, we utilized longitudinally collected data, which enabled us to compare within-individual differences over time and ascertain the temporal ordering of methylation differences and emergence of the phenotype. Finally, we attempted to perform cross-tissue replication of our peripheral tissue findings in post-mortem brain tissue samples. Incorporating the experimental design and analyses utilized in this study would strengthen future research efforts examining the role of epigenetic processes in the onset of mental health difficulties.

### Conclusion

In summary, we present the first evidence that site-specific epigenetic variation may be associated with childhood psychotic symptoms in peripheral DNA samples from symptom-discordant MZ twin pairs. The top-ranked psychosis-associated DMP (cg23933044) identified in this study was also significantly hypomethylated in the prefrontal cortex of adult schizophrenia patients compared to age-matched control subjects, suggesting that some peripheral biomarkers of disease may reflect later disease-associated variation in the brain.

## Materials and Methods

### Study cohort

Participants were recruited from the Environmental Risk (E-Risk) Longitudinal Twin Study, which tracks the development of a birth cohort of 1,116 British twin pairs (n = 2,232 individuals). The E-Risk sample was drawn from a larger representative birth register of same-sex twins born in England and Wales in 1994-1995.^52^ Full details about the sample are reported elsewhere.^53^ The sample includes 55% MZ and 45% dizygotic twin pairs. Sex is evenly distributed within zygosity (49% male). The children were originally seen when they were aged 5 and follow-up home visits took place when they were aged 7 (98% participation), 10 (96% participation), and 12 (96% participation) years. The Joint South London and Maudsley and the Institute of Psychiatry Research Ethics Committee approved each phase of the study (NRES 1997/122). Parents gave informed consent at every phase and children gave assent at the age-12 assessment phase.

### Measures

#### DNA samples

Buccal cell samples were obtained from children during home visits at ages 5 and 10. Genomic DNA was isolated from buccal cells using a standard procedure.^54^ DNA was tested for degradation and purity using spectrophotometry and gel electrophoresis prior to methylomic profiling.

#### Childhood psychotic symptoms

When the children were 12 years-old, psychotic symptoms were assessed in a private individual structured interview conducted by mental health trainees or professionals.^1,55^ Interviewers had no prior knowledge about the child. Seven psychotic symptoms were investigated related to delusions and hallucinations using items from the Dunedin Study's age-11 interview protocol,^36^ and the Avon Longitudinal Study of Parents and Children interview.^37,38^ Our protocol took a conservative approach to designating a child's report as a symptom. First, when a child endorsed any symptom, the interviewer probed using standard prompts designed to discriminate between experiences that were plausibly real (e.g., “I was followed by a man after school”) and potential symptoms (e.g., “I was followed by an angel who guards my spirit”) and wrote down the child's narrative description of the experience. Interviewers coded these descriptions 0, 1, or 2, indicating, respectively, “not a symptom,” “probable symptom,” and “definite symptom.” Second, a psychiatrist expert in schizophrenia, a psychologist expert in interviewing children, and a child and adolescent psychiatrist reviewed all the written narratives from the interviews to confirm the interviewers' codes. Third, experiences limited to the twin relationship (e.g., “My twin and I often know what each other are thinking”) were coded as “not a symptom.” A dichotomous variable was created representing children who reported no definite psychotic symptoms (n = 2,002, 94.1%) and those who reported at least one definite psychotic symptom (n = 125, 5.9%). Of these, 25 MZ twin pairs were discordant for the presence of a definite psychotic symptom at age 12, and of these pairs, 16 (64%) were male, all were Caucasian and UK-born. None of the twins in this sample took any medications for emotional, behavioral, or psychiatric conditions during childhood.

#### Potential confounders

Internalizing and externalizing problems at age 10 were assessed using the Child Behavior Checklist in face-to-face interviews with mothers,^56^ and the Teacher's Report Form by mail for teachers.^57^ The internalizing problems scale is the sum of items in the withdrawn and anxious/depressed subscales, and the externalizing problems scale is the sum of items from the aggressive and delinquent behavior subscales. We summed and standardized mothers and teachers reports to create cross-informant scales. At age 12, children completed the Children's Depression Inventory (CDI),^58^ with scores of 20 or more indicating clinically significant depressive symptoms.^59^

### DNA methylomic profiling

Genomic DNA (500 ng) was treated with sodium bisulfite using the EZ-96 DNA Methylation Kit (Zymo Research, CA, USA) following the manufacturers' protocol. DNA methylation was quantified using the Infinium HumanMethylation450 BeadChip array (Illumina, Inc., San Diego, California), as previously described.^60^ Each MZ twin pair was processed together throughout the entire experimental procedure to minimize potential within-pair batch effects, with all samples processed blind to phenotype. Genome Studio software (Illumina, Inc.) was used to extract signal intensities for each probe and perform initial quality control (QC). Further QC checks, quantile normalization, and separate background adjustment of methylated and unmethylated intensities of type I and II probes were undertaken using the dasen function in the R wateRmelon package (available from www.bioconductor.org).^60^ Samples with >5% of sites with a detection *P* value < 0.05 or a bead count < 3 in >5% of samples were removed from further analysis, together with their co-twin. Non-specific probes and probes on the X and Y chromosomes were removed.^61,62^ The final analyses included 391,565 probes, and incorporated data from 18 MZ pairs (36 individual samples) at age 5 and 24 MZ pairs (48 individual samples) at age 10. Polymorphic single nucleotide polymorphism (SNP) control probes (n = 65) located on the array were used to confirm monozygosity for all twin-pairs included in the final analysis.

### Statistical analyses

We assessed genome-wide patterns of DNA methylation at ages 5 and 10 in buccal cells from twins who went on to report psychotic symptoms at age 12, and their unaffected co-twins. Our primary focus was on within-pair DNA methylation differences detected at age 10, as this was the most proximal sampling collection point to when psychotic symptoms were assessed at age 12. All statistical analyses were performed using R (version 3.1.1). Our primary analyses employed paired t-tests to identify differentially methylated positions (DMPs) between affected (1 or more definite psychotic symptoms at age 12) and unaffected (no definite psychotic symptoms at age 12) twin children using DNA collected at age 10. The β value (β) is a ratio between methylated probe intensity and total probe intensities (sum of methylated and unmethylated probe intensities) and ranges from 0 to 1. Probes were ranked according to *P* value and Q-Q plots assessed to check for *P* value inflation (see **Fig. S1A**). For the top-ranked DMPs (nominal *P* < 5×10^−5^), linear regression was performed to adjust β values for internalizing and externalizing problems at age 10, to check that these difficulties were not accounting for any observed DNA methylation differences, together with clinically significant depressive symptoms at age 12, to ensure that these symptoms were not confounding the phenotypic difference between the twins. Paired t-tests were used to examine differences in these adjusted β values between affected and unaffected MZ twins at age 10 at the top-ranked DMPs (nominal *P* < 5×10^−5^). Analyses were repeated for buccal samples collected at age 5. Intra-individual changes in DNA methylation from ages 5 to 10 were calculated (longitudinal Δβ) with differences in longitudinal Δβ between affected twins and their unaffected co-twin assessed using paired t-tests. An empirical *P* value was calculated for top-ranked DMPs (P < 0.00005) in psychosis-discordant twins at age 10 (n = 24 twin pairs) and age 5 (n = 18 twin pairs) by randomly assigning twin status (affected/unaffected) for 10,000 permutations. An empirical *P* value was calculated by dividing the number of permutations, which were at least as significant as the true result (P < 0.00005) by the number of permutations performed (10,000).

The specificity of the 10 top-ranked psychosis-associated DMPs was determined by examining within-twin DNA methylation differences at these loci in 20 age-matched concordant unaffected control MZ twin pairs, with no history of age-12 psychotic symptoms. The average within-twin Δβ, at these loci, was calculated using permutation testing (n = 1,000). Briefly, concordant unaffected twin-pairs were randomly assigned to a dichotomous variable [0 (Twin 1) or 1 (Twin 2)] and the within-twin Δβ (β value of Twin 1 – β value of Twin 2) was calculated for each twin pair at each probe. Next, the average within-twin Δβ was calculated across all twin pairs at each probe, this was repeated 1,000 times. The average within-twin Δβ was calculated by taking the average of the permutated within-twin Δβ values obtained at each DMP of interest. The average within-twin Δβ for the unaffected concordant twins was then compared to the average within-twin Δβ calculated from the discordant MZ twins at each probe of interest using a two-sample t-test.

### Gene ontology term enrichment analysis

Gene ontology (GO) term enrichment analysis was performed on genes annotated (Illumina UCSC gene annotation) to the top-ranked DMPs (nominal *P* < 0.001) using the R package GOseqv1.18.1 (downloaded from Bioconductor).^63^ GOseq can be used to correct for the number of Illumina 450K probes in each gene during GO term enrichment analysis. The number of probes per gene was calculated in our final dataset to create a probability weighting function, which was then used in the GO term enrichment analysis.

### Post-mortem brain DNA methylation data

Prefrontal cortex (PFC) post-mortem brain samples were obtained from 20 schizophrenia cases' and 23 non-psychiatric controls' adult brains archived in the London Brain Bank for Neurodegenerative Disorders (LBBND) and from 18 schizophrenia cases' and 15 non-psychiatric controls' brains obtained from the Douglas Bell-Canada Brain Bank (DBCBB), Montreal (www.douglasbrainbank.ca) as described in Pidsley et al.^10^ Subjects were approached in life for written consent for brain banking, and all tissue donations were collected and stored following legal and ethical guidelines (NHS 08/MRE09/38; HTA license:12293; University of Exeter Medical School Research Ethics Committee:13/02/009). Briefly, genomic DNA was isolated using a standard phenol-chloroform extraction protocol. Schizophrenia patients were diagnosed by trained psychiatrists according to DSM criteria. Demographic information for the samples is summarized in **Table S4**. Samples were randomized with respect to gender and disease status to avoid batch effects throughout all experimental procedures. All microarray pre-processing and data normalization was performed as described above. A fixed effects meta-analysis of the two independent schizophrenia brain cohorts was performed using the metacont function from the meta package in R (http://cran.r-project.org/).^64^
