# Supplementary material for: Methylomic analysis of monozygotic twins discordant for childhood psychotic symptoms
Source: Epigenetics. 2015 Oct 19;10(11):1014–23. doi: 10.1080/15592294.2015.1099797 (PMC4867769; doi:10.1080/15592294.2015.1099797)
Supplement: 1099797_Supplemental_Material.doc [file kepi-10-11-1099797-s001.doc]

**Supplementary Material**

**
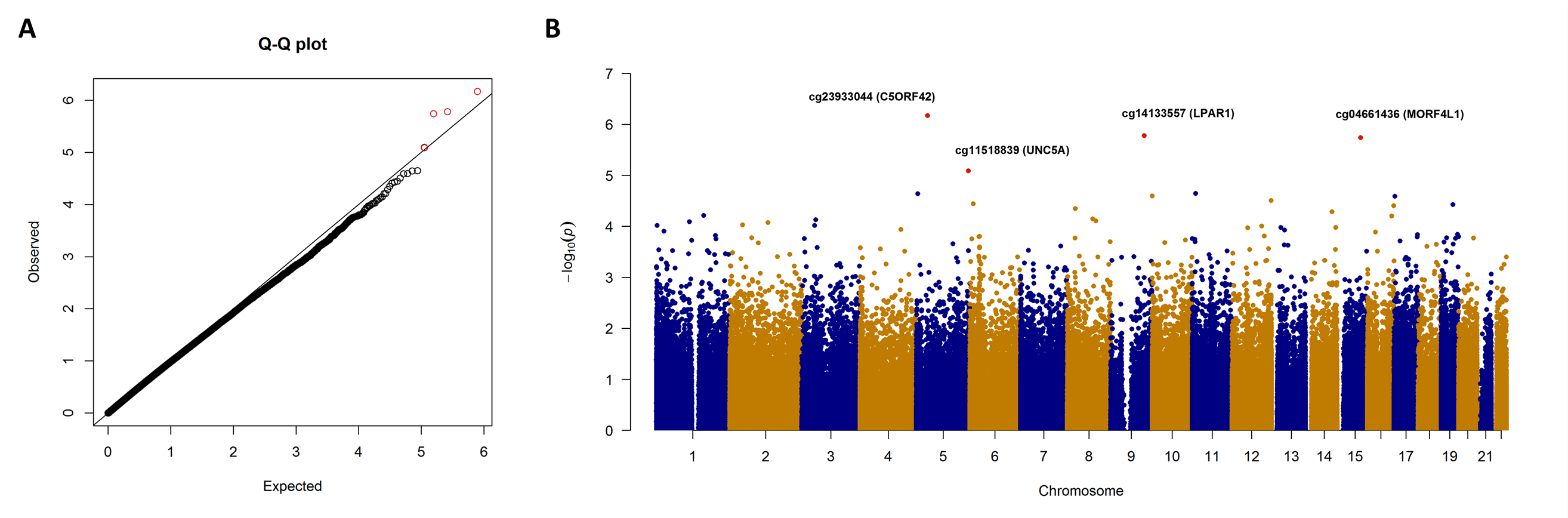
**

**Supplementary Figure 1. *A,*** Quantile-quantile (QQ) plot illustrating the -Log10(observed *p* value) (Observed) to the -Log10(expected *p* value) (Expected). ***B,*** Manhattan plot showing the distribution of *p* values from the paired comparison of 24 monozygotic twin pairs at age 10 who were discordant for psychotic symptoms at age 12, with the top 4 ranked differentially methylated positions (DMPs) shown in red and identified in the plot.

**Supplementary Table 1.** The top 100 ranked DMPs at age 10 in monozygotic twin pairs discordant for psychotic symptoms at age 12

| **Probe ID** | **Affected Twin Mean** | **Co-Twin Mean** | **Mean ∆β** | **P value** | **Hg19** | **Relation to CpG Island** | **Gene region feature category (UCSC)** | **SNPs in probe (+/- 10bp SBE)** | **Illumina Gene Annotation** | **Probe Type** | **Gene Annotation from GREAT (Distance from TSS)** |
| --- | --- | --- | --- | --- | --- | --- | --- | --- | --- | --- | --- |
| cg23933044 | 0.315 | 0.349 | -0.034 | 6.76E-07 | Chr5:37249909 | S_Shore | TSS1500 |  | C5ORF42 | II | C5ORF42 (-380) |
| cg14133557 | 0.796 | 0.742 | 0.055 | 1.65E-06 | Chr9:113802005 | S_Shore |  |  |  | II | LPAR1 (-1641) |
| cg04661436 | 0.795 | 0.751 | 0.044 | 1.81E-06 | Chr15:79169207 | S_Shelf | Body |  | MORF4L1 | II | MORF4L1 (+4036), CTSH (+68212) |
| cg11518839 | 0.378 | 0.428 | -0.051 | 8.11E-06 | Chr5:176216711 |  |  |  |  | II | UNC5A (-20848), TSPAN17 (+142324) |
| cg16508913 | 0.766 | 0.698 | 0.068 | 2.25E-05 | Chr11:11609419 |  | Body |  | GALNTL4 | II | CSNK2A1 (-234516), GALNTL4 (+34141) |
| cg19115205 | 0.899 | 0.873 | 0.026 | 2.28E-05 | Chr5:5062128 |  | Body | rs75560907 | LOC340094 | I | ADAMTS16 (-78314) |
| cg05764011 | 0.880 | 0.907 | -0.027 | 2.55E-05 | Chr10:409541 | S_Shore | Body |  | DIP2C | II | ZMYND11 (+183608), DIP2C (+326066) |
| cg26403608 | 0.308 | 0.396 | -0.088 | 2.58E-05 | Chr17:2319719 |  | TSS1500;3'UTR |  | LOC284009;METT10D | II | MNT (-15462), METTL16 (+95480) |
| cg04576398 | 0.747 | 0.700 | 0.047 | 3.10E-05 | Chr12:132262872 | N_Shore | Body |  | SFRS8 | II | MMP17 (-50068), SFSWAP (+67238) |
| cg16991886 | 0.903 | 0.888 | 0.015 | 3.58E-05 | Chr6:11832079 |  |  |  |  | I | HIVEP1 (-180644), C6orf105 (-52800) |
| cg08061902 | 0.848 | 0.818 | 0.030 | 3.71E-05 | Chr19:40169418 |  | TSS1500 |  | LOC400696 | II | LGALS14 (-25527), LGALS16 (+22861) |
| cg27418099 | 0.744 | 0.690 | 0.054 | 3.92E-05 | Chr16:88941395 | N_Shelf | 3'UTR |  | CBFA2T3 | II | PABPN1L (-8328), CBFA2T3 (+102108) |
| cg20546928 | 0.068 | 0.057 | 0.011 | 4.47E-05 | Chr8:27167985 | N_Shore | TSS1500;Body |  | PTK2B;TRIM35 | II | TRIM35 (+848) |
| cg06889165 | 0.604 | 0.556 | 0.048 | 5.18E-05 | Chr14:90042580 |  | Body;5'UTR |  | PRO1768;FOXN3 | II | FOXN3 (+42913), TTC8 (+751663) |
| cg12754671 | 0.366 | 0.391 | -0.025 | 6.12E-05 | Chr1:161171876 | Island | 5'UTR;TSS200 |  | NDUFS2 | I | ADAMTS4 (-3032), NDUFS2 (-60) |
| cg03804206 | 0.855 | 0.825 | 0.031 | 6.21E-05 | Chr16:82191819 |  | Body |  | MPHOSPH6 | I | MPHOSPH6 (+12009), HSD17B2 (+122962) |
| cg21211020 | 0.047 | 0.061 | -0.014 | 7.15E-05 | Chr8:86132685 | Island | TSS200 |  | C8orf59 | II | CA13 (-25030), E2F5 (+43067) |
| cg04602043 | 0.866 | 0.831 | 0.034 | 7.35E-05 | Chr3:47827617 | S_Shelf |  |  |  | II | SMARCC1 (-4213) |
| cg13160891 | 0.738 | 0.790 | -0.052 | 7.77E-05 | Chr8:96768366 |  |  |  |  | II | GDF6 (+404653), PLEKHF2 (+622418) |
| cg21304211 | 0.262 | 0.275 | -0.014 | 8.12E-05 | Chr1:112281877 | Island | Body;1stExon;5'UTR |  | C1orf183 | I | DDX20 (-16312), RAP1A (+119473) |
| cg21875839 | 0.850 | 0.815 | 0.035 | 8.39E-05 | Chr2:129308196 |  |  |  |  | II | HS6ST1 (-232026) |
| cg03181376 | 0.132 | 0.147 | -0.015 | 9.34E-05 | Chr2:43454447 | Island | TSS1500;Body |  | ZFP36L2;LOC100129726 | I | ZFP36L2 (-703) |
| cg25512936 | 0.173 | 0.198 | -0.025 | 9.57E-05 | Chr3:44041103 | Island |  |  |  | II | C3orf23 (-338840), ABHD5 (+308729) |
| cg12857678 | 0.785 | 0.725 | 0.060 | 9.60E-05 | Chr1:3549732 |  | Body |  | WDR8 | II | TPRG1L (+8177), WRAP73 (+16938) |
| cg14893473 | 0.649 | 0.593 | 0.056 | 9.88E-05 | Chr12:100041071 |  | Body;TSS1500 |  | ANKS1B | II | ANKS1B (+337360) |
| cg23470196 | 0.873 | 0.842 | 0.031 | 0.00010 | Chr14:102695671 | Island | Body |  | RAGE | II | HSP90AA1 (-89586), MOK (+75859) |
| cg06770877 | 0.823 | 0.788 | 0.035 | 0.00011 | Chr13:31897247 |  | Body |  | B3GALTL | II | RXFP2 (-416431), B3GALTL (+123136) |
| cg10227191 | 0.059 | 0.067 | -0.008 | 0.00011 | Chr12:53342746 | N_Shore | TSS200;5'UTR |  | KRT18 | II | KRT18 (-96) |
| cg13443950 | 0.686 | 0.736 | -0.050 | 0.00011 | Chr4:138584567 |  |  |  |  | II | PCDH18 (-130939), SLC7A11 (+578935) |
| cg08261525 | 0.221 | 0.281 | -0.060 | 0.00012 | Chr13:44239135 |  | 5'UTR |  | ENOX1 | II | SERP2 (-708842), ENOX1 (-35523) |
| cg03987192 | 0.352 | 0.395 | -0.043 | 0.00012 | Chr1:26373407 | S_Shore | TSS1500 |  | SLC30A2 | II | SLC30A2 (-804) |
| cg14431699 | 0.101 | 0.079 | 0.021 | 0.00013 | Chr16:27121094 | Island |  |  |  | II | JMJD5 (-94201) |
| cg08199563 | 0.803 | 0.763 | 0.041 | 0.00014 | Chr19:55816212 | Island | Body |  | BRSK1 | II | TMEM150B (+20495), BRSK1 (+20679) |
| cg06919800 | 0.890 | 0.909 | -0.019 | 0.00014 | Chr17:78803320 |  | Body |  | RPTOR | II | CHMP6 (-162320), RPTOR (+284696) |
| cg16028336 | 0.880 | 0.852 | 0.028 | 0.00015 | Chr1:200194941 |  |  |  |  | II | FAM58BP (+12286), ZNF281 (+184224) |
| cg00095930 | 0.287 | 0.335 | -0.048 | 0.00015 | Chr12:109569116 |  |  |  |  | II | ACACB (-8085), UNG (+33718) |
| cg07025169 | 0.884 | 0.858 | 0.026 | 0.00016 | Chr6:33236905 | N_Shelf | Body |  | VPS52 | II | RPS18 (-2946), VPS52 (+2756) |
| cg07061692 | 0.073 | 0.084 | -0.011 | 0.00016 | Chr19:59010811 | Island | Body |  | SLC27A5 | II | SLC27A5 (+12620), ZNF446 (+23017) |
| cg16880210 | 0.771 | 0.741 | 0.030 | 0.00016 | Chr6:31130290 | S_Shelf | Body | rs77870954 | TCF19 | I | CCHCR1 (-4725), TCF19 (+3988) |
| cg07428200 | 0.922 | 0.888 | 0.034 | 0.00016 | Chr17:78046742 |  | Body |  | CCDC40 | I | GAA (-28612), CCDC40 (+36312) |
| cg24836583 | 0.157 | 0.130 | 0.027 | 0.00017 | Chr19:19051747 | Island | TSS1500;5'UTR |  | HOMER3 | I | HOMER3 (+293) |
| cg03820024 | 0.077 | 0.091 | -0.015 | 0.00017 | Chr2:74649460 | S_Shore | Body |  | WDR54 | II | C2orf81 (-4617) |
| cg11903133 | 0.144 | 0.163 | -0.019 | 0.00017 | Chr20:50722613 | S_Shore | Body |  | ZFP64 | I | SALL4 (-303566), ZFP64 (+85910) |
| cg12252412 | 0.908 | 0.886 | 0.021 | 0.00017 | Chr8:26513782 |  | 3'UTR |  | DPYSL2 | II | DPYSL2 (+142074), ADRA1A (+209139) |
| cg00419186 | 0.105 | 0.096 | 0.010 | 0.00017 | Chr19:48825265 | Island |  |  |  | I | EMP3 (-3363) |
| cg17114283 | 0.083 | 0.071 | 0.012 | 0.00017 | Chr11:696207 | Island | Body;TSS1500 |  | TMEM80;DEAF1 | II | DEAF1 (-468), TMEM80 (+592) |
| cg12353927 | 0.851 | 0.825 | 0.026 | 0.00017 | Chr3:9814514 | S_Shelf |  |  |  | II | CAMK1 (-2847) |
| cg07915528 | 0.123 | 0.141 | -0.019 | 0.00018 | Chr1:202936986 | S_Shore | TSS1500 |  | CYB5R1 | I | CYB5R1 (-583) |
| cg13323047 | 0.097 | 0.107 | -0.011 | 0.00018 | Chr6:7261517 | Island |  |  |  | II | SSR1 (+52023), RREB1 (+153432) |
| cg00186954 | 0.802 | 0.751 | 0.051 | 0.00018 | Chr11:8933980 | S_Shore | TSS1500;Body |  | ST5;C11orf17 | II | AKIP1 (+1280), ASCL3 (+30599) |
| cg05318275 | 0.811 | 0.772 | 0.039 | 0.00019 | Chr10:112794609 |  |  | rs58174822 |  | II | ADRA2A (-42180), SHOC2 (+115309) |
| cg26791905 | 0.060 | 0.071 | -0.010 | 0.00019 | Chr1:120254664 | N_Shore | 1stExon |  | PHGDH | II | PHGDH (+246) |
| cg25819816 | 0.737 | 0.673 | 0.064 | 0.00019 | Chr17:20076850 |  | Body |  | CYTSB | II | SPECC1 (+164202), LGALS9B (+293997) |
| cg21310731 | 0.978 | 0.974 | 0.004 | 0.00020 | Chr8:145618932 | Island | Body |  | CPSF1 | I | CPSF1 (+15800), ADCK5 (+21202) |
| cg21949229 | 0.768 | 0.722 | 0.045 | 0.00020 | Chr11:9529385 |  | Body |  | ZNF143 | I | WEE1 (-65842), ZNF143 (+46874) |
| cg06622468 | 0.237 | 0.254 | -0.017 | 0.00020 | Chr19:10491460 | Island | TSS1500 |  | TYK2 | I | TYK2 (-213) |
| cg01291590 | 0.634 | 0.590 | 0.044 | 0.00021 | Chr10:42646111 | Island |  | rs34948030 |  | II | ZNF33B (+487880) |
| cg01310600 | 0.090 | 0.099 | -0.009 | 0.00021 | Chr2:96987238 | Island |  |  |  | I | ITPRIPL1 (-4706) |
| cg18368265 | 0.919 | 0.900 | 0.018 | 0.00022 | Chr5:123968247 |  |  |  |  | II | ZNF608 (+112617) |
| cg25237016 | 0.125 | 0.151 | -0.026 | 0.00022 | Chr19:41018757 | Island | Body |  | SPTBN4 | II | SHKBP1 (-63999), SPTBN4 (+45632) |
| cg21266547 | 0.853 | 0.873 | -0.020 | 0.00022 | Chr18:61654618 |  | 3'UTR;3'UTR |  | SERPINB8 | II | SERPINB8 (+17356), LINC00305 (+111255) |
| cg00131557 | 0.834 | 0.796 | 0.038 | 0.00023 | Chr13:43596656 | N_Shore | TSS1500 |  | DNAJC15 | II | DNAJC15 (-705) |
| cg20621674 | 0.698 | 0.645 | 0.053 | 0.00023 | Chr13:56188634 |  |  |  |  | II | NONE |
| cg08263941 | 0.715 | 0.660 | 0.054 | 0.00024 | Chr7:136401170 |  |  |  |  | II | MTPN (-738967), CHRM2 (-152661) |
| cg14482684 | 0.043 | 0.038 | 0.005 | 0.00024 | Chr18:30051176 | Island | TSS1500 |  | FAM59A | I | MEP1B (+281190), KLHL14 (+301797) |
| cg20729846 | 0.090 | 0.104 | -0.013 | 0.00025 | Chr6:32822182 | Island | Body;TSS1500 | rs4148878 | PSMB9;TAP1 | II | TAP1 (-435), PSMB9 (+245) |
| cg12365107 | 0.026 | 0.021 | 0.005 | 0.00026 | Chr3:52720035 | Island | 1stExon;5'UTR;TSS200 |  | GNL3;PBRM1 | I | GNL3 (+100) |
| cg18657303 | 0.885 | 0.857 | 0.028 | 0.00026 | Chr4:1051139 | S_Shore |  |  |  | II | FGFRL1 (+45380), RNF212 (+56212) |
| cg05826626 | 0.091 | 0.102 | -0.011 | 0.00026 | Chr6:32820862 | Island | TSS1500;1stExon |  | PSMB9;TAP1 | I | PSMB9 (-1075), TAP1 (+885) |
| cg23822643 | 0.845 | 0.863 | -0.018 | 0.00027 | Chr12:123341665 |  | Body |  | HIP1R | II | HIP1R (+21627), VPS37B (+39046) |
| cg17780992 | 0.052 | 0.059 | -0.007 | 0.00028 | Chr4:69215675 | Island | 1stExon;5'UTR |  | YTHDC1 | I | TMPRSS11B (-104264), TMPRSS11E (-97491) |
| cg06835156 | 0.873 | 0.835 | 0.038 | 0.00028 | Chr14:101131459 |  | Body |  | C14orf70 | II | BEGAIN (-97053), DLK1 (-61742) |
| cg11871280 | 0.864 | 0.828 | 0.036 | 0.00028 | Chr12:60082038 |  | TSS1500 |  | SLC16A7 | II | SLC16A7 (-1087) |
| cg22711777 | 0.065 | 0.072 | -0.007 | 0.00029 | Chr1:9352663 |  | TSS1500 |  | SPSB1 | I | SPSB1 (-277) |
| cg08093568 | 0.849 | 0.810 | 0.038 | 0.00029 | Chr7:27626595 |  | Body |  | HIBADH | II | HIBADH (+76024), EVX1 (+344432) |
| cg00394844 | 0.411 | 0.447 | -0.037 | 0.00030 | Chr19:18343302 | N_Shore | Body |  | PDE4C | II | RAB3A (-28429), PDE4C (+15707) |
| cg02624704 | 0.337 | 0.373 | -0.035 | 0.00030 | Chr1:173446429 | Island | TSS200 |  | PRDX6 | I | PRDX6 (-56) |
| cg04554817 | 0.645 | 0.582 | 0.062 | 0.00030 | Chr1:55508184 | S_Shelf | Body |  | PCSK9 | II | PCSK9 (+3036), USP24 (+172854) |
| cg11097675 | 0.646 | 0.702 | -0.056 | 0.00030 | Chr5:175612438 |  |  |  |  | II | THOC3 (-216894), KIAA1191 (+176370) |
| cg07315693 | 0.061 | 0.076 | -0.015 | 0.00030 | Chr11:118135203 |  | TSS200 |  | MPZL2;MPZL2 | II | MPZL2 (+47) |
| cg11058916 | 0.683 | 0.745 | -0.062 | 0.00031 | Chr16:34257749 | Island |  |  |  | II | BC068290 (+473239) |
| cg23709782 | 0.820 | 0.777 | 0.043 | 0.00031 | Chr15:99386939 |  | Body |  | IGF1R | II | PGPEP1L (+161945), IGF1R (+194179) |
| cg22396878 | 0.882 | 0.858 | 0.025 | 0.00031 | Chr4:151097199 |  | Body |  | DCLK2 | II | MAB21L2 (-405877), DCLK2 (+97774) |
| cg23405575 | 0.207 | 0.186 | 0.021 | 0.00032 | Chr3:11034281 | N_Shore | TSS200 |  | SLC6A1 | I | SLC6A1 (-138) |
| cg05925971 | 0.087 | 0.096 | -0.010 | 0.00033 | Chr19:36485966 | Island | TSS200 |  | SDHAF1 | I | SDHAF1 (-123) |
| cg20273697 | 0.094 | 0.107 | -0.013 | 0.00033 | Chr2:8977583 | Island | 5'UTR |  | KIDINS220 | I | KIDINS220 (+171) |
| cg02567344 | 0.944 | 0.936 | 0.009 | 0.00033 | Chr1:179010239 |  | 5'UTR |  | FAM20B | I | TOR3A (-40872), FAM20B (+15166) |
| cg03402805 | 0.060 | 0.066 | -0.006 | 0.00033 | Chr12:2921865 | Island | 1stExon;5'UTR |  | ITFG2 | I | FKBP4 (+17758), NRIP2 (+22355) |
| cg08455719 | 0.772 | 0.735 | 0.037 | 0.00034 | Chr1:234351342 | S_Shore | Body |  | SLC35F3 | II | C1orf31 (-158086), SLC35F3 (+310664) |
| cg18792689 | 0.639 | 0.602 | 0.037 | 0.00035 | Chr1:109254098 |  | TSS1500 | rs61797272 | FNDC7 | II | FNDC7 (-1457) |
| cg03345454 | 0.582 | 0.495 | 0.087 | 0.00035 | Chr2:202901428 | S_Shore | 1stExon;3'UTR |  | FZD7;FZD7 | II | FZD7 (+2119), SUMO1 (+201893) |
| cg08708599 | 0.098 | 0.107 | -0.010 | 0.00035 | Chr1:244893918 | Island |  |  |  | I | FAM36A (-104720), C1orf101 (+269246) |
| cg08831369 | 0.805 | 0.766 | 0.040 | 0.00036 | Chr1:197511177 |  | Body |  | DENND1B | II | DENND1B (+233445), CRB1 (+273844) |
| cg01890712 | 0.551 | 0.615 | -0.064 | 0.00036 | Chr11:57790918 |  | TSS1500 |  | OR9Q1 | II | OR9Q1 (-434) |
| cg17546147 | 0.774 | 0.755 | 0.019 | 0.00038 | Chr6:29710109 |  | Body |  | LOC285830 | II | LOC554223 (-49573), HLA-F (+18993) |
| cg00000321 | 0.381 | 0.434 | -0.053 | 0.00038 | Chr8:41167802 | S_Shore | TSS1500 |  | SFRP1 | II | SFRP1 (-813) |
| cg02494549 | 0.894 | 0.868 | 0.025 | 0.00039 | Chr2:161798364 |  |  |  |  | II | RBMS1 (-448047), TANK (-195101) |
| cg13482432 | 0.173 | 0.156 | 0.017 | 0.00039 | Chr9:79633350 | Island | TSS1500 |  | FOXB2 | I | FOXB2 (-1220) |
| cg03883640 | 0.860 | 0.816 | 0.044 | 0.00039 | Chr22:50727014 | N_Shore | Body |  | PLXNB2 | II | MAPK11 (-18236), PLXNB2 (+18986) |
| cg02017892 | 0.835 | 0.796 | 0.038 | 0.00040 | Chr8:103550807 |  |  |  |  | II | UBR5 (-125891), ODF1 (-13040) |
| cg10182151 | 0.878 | 0.836 | 0.042 | 0.00040 | Chr6:161575294 |  | Body |  | AGPAT4 | II | AGPAT4 (+119812), MAP3K4 (+162473) |
| cg14827481 | 0.731 | 0.687 | 0.045 | 0.00040 | Chr10:134361889 | S_Shore | Body | rs57835447 | INPP5A | II | INPP5A (+10537), NKX6-2 (+237647) |
| cg09375907 | 0.868 | 0.830 | 0.038 | 0.00040 | Chr6:49706534 |  | 5'UTR |  | CRISP3 | II | CRISP2 (-25236), CRISP3 (+5633) |
| cg08077354 | 0.924 | 0.907 | 0.017 | 0.00040 | Chr17:40437010 | N_Shelf |  |  |  | II | STAT5A (-2554) |
| cg14293614 | 0.308 | 0.264 | 0.043 | 0.00040 | Chr9:37593003 | S_Shore | TSS1500 |  | TOMM5 | II | TOMM5 (-368) |
| cg12044213 | 0.230 | 0.263 | -0.033 | 0.00041 | Chr6:31124978 | N_Shore | Body;TSS1500;5'UTR |  | CCHCR1;TCF19 | II | TCF19 (-1324), CCHCR1 (+587) |
| cg17332705 | 0.815 | 0.842 | -0.027 | 0.00041 | Chr4:7768162 |  | Body |  | AFAP1;LOC84740 | II | PSAPL1 (-331463), AFAP1 (+173490) |
| cg09867322 | 0.847 | 0.818 | 0.029 | 0.00041 | Chr5:135329478 |  |  | rs73294763 |  | II | LECT2 (-38756), TGFBI (-35105) |
| cg18507032 | 0.773 | 0.712 | 0.060 | 0.00042 | Chr11:61382092 |  | TSS1500 |  | RPLP0P2 | II | DAGLA (-65812), SYT7 (-33749) |
| cg10105699 | 0.898 | 0.881 | 0.017 | 0.00043 | Chr2:37869895 |  |  |  |  | II | CDC42EP3 (+29430), QPCT (+298143) |
| cg07224918 | 0.913 | 0.882 | 0.031 | 0.00043 | Chr1:17944423 |  | Body |  | ARHGEF10L | II | ACTL8 (-137384), ARHGEF10L (+78094) |
| cg27214721 | 0.082 | 0.094 | -0.012 | 0.00043 | Chr17:49337257 | Island | 1stExon;TSS1500;5'UTR |  | MBTD1;UTP18 | II | UTP18 (-639), MBTD1 (+169) |
| cg00994583 | 0.667 | 0.701 | -0.033 | 0.00044 | Chr17:36613498 | S_Shelf | TSS200 |  | ARHGAP23 | II | ARHGAP23 (+28779), SRCIN1 (+148684) |
| cg01409207 | 0.539 | 0.578 | -0.039 | 0.00046 | Chr12:117538129 | S_Shore | TSS1500 |  | TESC | II | TESC (-879) |
| cg14480858 | 0.041 | 0.045 | -0.004 | 0.00046 | Chr9:4666499 | S_Shelf | 1stExon;5'UTR |  | C9orf68 | I | CDC37L1 (-13066), PPAPDC2 (+4202) |
| cg02997817 | 0.886 | 0.868 | 0.018 | 0.00046 | Chr14:64929012 | N_Shelf |  |  |  | II | AKAP5 (-3204) |
| cg13697735 | 0.453 | 0.509 | -0.056 | 0.00046 | Chr14:65172103 | S_Shore | 5'UTR |  | PLEKHG3 | II | PLEKHG3 (-22207), HSPA2 (+164918) |
| cg22456479 | 0.758 | 0.706 | 0.051 | 0.00048 | Chr12:113793985 | N_Shelf |  |  |  | II | PLBD2 (-2385) |
| cg15669092 | 0.090 | 0.113 | -0.023 | 0.00048 | Chr16:89989024 | Island | TSS1500 |  | TUBB3 | II | TUBB3 (-662) |
| cg13883063 | 0.891 | 0.874 | 0.018 | 0.00048 | Chr9:138836839 |  | Body |  | UBAC1 | II | CAMSAP1 (-37835), UBAC1 (+16386) |
| cg25569234 | 0.047 | 0.057 | -0.010 | 0.00048 | Chr6:33257790 | S_Shore | Body;TSS1500 |  | PFDN6;WDR46 | I | WDR46 (-487), PFDN6 (+413) |
| cg19745903 | 0.749 | 0.684 | 0.065 | 0.00049 | Chr6:3129401 |  | Body |  | BPHL | II | BPHL (+10476), TUBB2A (+28381) |
| cg01236849 | 0.092 | 0.102 | -0.010 | 0.00049 | Chr11:16760157 | Island | 1stExon;5'UTR | rs34623871 | C11orf58 | II | C11orf58 (+10) |
| cg15340582 | 0.080 | 0.069 | 0.011 | 0.00050 | Chr5:139780509 | Island | TSS1500 |  | ANKHD1;EIF4EBP3 | II | ANKHD1-EIF4EBP3 (-889) |
| cg18602913 | 0.314 | 0.263 | 0.052 | 0.00050 | Chr19:16022934 | Island |  |  |  | II | CYP4F2 (-14051), CYP4F11 (+22741) |
| cg15340582 | 0.069 | 0.080 | 0.011 | 0.00050 | Chr5:139780509 | Island | TSS1500 |  | ANKHD1;ANKHD1-EIF4EBP3 | II | ANKHD1-EIF4EBP3 (-889) |
| cg18602913 | 0.263 | 0.314 | 0.052 | 0.00050 | Chr19:16022934 | Island |  |  |  | II | CYP4F2 (-14051), CYP4F11 (+22741) |
| cg00615915 | 0.063 | 0.073 | 0.010 | 0.00051 | Chr18:61637299 | N_Shore | 5'UTR;1stExon |  | SERPINB8 | II | SERPINB8 (+37) |
| cg15050310 | 0.875 | 0.844 | -0.032 | 0.00051 | Chr11:86617582 | Island |  |  |  | II | FZD4 (+48857), PRSS23 (+106092) |
| cg04462209 | 0.078 | 0.068 | -0.009 | 0.00051 | Chr1:202896493 | Island | TSS200 |  | KLHL12 | II | KLHL12 (-123) |
| cg15885786 | 0.693 | 0.743 | 0.050 | 0.00051 | Chr10:54054127 |  | 3'UTR |  | PRKG1;PRKG1 | II | CSTF2T (-594773), DKK1 (-19913) |
| cg20060394 | 0.813 | 0.837 | 0.024 | 0.00052 | Chr1:34328648 |  | 5'UTR;Body;TSS1500 |  | HMGB4;CSMD2 | II | HMGB4 (+2573), CSMD2 (+302226) |
| cg11855409 | 0.776 | 0.810 | 0.034 | 0.00052 | Chr7:14380781 |  | Body |  | DGKB | II | ETV1 (-351140), DGKB (+500293) |
| cg05409419 | 0.548 | 0.608 | 0.060 | 0.00052 | Chr14:30953702 |  |  |  |  | II | PRKD1 (-556804), G2E3 (-74626) |
| cg03310242 | 0.239 | 0.207 | -0.033 | 0.00052 | Chr12:2903563 | Island | TSS1500 |  | FKBP4 | I | FKBP4 (-544) |
| cg09361958 | 0.819 | 0.851 | 0.032 | 0.00053 | Chr3:128947790 |  |  |  |  | II | CNBP (-44981), COPG (-20662) |
| cg21126707 | 0.774 | 0.708 | -0.066 | 0.00054 | Chr12:81111012 | Island | 1stExon |  | MYF5 | II | MYF5 (+305) |
| cg14303948 | 0.130 | 0.120 | -0.010 | 0.00054 | Chr7:43878684 |  |  |  |  | I | MRPS24 (+30460), BLVRA (+80413) |
| cg23631932 | 0.549 | 0.507 | -0.043 | 0.00054 | Chr8:52322341 | S_Shore | Body |  | PXDNL | II | PXDNL (+399663) |
| cg03715152 | 0.067 | 0.061 | -0.006 | 0.00055 | Chr17:46969953 | Island | TSS200 |  | ATP5G1 | I | ATP5G1 (-194) |
| cg16519433 | 0.956 | 0.962 | 0.006 | 0.00055 | Chr10:3143596 | N_Shelf | Body |  | PFKP | I | PFKP (+33885), PITRM1 (+71436) |
| cg19533294 | 0.459 | 0.394 | -0.066 | 0.00055 | Chr4:87857667 | S_Shore | 5'UTR |  | AFF1 | II | AFF1 (+1514), HSD17B13 (+386388) |
| cg02377690 | 0.487 | 0.455 | -0.032 | 0.00055 | Chr6:37400392 | N_Shore | TSS1500 |  | FTSJD2 | II | FTSJD2 (-514) |
| cg04692538 | 0.570 | 0.602 | 0.031 | 0.00055 | Chr22:43586097 | S_Shelf |  |  |  | II | TTLL12 (-2961) |
| cg07551054 | 0.977 | 0.982 | 0.005 | 0.00056 | Chr8:140744200 | Island | Body |  | TRAPPC9 | I | KCNK9 (-28902), TRAPPC9 (+724477) |
| cg07050192 | 0.877 | 0.898 | 0.021 | 0.00056 | Chr10:22038263 |  |  |  |  | II | MLLT10 (+214690), DNAJC1 (+254386) |
| cg03097111 | 0.087 | 0.102 | 0.015 | 0.00057 | Chr10:99078896 | Island | TSS200 |  | FRAT1 | I | FRAT1 (-125) |
| cg23138461 | 0.060 | 0.048 | -0.012 | 0.00057 | Chr10:17685950 | N_Shore | TSS200 |  | STAM | II | STAM (-173) |
| cg15975890 | 0.354 | 0.313 | -0.041 | 0.00058 | Chr5:11903145 | N_Shore | Body |  | CTNND2 | II | CTNND2 (+964) |
| cg25450121 | 0.235 | 0.222 | -0.014 | 0.00058 | Chr17:46970175 | S_Shore | 1stExon;5'UTR | rs66985080 | ATP5G1 | II | ATP5G1 (+28) |
| cg06954520 | 0.735 | 0.781 | 0.046 | 0.00058 | Chr18:68095624 | N_Shelf |  |  |  | II | SOCS6 (+139488) |
| cg10700334 | 0.123 | 0.109 | -0.014 | 0.00058 | Chr3:118753610 | Island | 5'UTR;1stExon;Body |  | IGSF11 | II | IGSF11 (+65) |
| cg14523898 | 0.449 | 0.380 | -0.069 | 0.00058 | Chr1:38220138 | S_Shore | Body |  | EPHA10 | II | EPHA10 (+10685), CDCA8 (+61980) |
| cg17926940 | 0.097 | 0.074 | -0.023 | 0.00059 | Chr14:97685060 | Island |  |  |  | II | VRK1 (+421377) |
| cg20786876 | 0.712 | 0.753 | 0.041 | 0.00059 | Chr2:1938415 | N_Shelf | Body |  | MYT1L | II | PXDN (-190125), MYT1L (+396629) |
| cg16960573 | 0.889 | 0.910 | 0.021 | 0.00060 | Chr10:131200784 |  |  |  |  | I | MGMT (-64669) |
| cg08940097 | 0.979 | 0.983 | 0.004 | 0.00060 | Chr17:74094040 |  | Body |  | EXOC7 | I | EXOC7 (+5827), ZACN (+18778) |
| cg18196374 | 0.818 | 0.853 | 0.035 | 0.00060 | Chr3:196243502 |  | TSS1500 |  | C3orf43 | II | C3orf43 (-1266) |
| cg13956932 | 0.835 | 0.880 | 0.045 | 0.00060 | Chr2:3493521 |  |  |  |  | II | ADI1 (+29828), TRAPPC12 (+110076) |
| cg08107701 | 0.121 | 0.111 | -0.010 | 0.00060 | Chr2:218933668 |  | TSS200 |  | RUFY4 | II | RUFY4 (-69) |
| cg01447322 | 0.076 | 0.092 | 0.016 | 0.00062 | Chr11:66035600 | N_Shore | TSS1500 |  | RAB1B | II | RAB1B (-455) |
| cg06805253 | 0.070 | 0.080 | 0.010 | 0.00062 | Chr3:15900513 | Island | Body |  | ANKRD28 | I | ANKRD28 (+539) |
| cg12658052 | 0.692 | 0.743 | 0.051 | 0.00062 | Chr1:1078295 |  |  |  |  | II | TTLL10 (-30990), C1orf159 (-26560) |
| cg25488160 | 0.822 | 0.860 | 0.038 | 0.00062 | Chr3:133097868 |  | Body |  | TMEM108 | II | BFSP2 (-20921), TMEM108 (+340698) |
| cg03393769 | 0.891 | 0.909 | 0.018 | 0.00062 | Chr2:54611906 |  |  |  |  | I | TSPYL6 (-128498), SPTBN1 (-71547) |
| cg03716590 | 0.738 | 0.781 | 0.043 | 0.00062 | Chr7:95435309 |  | Body |  | DYNC1I1 | II | DYNC1I1 (+33492), SLC25A13 (+516149) |
| cg08591761 | 0.172 | 0.156 | -0.016 | 0.00063 | Chr19:57922319 | N_Shore | TSS1500 |  | ZNF17 | I | ZNF17 (-209) |
| cg19004134 | 0.078 | 0.069 | -0.009 | 0.00064 | Chr6:142468346 | Island | TSS200 |  | VTA1 | I | VTA1 (-63) |
| cg21503582 | 0.133 | 0.122 | -0.011 | 0.00064 | Chr7:151001017 | Island |  |  |  | I | SMARCD3 (-55269), NUB1 (-37829) |
| cg21843272 | 0.807 | 0.845 | 0.038 | 0.00065 | Chr12:48235729 |  | 3'UTR |  | VDR | II | HDAC7 (-21967), VDR (+63084) |
| cg13347970 | 0.160 | 0.113 | -0.047 | 0.00065 | Chr6:137809630 | Island |  |  |  | II | IFNGR1 (-269064), OLIG3 (+5900) |
| cg23394673 | 0.476 | 0.402 | -0.074 | 0.00065 | Chr1:1149211 | Island | Body |  | TNFRSF4 | I | TNFRSF4 (+336) |
| cg07203817 | 0.818 | 0.845 | 0.027 | 0.00066 | Chr1:64300033 |  | Body |  | ROR1 | II | UBE2U (-369456), ROR1 (+60344) |
| cg08439468 | 0.075 | 0.065 | -0.010 | 0.00066 | Chr9:95526812 | Island | 1stExon |  | BICD2 | II | BICD2 (+270) |
| cg06407434 | 0.685 | 0.626 | -0.059 | 0.00067 | Chr22:34046529 | Island | Body |  | LARGE | II | SYN3 (-643721), LARGE (+269886) |
| cg15444081 | 0.707 | 0.766 | 0.059 | 0.00068 | Chr7:123406500 |  |  |  |  | II | HYAL4 (-78722), WASL (-17385) |
| cg01780585 | 0.505 | 0.476 | -0.029 | 0.00068 | Chr1:36348359 | N_Shore | TSS1500 |  | EIF2C1 | I | EIF2C1 (-450) |
| cg15398152 | 0.267 | 0.322 | 0.055 | 0.00069 | Chr6:32016535 |  | Body |  | TNXB | II | CYP21A2 (+10443), TNXB (+60615) |
| cg14711016 | 0.112 | 0.100 | -0.012 | 0.00070 | Chr16:790766 | Island | Body |  | NARFL | II | NARFL (+230) |
| cg07887753 | 0.770 | 0.805 | 0.036 | 0.00071 | Chr8:57817338 |  |  |  |  | II | PENK (-458746), IMPAD1 (+89091) |
| cg24250374 | 0.822 | 0.854 | 0.032 | 0.00071 | Chr1:184591081 |  | 3'UTR |  | C1orf21 | I | EDEM3 (+132959), TSEN15 (+570271) |
| cg22765829 | 0.620 | 0.665 | 0.045 | 0.00071 | Chr6:168216358 |  |  |  |  | II | TCP10 (-418361), MLLT4 (-11312) |
| cg04569202 | 0.855 | 0.819 | -0.036 | 0.00072 | Chr7:157444423 | S_Shore | Body |  | PTPRN2 | II | DNAJB6 (+314714), PTPRN2 (+936058) |
| cg14368406 | 0.720 | 0.756 | 0.035 | 0.00072 | Chr7:148923027 | Island | 3'UTR |  | ZNF282 | II | ZNF212 (-13714), ZNF282 (+30451) |
| cg01776691 | 0.809 | 0.845 | 0.037 | 0.00072 | Chr7:44279727 | S_Shore | Body |  | CAMK2B | I | YKT6 (+39150), CAMK2B (+85502) |
| cg20529923 | 0.250 | 0.208 | -0.042 | 0.00072 | Chr11:32110187 | N_Shelf |  |  |  | II | RCN1 (-2289) |
| cg19478105 | 0.786 | 0.836 | 0.050 | 0.00072 | Chr10:98946943 | S_Shore | TSS1500 |  | SLIT1 | II | SLIT1 (-1261) |
| cg20121258 | 0.859 | 0.880 | 0.021 | 0.00072 | Chr11:119652442 |  |  |  |  | II | PVRL1 (-53008), TRIM29 (+356420) |
| cg16876823 | 0.699 | 0.747 | 0.048 | 0.00072 | Chr19:2987082 |  | Body |  | TLE6 | II | TLE6 (+9547), TLE2 (+42082) |
| cg12900467 | 0.075 | 0.065 | -0.010 | 0.00073 | Chr13:21635665 | Island | 5'UTR;1stExon |  | LATS2 | II | LATS2 (+56) |
| cg27492102 | 0.622 | 0.676 | 0.054 | 0.00073 | Chr13:26759970 | N_Shore |  |  |  | II | SHISA2 (-134773), RNF6 (+36537) |
| cg16953064 | 0.075 | 0.067 | -0.009 | 0.00074 | Chr16:72127243 | Island | 5'UTR;TSS1500 |  | DHX38;TXNL4B | II | DHX38 (-371), TXNL4B (+524) |
| cg04290835 | 0.821 | 0.857 | 0.036 | 0.00075 | Chr17:5350284 | S_Shelf | Body |  | DHX33 | II | C1QBP (-7814), DHX33 (+22095) |
| cg21396956 | 0.735 | 0.779 | 0.044 | 0.00075 | Chr18:56340167 | S_Shore | Body |  | MALT | II | ZNF532 (-189893), MALT1 (+1550) |
| cg16561957 | 0.917 | 0.934 | 0.017 | 0.00076 | Chr14:103430940 | S_Shore | Body |  | CDC42BPB | I | AMN (+41948), CDC42BPB (+92801) |
| cg05419798 | 0.677 | 0.719 | 0.042 | 0.00077 | Chr17:74928552 | N_Shore | Body |  | MGAT5B | II | SEC14L1 (-208452), MGAT5B (+63755) |
| cg16139011 | 0.095 | 0.086 | -0.010 | 0.00078 | Chr11:9596224 | Island | TSS200;Body |  | WEE1 | II | WEE1 (+997) |
| cg20202881 | 0.096 | 0.085 | -0.011 | 0.00078 | Chr17:57784779 | Island | TSS200;5'UTR;1stExon |  | TMEM49;PTRH2 | I | VMP1 (-83), PTRH2 (+76) |
| cg18630756 | 0.632 | 0.595 | -0.038 | 0.00079 | Chr10:107460450 |  |  |  |  | II | NONE |
| cg21068030 | 0.749 | 0.811 | 0.063 | 0.00079 | Chr17:7762413 | S_Shore | Body;TSS1500 |  | CYB5D1;LSMD1 | II | LSMD1 (-1242) |
| cg14987769 | 0.248 | 0.213 | -0.035 | 0.00079 | Chr2:220197576 | S_Shore | Body |  | RESP18 | II | RESP18 (+322) |
| cg08234149 | 0.690 | 0.648 | -0.042 | 0.00079 | Chr14:22925659 |  |  |  |  | II | TCRDV2 (-2429) |
| cg24449706 | 0.669 | 0.709 | 0.040 | 0.00079 | Chr8:25229721 |  | Body |  | DOCK5 | II | GNRH1 (+52834), DOCK5 (+187435) |
| cg14711428 | 0.081 | 0.069 | -0.012 | 0.00080 | Chr7:112090348 | Island | TSS200;5'UTR |  | IFRD1 | I | C7orf53 (-30559), IFRD1 (+27150) |
| cg07237214 | 0.082 | 0.074 | -0.008 | 0.00080 | Chr5:42424604 | Island | 5'UTR |  | GHR | I | GHR (+728) |
| cg15445958 | 0.687 | 0.656 | -0.031 | 0.00080 | Chr6:110266125 |  |  |  |  | II | GPR6 (-34172), FIG4 (+253702) |
| cg18415382 | 0.838 | 0.895 | 0.057 | 0.00081 | Chr10:135012575 | N_Shelf | Body |  | KNDC1 | I | UTF1 (-31202), KNDC1 (+38605) |
| cg25484904 | 0.252 | 0.212 | -0.040 | 0.00081 | Chr4:48988015 | Island | TSS1500 |  | CWH43 | II | CWH43 (-249) |
| cg09100988 | 0.088 | 0.103 | 0.015 | 0.00082 | Chr11:66725961 | Island | TSS200 |  | PC | I | SYT12 (-64854), PC (-50622) |
| cg01969473 | 0.772 | 0.813 | 0.041 | 0.00083 | Chr2:102978461 |  | TSS1500 |  | IL18R1 | II | IL18R1 (-631) |
| cg00742472 | 0.343 | 0.360 | 0.017 | 0.00083 | Chr14:31889912 | Island |  |  |  | I | HEATR5A (-125) |
| cg11998425 | 0.159 | 0.141 | -0.017 | 0.00083 | Chr19:8213583 | N_Shore | TSS1500 | rs3813771 | FBN3 | II | FBN3 (-1203) |
| cg14256643 | 0.809 | 0.830 | 0.020 | 0.00084 | Chr2:224896241 |  | TSS200;5'UTR;Body |  | SERPINE2 | II | SERPINE2 (+7794), MRPL44 (+74121) |
| cg20103018 | 0.790 | 0.825 | 0.034 | 0.00084 | Chr6:33996522 | S_Shore | Body |  | GRM4 | II | MLN (-224730), GRM4 (+105113) |
| cg11923627 | 0.054 | 0.048 | -0.005 | 0.00085 | Chr18:55470401 | Island |  |  |  | I | ATP8B1 (-75) |
| cg19170009 | 0.636 | 0.681 | 0.045 | 0.00085 | Chr10:17277756 |  | Body |  | VIM | II | VIM (+7499), ST8SIA6 (+218497) |
| cg12333210 | 0.839 | 0.865 | 0.025 | 0.00085 | Chr6:136685884 | S_Shelf | Body |  | MAP7 | II | BCLAF1 (-74896), MAP7 (+161466) |
| cg02060434 | 0.845 | 0.869 | 0.024 | 0.00085 | Chr12:52473014 | N_Shore |  |  |  | II | C12orf44 (+9257), KRT80 (+112769) |
| cg08156927 | 0.873 | 0.896 | 0.022 | 0.00086 | Chr16:963866 | S_Shore | Body |  | LMF1 | II | GNG13 (-113134), LMF1 (+57117) |
| cg07385577 | 0.063 | 0.059 | -0.005 | 0.00086 | Chr21:47394015 | Island |  |  |  | I | COL6A1 (-7647), PCBP3 (+124141) |
| cg12606891 | 0.729 | 0.770 | 0.040 | 0.00087 | Chr1:7913872 |  | TSS1500 |  | UTS2;UTS2 | II | UTS2 (-308) |
| cg27607283 | 0.797 | 0.755 | -0.042 | 0.00087 | Chr5:15385796 |  |  |  |  | II | ANKH (-513910), FBXL7 (-114508) |
| cg08650251 | 0.872 | 0.892 | 0.020 | 0.00088 | Chr4:166030097 | N_Shelf | Body |  | TMEM192 | II | TMEM192 (+3926), TRIM60 (+76947) |
| cg24627332 | 0.202 | 0.165 | -0.037 | 0.00088 | Chr6:76059450 |  | Body |  | FILIP1 | I | SENP6 (-252171), TMEM30A (-64819) |
| cg08578568 | 0.721 | 0.677 | -0.044 | 0.00089 | Chr16:86492943 |  |  |  |  | II | FOXF1 (-51189), IRF8 (+560170) |
| cg14320358 | 0.887 | 0.863 | -0.024 | 0.00089 | Chr20:31397044 |  | 3'UTR |  | DNMT3B | II | MAPRE1 (-10654), DNMT3B (+46854) |
| cg22736280 | 0.075 | 0.067 | -0.008 | 0.00089 | Chr8:95565249 | N_Shore | Body |  | KIAA1429 | I | KIAA1429 (+496) |
| cg26883033 | 0.430 | 0.499 | 0.069 | 0.00090 | Chr10:80027004 |  |  |  |  | II | ZMIZ1 (-801787), RPS24 (+233487) |
| cg08254399 | 0.764 | 0.794 | 0.031 | 0.00092 | Chr6:45671659 |  |  |  |  | II | SUPT3H (-325990), CLIC5 (+376425) |
| cg05573737 | 0.882 | 0.897 | 0.016 | 0.00092 | Chr10:134684065 | S_Shore |  |  |  | II | NKX6-2 (-84529), TTC40 (+72023) |
| cg11081580 | 0.121 | 0.105 | -0.017 | 0.00094 | Chr7:1286650 | Island |  |  |  | II | UNCX (+13997), MICALL2 (+212458) |
| cg11009335 | 0.114 | 0.095 | -0.020 | 0.00094 | Chr2:166651299 | S_Shore | TSS1500 |  | GALNT3 | I | GALNT3 (-497) |
| cg01359962 | 0.489 | 0.428 | -0.061 | 0.00095 | Chr3:43148002 | S_Shore | TSS1500 |  | C3orf39 | II | C3orf39 (-438) |
| cg08808677 | 0.853 | 0.822 | -0.031 | 0.00095 | Chr2:6911141 |  |  |  |  | II | CMPK2 (+94794) |
| cg03165383 | 0.780 | 0.810 | 0.030 | 0.00095 | Chr18:74981431 |  | 3'UTR |  | GALR1 | II | GALR1 (+19424) |
| cg17283347 | 0.072 | 0.066 | -0.006 | 0.00095 | Chr17:56736571 | N_Shore | 5'UTR |  | TEX14 | II | SEPT4 (-129794), TEX14 (+32844) |
| cg08118412 | 0.835 | 0.861 | 0.027 | 0.00095 | Chr3:64113418 |  | Body |  | PRICKLE2 | II | PSMD6 (-104299), PRICKLE2 (+97712) |
| cg22288011 | 0.711 | 0.749 | 0.038 | 0.00095 | Chr3:182881613 | S_Shore | TSS1500 | rs34992803 | LAMP3 | II | LAMP3 (-947) |
| cg10690515 | 0.803 | 0.838 | 0.035 | 0.00096 | Chr3:44751120 | N_Shelf |  |  |  | II | ZNF502 (-3014) |
| cg01914743 | 0.845 | 0.860 | 0.015 | 0.00096 | Chr19:9270907 | Island | Body |  | ZNF317 | II | OR7D2 (-25362), ZNF317 (+19852) |
| cg22557029 | 0.082 | 0.073 | -0.009 | 0.00096 | Chr7:45026411 | Island | TSS1500 |  | SNORA9;C7orf40 | II | CCM2 (-13375), MYO1G (-7708) |
| cg03854796 | 0.157 | 0.121 | -0.036 | 0.00096 | Chr11:31819162 | N_Shore | Body |  | PAX6 | II | PAX6 (+20346), ELP4 (+287866) |
| cg02483043 | 0.863 | 0.837 | -0.025 | 0.00097 | Chr10:134536498 | S_Shore | Body |  | INPP5A | II | NKX6-2 (+63038), INPP5A (+185146) |
| cg08285446 | 0.553 | 0.503 | -0.050 | 0.00097 | Chr8:1273856 |  |  |  |  | II | C8orf42 (-778526), DLGAP2 (-175712) |
| cg20084184 | 0.863 | 0.837 | -0.027 | 0.00097 | Chr4:1148524 |  |  |  |  | II | RNF212 (-41173), SPON2 (+18132) |
| cg13473086 | 0.787 | 0.817 | 0.030 | 0.00097 | Chr13:67676107 |  | Body |  | PCDH9 | II | PCDH9 (+128360) |
| cg15558616 | 0.604 | 0.659 | 0.055 | 0.00098 | Chr3:173630182 |  | Body |  | NLGN1 | II | NAALADL2 (-946928), NLGN1 (+513939) |
| cg19355190 | 0.175 | 0.132 | -0.042 | 0.00098 | Chr10:64575798 | Island | 5'UTR;1stExon |  | EGR2 | II | EGR2 (+327) |
| cg07594674 | 0.167 | 0.111 | -0.056 | 0.00099 | Chr2:47499812 | Island |  |  |  | II | EPCAM (-96474), CALM2 (-96073) |
| cg09784168 | 0.616 | 0.669 | 0.053 | 0.00099 | Chr7:111843174 | N_Shelf | Body |  | DOCK4 | II | ZNF277 (-3468), DOCK4 (+3287) |
| cg13948230 | 0.037 | 0.041 | 0.005 | 0.00099 | Chr10:126106957 | Island | 5'UTR |  | OAT | II | OAT (+587) |
| cg11046502 | 0.080 | 0.088 | 0.009 | 0.00100 | Chr8:27491495 | Island | TSS200 |  | SCARA3 | I | SCARA3 (-81) |

*Note.* Ranked by *p* value (p < 5e-4). ∆β, difference in DNA methylation; DMPs, differentially methylated positions; GREAT, Genomic Regions Enrichment of Annotations Tool; Hg19, Human Genome build 19; SBE, single-base extension; SNP, single nucleotide polymorphism; TSS, transcription start site.

**Supplementary Table 2.** Top ten ranked DMPs at age 10 adjusted for internalizing and externalizing problems at age 10 and depressive symptoms at age 12.

| **Probe ID** | **Mean ∆β** | **P value** |
| --- | --- | --- |
| cg23933044 | -0.037 | 1.74E-06 |
| cg14133557 | 0.051 | 2.22E-06 |
| cg04661436 | 0.042 | 6.47E-06 |
| cg11518839 | -0.047 | 7.48E-05 |
| cg16508913 | 0.065 | 4.72E-05 |
| cg19115205 | 0.027 | 4.90E-05 |
| cg05764011 | -0.024 | 0.00011 |
| cg26403608 | -0.082 | 6.80E-05 |
| cg04576398 | 0.045 | 6.55E-05 |
| cg16991886 | 0.014 | 0.00015 |

*Note.* ∆β, difference in DNA methylation; DMP, differentially methylated position.

**Supplementary Table 3.** Gene Ontology (GO) enrichment analysis of top ranked age 10 DMPs.

| **GO Accession ID** | **GO Function** | **Ontology** | **P value** |
| --- | --- | --- | --- |
| GO:0002335 | mature B cell differentiation | BP | 0.0002 |
| GO:0051966 | regulation of synaptic transmission, glutamatergic | BP | 0.0034 |
| GO:0017147 | Wnt-protein binding | MF | 0.0037 |
| GO:0042976 | activation of Janus kinase activity | BP | 0.0037 |
| GO:0071305 | cellular response to vitamin D | BP | 0.0043 |
| GO:0007163 | establishment or maintenance of cell polarity | BP | 0.0045 |
| GO:0051649 | establishment of localization in cell | BP | 0.0050 |
| GO:0016310 | phosphorylation | BP | 0.0054 |
| GO:0071295 | cellular response to vitamin | BP | 0.0055 |
| GO:0016773 | phosphotransferase activity, alcohol group as acceptor | MF | 0.0058 |
| GO:0031175 | neuron projection development | BP | 0.0064 |
| GO:0050804 | regulation of synaptic transmission | BP | 0.0064 |
| GO:0051168 | nuclear export | BP | 0.0067 |
| GO:0042220 | response to cocaine | BP | 0.0068 |
| GO:0017046 | peptide hormone binding | MF | 0.0069 |
| GO:0030165 | PDZ domain binding | MF | 0.0073 |
| GO:0016226 | iron-sulfur cluster assembly | BP | 0.0074 |
| GO:0031163 | metallo-sulfur cluster assembly | BP | 0.0074 |
| GO:0006107 | oxaloacetate metabolic process | BP | 0.0075 |
| GO:0033209 | tumor necrosis factor-mediated signaling pathway | BP | 0.0092 |
| GO:0016301 | kinase activity | MF | 0.0094 |
| GO:0004715 | non-membrane spanning protein tyrosine kinase activity | MF | 0.0116 |
| GO:0046907 | intracellular transport | BP | 0.0121 |
| GO:0032892 | positive regulation of organic acid transport | BP | 0.0125 |
| GO:0009081 | branched-chain amino acid metabolic process | BP | 0.0129 |
| GO:0004713 | protein tyrosine kinase activity | MF | 0.0134 |
| GO:0045664 | regulation of neuron differentiation | BP | 0.0136 |
| GO:0071356 | cellular response to tumor necrosis factor | BP | 0.0142 |
| GO:0051955 | regulation of amino acid transport | BP | 0.0143 |
| GO:0006641 | triglyceride metabolic process | BP | 0.0146 |
| GO:0007215 | glutamate receptor signaling pathway | BP | 0.0146 |
| GO:0051287 | NAD binding | MF | 0.0147 |
| GO:0004672 | protein kinase activity | MF | 0.0150 |
| GO:0071229 | cellular response to acid chemical | BP | 0.0150 |
| GO:0030098 | lymphocyte differentiation | BP | 0.0150 |
| GO:0006468 | protein phosphorylation | BP | 0.0157 |
| GO:0010975 | regulation of neuron projection development | BP | 0.0159 |
| GO:0007216 | G-protein coupled glutamate receptor signaling pathway | BP | 0.0167 |
| GO:0031503 | protein complex localization | BP | 0.0170 |
| GO:0045822 | negative regulation of heart contraction | BP | 0.0178 |
| GO:0033280 | response to vitamin D | BP | 0.0180 |
| GO:0090330 | regulation of platelet aggregation | BP | 0.0181 |
| GO:0031670 | cellular response to nutrient | BP | 0.0183 |
| GO:0017146 | N-methyl-D-aspartate selective glutamate receptor complex | CC | 0.0185 |
| GO:0008028 | monocarboxylic acid transmembrane transporter activity | MF | 0.0188 |
| GO:0006639 | acylglycerol metabolic process | BP | 0.0189 |
| GO:0006638 | neutral lipid metabolic process | BP | 0.0193 |
| GO:0034451 | centriolar satellite | CC | 0.0194 |
| GO:0016482 | cytoplasmic transport | BP | 0.0200 |
| GO:0048505 | regulation of timing of cell differentiation | BP | 0.0219 |
| GO:0042813 | Wnt-activated receptor activity | MF | 0.0222 |
| GO:0007271 | synaptic transmission, cholinergic | BP | 0.0222 |
| GO:1902582 | single-organism intracellular transport | BP | 0.0224 |
| GO:0048666 | neuron development | BP | 0.0229 |
| GO:0051954 | positive regulation of amine transport | BP | 0.0229 |
| GO:0040034 | regulation of development, heterochronic | BP | 0.0230 |
| GO:2000310 | regulation of N-methyl-D-aspartate selective glutamate receptor activity | BP | 0.0234 |
| GO:0035249 | synaptic transmission, glutamatergic | BP | 0.0236 |
| GO:0038083 | peptidyl-tyrosine autophosphorylation | BP | 0.0237 |
| GO:1901135 | carbohydrate derivative metabolic process | BP | 0.0240 |
| GO:0050767 | regulation of neurogenesis | BP | 0.0241 |
| GO:0050806 | positive regulation of synaptic transmission | BP | 0.0247 |
| GO:0033036 | macromolecule localization | BP | 0.0254 |
| GO:0005085 | guanyl-nucleotide exchange factor activity | MF | 0.0255 |
| GO:0034612 | response to tumor necrosis factor | BP | 0.0260 |
| GO:0051960 | regulation of nervous system development | BP | 0.0264 |
| GO:0042562 | hormone binding | MF | 0.0270 |
| GO:0042744 | hydrogen peroxide catabolic process | BP | 0.0272 |
| GO:0051968 | positive regulation of synaptic transmission, glutamatergic | BP | 0.0282 |
| GO:0006888 | ER to Golgi vesicle-mediated transport | BP | 0.0282 |
| GO:0030426 | growth cone | CC | 0.0284 |
| GO:0060999 | positive regulation of dendritic spine development | BP | 0.0294 |
| GO:0016772 | transferase activity, transferring phosphorus-containing groups | MF | 0.0297 |
| GO:0018108 | peptidyl-tyrosine phosphorylation | BP | 0.0298 |
| GO:0048103 | somatic stem cell division | BP | 0.0307 |
| GO:0018212 | peptidyl-tyrosine modification | BP | 0.0309 |
| GO:0030427 | site of polarized growth | CC | 0.0312 |
| GO:0060284 | regulation of cell development | BP | 0.0314 |
| GO:0030183 | B cell differentiation | BP | 0.0317 |
| GO:0008104 | protein localization | BP | 0.0320 |
| GO:0021683 | cerebellar granular layer morphogenesis | BP | 0.0333 |
| GO:0002028 | regulation of sodium ion transport | BP | 0.0335 |
| GO:0042659 | regulation of cell fate specification | BP | 0.0337 |
| GO:0031669 | cellular response to nutrient levels | BP | 0.0339 |
| GO:0002521 | leukocyte differentiation | BP | 0.0340 |
| GO:0034110 | regulation of homotypic cell-cell adhesion | BP | 0.0342 |
| GO:0032855 | positive regulation of Rac GTPase activity | BP | 0.0346 |
| GO:0006369 | termination of RNA polymerase II transcription | BP | 0.0347 |
| GO:0007270 | neuron-neuron synaptic transmission | BP | 0.0352 |
| GO:0030850 | prostate gland development | BP | 0.0358 |
| GO:1901605 | alpha-amino acid metabolic process | BP | 0.0359 |
| GO:0060359 | response to ammonium ion | BP | 0.0364 |
| GO:0009118 | regulation of nucleoside metabolic process | BP | 0.0364 |
| GO:0048708 | astrocyte differentiation | BP | 0.0370 |
| GO:0004683 | calmodulin-dependent protein kinase activity | MF | 0.0375 |
| GO:0021681 | cerebellar granular layer development | BP | 0.0393 |
| GO:0033218 | amide binding | MF | 0.0394 |
| GO:0071837 | HMG box domain binding | MF | 0.0395 |
| GO:0017112 | Rab guanyl-nucleotide exchange factor activity | MF | 0.0408 |
| GO:0022904 | respiratory electron transport chain | BP | 0.0418 |
| GO:0032526 | response to retinoic acid | BP | 0.0419 |
| GO:0042063 | gliogenesis | BP | 0.0425 |
| GO:0019637 | organophosphate metabolic process | BP | 0.0429 |
| GO:0048167 | regulation of synaptic plasticity | BP | 0.0436 |
| GO:0006120 | mitochondrial electron transport, NADH to ubiquinone | BP | 0.0447 |
| GO:0071300 | cellular response to retinoic acid | BP | 0.0451 |
| GO:0007398 | ectoderm development | BP | 0.0453 |
| GO:0046128 | purine ribonucleoside metabolic process | BP | 0.0453 |
| GO:0021510 | spinal cord development | BP | 0.0453 |
| GO:0022900 | electron transport chain | BP | 0.0454 |
| GO:0010656 | negative regulation of muscle cell apoptotic process | BP | 0.0455 |
| GO:0006968 | cellular defense response | BP | 0.0457 |
| GO:0009950 | dorsal/ventral axis specification | BP | 0.0460 |
| GO:0007622 | rhythmic behavior | BP | 0.0461 |
| GO:0019221 | cytokine-mediated signaling pathway | BP | 0.0467 |
| GO:0030279 | negative regulation of ossification | BP | 0.0467 |
| GO:0006903 | vesicle targeting | BP | 0.0471 |
| GO:0042278 | purine nucleoside metabolic process | BP | 0.0473 |
| GO:0043087 | regulation of GTPase activity | BP | 0.0474 |
| GO:0032319 | regulation of Rho GTPase activity | BP | 0.0479 |
| GO:0030010 | establishment of cell polarity | BP | 0.0479 |
| GO:0033124 | regulation of GTP catabolic process | BP | 0.0481 |
| GO:0051539 | 4 iron, 4 sulfur cluster binding | MF | 0.0485 |
| GO:0009205 | purine ribonucleoside triphosphate metabolic process | BP | 0.0485 |
| GO:0042113 | B cell activation | BP | 0.0486 |
| GO:0009119 | ribonucleoside metabolic process | BP | 0.0490 |
| GO:0009199 | ribonucleoside triphosphate metabolic process | BP | 0.0495 |
| GO:0001101 | response to acid chemical | BP | 0.0496 |

*Note.* BP, Biological Processes; CC, Cellular; DMPs, differentially methylated positions; MF, Molecular Function.

**Supplementary Table 4. Details of post-mortem prefrontal cortex brain samples.**

| **Sample** | **SZ** | **Control** | **Total** | ***P* value** |
| --- | --- | --- | --- | --- |
| LBBND |  |  |  |  |
| Number | 20 | 23 | 43 |  |
| Sex (male:female) | 11:09 | 17:06 | 28:15 | 0.19 |
| Age at death (years) | 62.1± 15.87 | 62 ± 18.74 | 62.05 ± 17.26 | 0.99 |
| Total brain weight (g) | 1359± 150 | 1465 ± 197.8 | 1420 ± 182 | 0.07 |
| pH | 6.64 ± 0.28 | 6.49 ± 0.33 | 6.56 ± 0.31 | 0.13 |
| DBCBB |  |  |  |  |
| Number | 18 | 15 | 33 |  |
| Sex (male:female) | 15:03 | 13:02 | 28:05 | 0.79 |
| Age at death (years) | 45.5 ± 16.6 | 42.3 ± 14.8 | 44.0 ± 15.7 | 0.28 |
| Total brain weight (g) | 1432 ± 188 | 1463 ± 175 | 1447 ± 179 | 0.32 |
| pH | 6.6 ± 0.28 | 6.48 ± 0.33 | 6.54 ± 0.31 | 0.13 |

*Note.* Schizophrenia (SZ) cases and controls are matched (Chi-Squared test [for sex] or t-test *P* value>0.05) for all variables. DBCBB, Douglas Bell-Canada Brain Bank; g, grams; LBBND, London Brain Bank for Neurodegenerative Disorders.

**Supplementary Table 5.** The top 100 ranked DMPs at age 5 for monozygotic twins discordant for psychotic symptoms at age 12

| **Probe ID** | **Affected Twin Mean** | **Co-Twin Mean** | **Mean ∆β** | **P value** | **Hg19** | **Relation to CpG Island** | **Gene region feature category (UCSC)** | **SNPs in probe (+/- 10bp SBE)** | **Illumina Gene Annotation** | **Probe Type** | **Gene Annotation from GREAT (Distance from TSS)** |
| --- | --- | --- | --- | --- | --- | --- | --- | --- | --- | --- | --- |
| cg15031661 | 0.150 | 0.130 | 0.020 | 1.26E-05 | Chr1:238323226 | Island | 1stExon |  | FMN2 | I | FMN2 (+1419), GREM2 (+518858) |
| cg26432347 | 0.082 | 0.063 | 0.018 | 1.87E-05 | Chr6:30818615 | Island | TSS200 |  | FLOT1 | I | FLOT1 (-184) |
| cg11356706 | 0.070 | 0.061 | 0.010 | 1.95E-05 | Chr20:604240 | Island | 1stExon |  | SCRT2 | I | SCRT2 (+582) |
| cg16011679 | 0.116 | 0.094 | 0.022 | 2.59E-05 | Chr1:85497983 | Island | TSS200 |  | C1orf52 | I | SYDE2 (-58668), BCL10 (+17191) |
| cg21480740 | 0.899 | 0.883 | 0.016 | 2.64E-05 | Chr7:158511954 | S_Shore |  |  |  | II | ESYT2 (-196875), VIPR2 (+118455) |
| cg10377582 | 0.443 | 0.520 | -0.077 | 3.03E-05 | Chr12:49899061 | S_Shore | TSS1500 |  | POU6F1 | I | POU6F1 (-20845), DAZAP2 (-19713) |
| cg24085426 | 0.863 | 0.839 | 0.024 | 3.05E-05 | Chr12:45869545 |  |  |  |  | II | AMIGO2 (-109545), FAM113B (-26726) |
| cg03044239 | 0.072 | 0.080 | -0.008 | 3.15E-05 | Chr3:139549743 | Island | 5'UTR;TSS1500;1stExon |  | MRAS | II | MRAS (-454) |
| cg06547771 | 0.044 | 0.052 | -0.008 | 3.34E-05 | Chr11:43336969 | Island | TSS200 |  | TTC17 | I | TTC17 (-41) |
| cg14659771 | 0.138 | 0.114 | 0.024 | 3.59E-05 | Chr2:231625606 | Island |  |  |  | I | PSMD1 (-4215) |
| cg24391460 | 0.525 | 0.462 | 0.062 | 4.12E-05 | Chr7:78922102 | S_Shore | TSS1500 |  | MAGI2 | I | MAGI2 (-1277) |
| cg24106824 | 0.927 | 0.918 | 0.009 | 5.24E-05 | Chr17:77284720 | Island | 3'UTR |  | MRPL12 | I | SLC25A10 (-5055), HGS (+23354) |
| cg21155461 | 0.048 | 0.042 | 0.006 | 5.57E-05 | Chr19:63432065 | Island | 5'UTR;1stExon |  | ZNF544 | I | ZNF544 (+184) |
| cg09501025 | 0.760 | 0.718 | 0.043 | 5.59E-05 | Chr10:72982961 |  | Body |  | CDH23 | II | CDH23 (+156265), C10orf105 (+166622) |
| cg12730562 | 0.779 | 0.727 | 0.052 | 6.27E-05 | Chr11:44884452 |  | 5'UTR;TSS200 |  | TSPAN18 | II | TP53I11 (+44731), TSPAN18 (+141901) |
| cg15648026 | 0.100 | 0.084 | 0.016 | 7.32E-05 | Chr11:106967008 | Island | TSS200;TSS1500 |  | ELMOD1 | I | ELMOD1 (-18) |
| cg08370347 | 0.799 | 0.841 | -0.041 | 8.64E-05 | Chr5:171504398 |  | Body |  | STK10 | II | FBXW11 (-137917), STK10 (+43552) |
| cg08307030 | 0.381 | 0.294 | 0.087 | 8.85E-05 | Chr15:74421435 | Island | 3'UTR |  | ISL2 | II | ISL2 (+5234), SCAPER (+541836) |
| cg10624784 | 0.166 | 0.179 | -0.012 | 9.08E-05 | Chr7:77265849 | Island | TSS200 |  | PHTF2;TMEM60 | I | PHTF2 (-195), TMEM60 (-167) |
| cg01353538 | 0.872 | 0.847 | 0.025 | 9.66E-05 | Chr6:43860500 |  | 3'UTR |  | VEGFA | II | VEGFA (+14577), MRPL14 (+342668) |
| cg00802617 | 0.938 | 0.927 | 0.012 | 9.77E-05 | Chr15:97489121 |  | Body |  | SYNM | II | SYNM (+26313), TTC23 (+118216) |
| cg17397135 | 0.082 | 0.073 | 0.009 | 0.00011 | Chr2:28643320 | Island | Body |  | PLB1 | I | PPP1CB (-184797), PLB1 (+70879) |
| cg13382000 | 0.047 | 0.040 | 0.007 | 0.00011 | Chr4:71772749 | Island | TSS1500 |  | UTP3 | I | UTP3 (-310) |
| cg04262465 | 0.733 | 0.778 | -0.045 | 0.00012 | Chr14:100475446 |  | TSS200 |  | SNORD113-6 | II | DIO3 (-621994), RTL1 (-54510) |
| cg11556416 | 0.308 | 0.262 | 0.046 | 0.00013 | Chr2:191587497 | Island | TSS1500 |  | STAT1 | I | STAT1 (-277) |
| cg10970392 | 0.884 | 0.910 | -0.026 | 0.00015 | Chr1:152809742 | N_Shore | Body |  | CHRNB2 | II | CHRNB2 (+2862), ADAR (+37605) |
| cg25523261 | 0.849 | 0.882 | -0.033 | 0.00016 | Chr2:37972475 |  |  |  |  | II | CDC42EP3 (-219646), FAM82A1 (-58624) |
| cg12493050 | 0.042 | 0.037 | 0.005 | 0.00016 | Chr6:31234065 | Island | TSS1500 |  | CCHCR1;TCF19 | I | CCHCR1 (-521), TCF19 (-216) |
| cg17806717 | 0.798 | 0.831 | -0.033 | 0.00017 | Chr6:32135497 |  | Body |  | TNXB | II | CYP21A2 (+21427), TNXB (+49631) |
| cg14344261 | 0.081 | 0.065 | 0.016 | 0.00018 | Chr9:36976380 | Island | Body |  | PAX5 | I | PAX5 (+48095), MELK (+413476) |
| cg12928619 | 0.040 | 0.049 | -0.009 | 0.00018 | Chr2:74502262 | Island | TSS200 |  | WDR54 | I | C2orf81 (-3911) |
| cg14014964 | 0.914 | 0.867 | 0.046 | 0.00019 | Chr9:93483513 |  |  |  |  | I | NFIL3 (-257549), ROR2 (+268751) |
| cg10113467 | 0.084 | 0.074 | 0.011 | 0.00019 | Chr13:74954491 | Island | TSS1500 |  | TBC1D4 | I | TBC1D4 (-241) |
| cg17025730 | 0.162 | 0.136 | 0.026 | 0.00019 | Chr5:133887697 | N_Shore |  |  |  | I | PHF15 (-1999) |
| cg01314143 | 0.059 | 0.049 | 0.010 | 0.00019 | Chr5:1852486 | Island | 5'UTR |  | MRPL36 | I | NDUFS6 (-2009), MRPL36 (+469) |
| cg00422638 | 0.272 | 0.211 | 0.061 | 0.00020 | Chr10:25506033 | S_Shore | TSS1500;Body |  | GPR158 | II | MYO3A (-756974), GPR158 (+1738) |
| cg10539936 | 0.894 | 0.862 | 0.032 | 0.00020 | Chr14:69569738 |  |  |  |  | II | SMOC1 (+153872), SLC8A3 (+155801) |
| cg12667002 | 0.727 | 0.780 | -0.053 | 0.00020 | Chr13:113830404 | N_Shore | Body |  | RASA3 | II | GAS6 (-245257), RASA3 (+85792) |
| cg14837825 | 0.905 | 0.881 | 0.024 | 0.00021 | Chr2:96281294 |  | 3'UTR |  | TMEM127 | II | STARD7 (-42995), TMEM127 (+14183) |
| cg08550421 | 0.087 | 0.123 | -0.036 | 0.00021 | Chr5:140286131 | Island | Body;TSS1500 |  | PCDHA1-12 | II | PCDHAC2 (-39799), ZMAT2 (+225916) |
| cg01964795 | 0.901 | 0.874 | 0.027 | 0.00022 | Chr16:2182897 | N_Shelf | Body |  | CASKIN1 | II | CASKIN1 (+3568), TRAF7 (+37098) |
| cg24324984 | 0.626 | 0.676 | -0.050 | 0.00022 | Chr19:51110894 | S_Shore | TSS1500 |  | NANOS2 | II | NANOS2 (-1019) |
| cg03357540 | 0.062 | 0.054 | 0.008 | 0.00022 | Chr18:17434821 | Island | TSS200 |  | ESCO1 | I | ESCO1 (-131) |
| cg01684006 | 0.892 | 0.870 | 0.022 | 0.00022 | Chr11:129518567 |  | Body;3'UTR |  | APLP2 | II | ST14 (-16324), APLP2 (+72895) |
| cg25053907 | 0.120 | 0.099 | 0.022 | 0.00023 | Chr4:482859 | Island | 5'UTR;TSS200;TSS200;1stExon |  | ZNF721;PIGG | I | PIGG (-129), ZNF721 (+582) |
| cg17802633 | 0.734 | 0.676 | 0.059 | 0.00023 | Chr2:68724972 | S_Shore | TSS1500 |  | PROKR1 | I | PROKR1 (-1325) |
| cg04881131 | 0.224 | 0.263 | -0.039 | 0.00023 | Chr20:21633834 | N_Shore | TSS1500 |  | PAX1 | I | PAX1 (-462) |
| cg25333216 | 0.071 | 0.056 | 0.015 | 0.00023 | Chr17:72245033 | Island | TSS1500;TSS200 |  | MIR636;SFRS2;MFSD11 | I | MFSD11 (-144), SRSF2 (+54) |
| cg11913790 | 0.194 | 0.165 | 0.029 | 0.00024 | Chr5:154116329 | Island | Body |  | LARP1 | II | LARP1 (+43675), C5orf4 (+94076) |
| cg26169156 | 0.521 | 0.566 | -0.045 | 0.00025 | Chr8:128909061 |  | Body |  | PVT1 | II | MYC (+91565) |
| cg14297332 | 0.893 | 0.876 | 0.017 | 0.00025 | Chr2:241828117 | Island | Body |  | HDLBP | II | HDLBP (+32801), ANO7 (+51521) |
| cg00155429 | 0.066 | 0.050 | 0.016 | 0.00026 | Chr1:152422321 | Island | 1stExon;5'UTR;Body |  | TPM3 | I | NUP210L (-28106), TPM3 (+8911) |
| cg10673740 | 0.138 | 0.154 | -0.016 | 0.00027 | Chr10:121401816 | Island | Body |  | BAG3 | II | BAG3 (+945) |
| cg14883916 | 0.098 | 0.129 | -0.031 | 0.00028 | Chr11:6904117 | Island | TSS200 |  | ZNF215 | I | ZNF215 (-112) |
| cg11214889 | 0.043 | 0.066 | -0.023 | 0.00028 | Chr18:587111 |  | 5'UTR;1stExon |  | CLUL1 | II | CLUL1 (-19588), CETN1 (+16743) |
| cg05617732 | 0.918 | 0.896 | 0.023 | 0.00029 | Chr6:47361869 |  | Body |  | TNFRSF21 | II | GPR110 (-243829), TNFRSF21 (+23769) |
| cg06740963 | 0.050 | 0.055 | -0.005 | 0.00030 | Chr16:2767349 | Island | TSS200 |  | TCEB2 | I | TCEB2 (-52) |
| cg07154861 | 0.034 | 0.039 | -0.005 | 0.00030 | Chr2:121210284 | Island |  |  |  | I | GLI2 (-61052), INHBB (+390096) |
| cg11550971 | 0.317 | 0.345 | -0.028 | 0.00030 | Chr18:11971317 | Island | TSS200 |  | IMPA2 | I | IMPA2 (-109) |
| cg09691810 | 0.052 | 0.060 | -0.008 | 0.00030 | Chr5:34951393 | Island | 1stExon;Body;TSS200;5'UTR |  | RAD1;;BRIX1 | II | BRIX1 (-183), RAD1 (+143) |
| cg01925594 | 0.870 | 0.833 | 0.037 | 0.00030 | Chr8:1308193 | N_Shore |  |  |  | II | C8orf42 (-825456), DLGAP2 (-128782) |
| cg22690046 | 0.030 | 0.026 | 0.003 | 0.00030 | Chr1:36636250 | S_Shore | TSS200 | rs60673492 | LSM10 | I | LSM10 (-171) |
| cg10409248 | 0.049 | 0.042 | 0.007 | 0.00032 | Chr17:75389668 | Island |  |  |  | I | CBX8 (-4159) |
| cg02434121 | 0.562 | 0.492 | 0.070 | 0.00032 | Chr7:4868324 | S_Shore | TSS200;Body |  | PAPOLB;RADIL | I | PAPOLB (-174) |
| cg23016766 | 0.030 | 0.034 | -0.004 | 0.00032 | Chr1:32889284 | Island | TSS200;TSS1500 |  | RBBP4;ZBTB8OS | I | RBBP4 (-51) |
| cg24419324 | 0.136 | 0.109 | 0.027 | 0.00033 | Chr2:222880573 | S_Shore |  |  |  | I | SGPP2 (-116992), PAX3 (-8615) |
| cg16347279 | 0.091 | 0.120 | -0.029 | 0.00033 | Chr17:70179304 | Island | 5'UTR;1stExon |  | RAB37 | II | CD300E (-47813), CD300LF (+41398) |
| cg26616283 | 0.071 | 0.062 | 0.008 | 0.00033 | Chr7:11838060 |  | 1stExon |  | THSD7A | I | THSD7A (+288) |
| cg19706795 | 0.379 | 0.316 | 0.063 | 0.00033 | Chr7:24764364 | S_Shore | TSS1500 |  | DFNA5 | II | DFNA5 (-757) |
| cg05716350 | 0.885 | 0.857 | 0.028 | 0.00034 | Chr4:79811906 |  |  |  |  | II | BMP2K (-104649), ANXA3 (+120141) |
| cg10092691 | 0.884 | 0.909 | -0.025 | 0.00034 | Chr1:203658419 |  | TSS1500 |  | LEMD1 | II | LEMD1 (-583) |
| cg10307119 | 0.875 | 0.854 | 0.021 | 0.00035 | Chr10:103979244 | N_Shore | 3'UTR |  | ELOVL3 | II | ELOVL3 (+3112), PITX3 (+11976) |
| cg10590925 | 0.401 | 0.467 | -0.066 | 0.00035 | Chr11:1420196 | N_Shore | Body |  | BRSK2 | II | MOB2 (+38494), BRSK2 (+52492) |
| cg16706629 | 0.079 | 0.089 | -0.010 | 0.00035 | Chr5:93980117 | Island | TSS200 |  | ANKRD32;C5orf36 | II | ANKRD32 (-29) |
| cg22641058 | 0.872 | 0.839 | 0.033 | 0.00037 | Chr2:130867352 | S_Shore |  |  |  | II | CFC1 (-127784), PTPN18 (+37303) |
| cg21907579 | 0.122 | 0.074 | 0.048 | 0.00038 | Chr12:113330251 | Island | 1stExon;5'UTR |  | TBX5 | II | TBX5 (-1980) |
| cg16123090 | 0.246 | 0.196 | 0.050 | 0.00038 | Chr2:3677085 | Island |  |  |  | II | ALLC (-6575), COLEC11 (+56574) |
| cg02335306 | 0.901 | 0.920 | -0.019 | 0.00039 | Chr11:2516456 |  | Body |  | KCNQ1 | I | KCNQ1 (+93660), CDKN1C (+347114) |
| cg01725864 | 0.897 | 0.881 | 0.016 | 0.00039 | Chr13:97627558 | S_Shore | 5'UTR;TSS200;5'UTR |  | FARP1;RNF113B | II | RNF113B (-37) |
| cg10593713 | 0.720 | 0.658 | 0.062 | 0.00039 | Chr10:3779788 |  |  |  |  | II | PITRM1 (-574756), KLF6 (+37684) |
| cg05869173 | 0.052 | 0.058 | -0.005 | 0.00039 | Chr7:99451128 | Island | TSS200 |  | ZKSCAN1 | I | ZKSCAN1 (-26) |
| cg24459409 | 0.338 | 0.298 | 0.040 | 0.00040 | Chr19:51196252 | S_Shore | Body |  | CCDC61 | II | CCDC61 (+6074), PGLYRP1 (+22143) |
| cg02002960 | 0.894 | 0.872 | 0.022 | 0.00041 | Chr19:5406386 | N_Shore | TSS200 | rs17271813 | ZNRF4 | II | ZNRF4 (-39) |
| cg25867726 | 0.042 | 0.049 | -0.007 | 0.00041 | Chr19:10057726 | Island | TSS200 |  | C19orf66 | I | ANGPTL6 (+16698), RDH8 (+72802) |
| cg21158737 | 0.769 | 0.808 | -0.040 | 0.00042 | Chr9:139463438 | Island | 3'UTR |  | NELF | I | ENTPD8 (-7717), NELF (+10168) |
| cg03184243 | 0.168 | 0.131 | 0.037 | 0.00042 | Chr8:28403881 | N_Shelf | TSS200 |  | FBXO16 | II | FZD3 (-3759) |
| cg17516330 | 0.632 | 0.675 | -0.043 | 0.00042 | Chr15:72281834 |  | 5'UTR |  | STRA6 | II | STRA6 (+438) |
| cg04064828 | 0.862 | 0.902 | -0.040 | 0.00042 | Chr10:133852741 | S_Shore | Body |  | DPYSL4 | I | DPYSL4 (+2338), STK32C (+118725) |
| cg20416037 | 0.083 | 0.111 | -0.028 | 0.00042 | Chr12:56270979 | N_Shore | TSS1500 |  | PIP4K2C | II | PIP4K2C (-229) |
| cg07321536 | 0.419 | 0.373 | 0.046 | 0.00042 | Chr4:39136292 | N_Shore | TSS1500;Body |  | LIAS;RPL9 | II | LIAS (-767), RPL9 (+309) |
| cg11985680 | 0.074 | 0.054 | 0.020 | 0.00042 | Chr1:76024455 |  | TSS1500;TSS200 |  | SNORD45C;RABGGTB | II | RABGGTB (-18) |
| cg24025255 | 0.298 | 0.354 | -0.056 | 0.00043 | Chr14:68687527 | N_Shore | Body |  | DCAF5 | II | ACTN1 (-171692), DCAF5 (+2139) |
| cg21963436 | 0.115 | 0.100 | 0.015 | 0.00044 | Chr13:27439142 | Island | Body |  | CDX2 | I | CDX2 (+2174), PDX1 (+46975) |
| cg11021661 | 0.847 | 0.872 | -0.026 | 0.00044 | Chr11:76430237 | S_Shore | 3'UTR |  | B3GNT6 | II | CAPN5 (-25402), B3GNT6 (+7155) |
| cg03441257 | 0.075 | 0.064 | 0.010 | 0.00044 | Chr1:148305713 | N_Shore | TSS1500 |  | VPS45 | I | VPS45 (-252) |
| cg21879791 | 0.451 | 0.493 | -0.042 | 0.00044 | Chr6:29702809 | N_Shore | Body |  | GABBR1 | II | GABBR1 (+6131), OR2H2 (+39148) |
| cg04958124 | 0.075 | 0.087 | -0.013 | 0.00045 | Chr7:148454795 | Island | 5'UTR;TSS1500 |  | ZNF398;ZNF425 | I | ZNF425 (-425) |
| cg02335804 | 0.085 | 0.113 | -0.028 | 0.00045 | Chr17:33179352 | N_Shore | TSS200 |  | HNF1B | II | HNF1B (-144) |
| cg16442298 | 0.809 | 0.767 | 0.042 | 0.00045 | Chr8:1390457 | N_Shore |  |  |  | II | C8orf42 (-907720), DLGAP2 (-46518) |
| cg25810178 | 0.508 | 0.547 | -0.039 | 0.00046 | Chr19:60569183 | N_Shelf | 3'UTR |  | IL11 | II | COX6B2 (-10909), IL11 (+4442) |
| cg18516946 | 0.890 | 0.860 | 0.030 | 0.00046 | Chr11:94774414 | S_Shelf |  |  |  | II | SRSF8 (-25641), KDM4DL (+15993) |
| cg23208152 | 0.037 | 0.030 | 0.007 | 0.00047 | Chr3:50383134 | Island | 1stExon;5'UTR |  | ZMYND10 | I | RASSF1 (-4768), ZMYND10 (+21) |
| cg27603283 | 0.101 | 0.094 | 0.007 | 0.00047 | Chr7:4998649 | Island | 1stExon;5'UTR |  | MMD2 | I | MMD2 (+194) |
| cg26841013 | 0.281 | 0.234 | 0.048 | 0.00047 | Chr1:228248013 | S_Shore | 3'UTR |  | WNT3A | I | ARF1 (-22347), WNT3A (+53291) |
| cg23327070 | 0.134 | 0.147 | -0.013 | 0.00047 | Chr14:93214896 |  | 1stExon;5'UTR |  | LGMN | I | LGMN (+150) |
| cg23842941 | 0.665 | 0.694 | -0.028 | 0.00048 | Chr20:33762631 | Island | Body |  | PROCR | I | MMP24 (-51907), PROCR (+2858) |
| cg10728960 | 0.904 | 0.889 | 0.015 | 0.00048 | Chr3:78079111 |  |  |  |  | I | ROBO2 (+989818) |
| cg17993148 | 0.894 | 0.914 | -0.020 | 0.00048 | Chr6:169717551 |  |  |  |  | II | C6orf120 (-384705), THBS2 (-63415) |
| cg21937169 | 0.129 | 0.101 | 0.029 | 0.00049 | Chr7:65541137 | S_Shore | Body |  | ASL | II | ASL (+362) |
| cg15166583 | 0.652 | 0.708 | -0.056 | 0.00049 | Chr11:86208322 |  | Body |  | ME3 | II | ME3 (+174917), C11orf73 (+195070) |
| cg23715749 | 0.464 | 0.419 | -0.045 | 0.00050 | Chr1:37413867 |  | Body |  | GRIK3 | II | CSF3R (-464953), GRIK3 (+85976) |
| cg18170076 | 0.609 | 0.562 | -0.047 | 0.00050 | Chr7:76240583 |  | 3'UTR;Body;Body |  | POMZP3;LOC100133091 | II | POMZP3 (+16036), UPK3B (+100839) |
| cg09571345 | 0.673 | 0.711 | 0.037 | 0.00050 | Chr10:26931926 |  | TSS200 |  | LOC731789 | I | PDSS1 (-54668), APBB1IP (+204661) |
| cg07059157 | 0.667 | 0.697 | 0.030 | 0.00051 | Chr12:54519802 | Island | TSS200 |  | LOC400043 | I | SMUG1 (+62975), HOXC4 (+72142) |
| cg25140501 | 0.058 | 0.074 | 0.016 | 0.00051 | Chr14:23564641 | Island | 5'UTR;1stExon;TSS200 |  | ACIN1;C14orf119 | II | ACIN1 (+181) |
| cg22220310 | 0.959 | 0.967 | 0.008 | 0.00051 | Chr1:1168541 | Island | TSS1500;1stExon |  | SDF4;B3GALT6 | I | SDF4 (-1095), B3GALT6 (+913) |
| cg13279019 | 0.962 | 0.947 | -0.015 | 0.00051 | Chr7:5266987 | Island | Body |  | WIPI2 | I | SLC29A4 (-55573), WIPI2 (+37153) |
| cg10455412 | 0.920 | 0.899 | -0.021 | 0.00053 | Chr12:67919042 |  |  |  |  | II | DYRK2 (-123469), CAND1 (+255982) |
| cg01751341 | 0.057 | 0.048 | -0.009 | 0.00053 | Chr16:85045118 | Island | 5'UTR;1stExon;5'UTR;1stExon |  | ZDHHC7 | II | ZDHHC7 (+22) |
| cg27380218 | 0.072 | 0.088 | 0.016 | 0.00053 | Chr15:99602811 | Island |  |  |  | I | PGPEP1L (-53927), SYNM (-42474) |
| cg10113589 | 0.756 | 0.710 | -0.046 | 0.00053 | Chr10:98118853 |  | 1stExon;5'UTR |  | OPALIN | II | OPALIN (+238) |
| cg00441301 | 0.815 | 0.845 | 0.031 | 0.00056 | Chr8:33458593 | S_Shore | TSS1500 |  | DUSP26 | II | DUSP26 (-1155) |
| cg22468497 | 0.651 | 0.618 | -0.033 | 0.00056 | Chr19:54241421 |  | TSS1500 |  | MIR518A2 | II | NLRP12 (+86226), DPRX (+106112) |
| cg22081003 | 0.734 | 0.773 | 0.039 | 0.00056 | Chr1:55416790 |  |  |  |  | II | DHCR24 (-63870), TMEM61 (-29674) |
| cg10556999 | 0.831 | 0.866 | 0.036 | 0.00057 | Chr1:158088745 |  |  |  |  | II | CD1D (-60991), KIRREL (+125683) |
| cg02977752 | 0.086 | 0.100 | 0.014 | 0.00058 | Chr7:86689099 | Island | TSS200 |  | KIAA1324L | I | KIAA1324L (-86) |
| cg05987564 | 0.878 | 0.901 | 0.023 | 0.00059 | Chr2:156981620 |  |  |  |  | II | NR4A2 (+207666) |
| cg12746717 | 0.093 | 0.108 | 0.015 | 0.00059 | Chr2:240323484 | Island | TSS1500 |  | HDAC4 | I | HDAC4 (-842) |
| cg04499015 | 0.580 | 0.534 | -0.046 | 0.00059 | Chr6:33141696 |  | Body |  | COL11A2 | II | COL11A2 (+18548), HLA-DPB1 (+97994) |
| cg07234439 | 0.859 | 0.880 | 0.021 | 0.00059 | Chr9:110479374 | Island |  |  |  | I | KLF4 (-227328) |
| cg17830308 | 0.133 | 0.181 | 0.049 | 0.00060 | Chr5:134872124 | S_Shore | TSS1500 |  | NEUROG1 | II | NEUROG1 (-486) |
| cg11050988 | 0.713 | 0.763 | 0.050 | 0.00060 | Chr7:1952600 | S_Shelf | Body |  | MAD1L1 | I | ELFN1 (+203803), MAD1L1 (+319982) |
| cg00627347 | 0.878 | 0.825 | -0.053 | 0.00060 | Chr17:915265 | N_Shore | Body |  | ABR | I | TIMM22 (+14909), ABR (+167865) |
| cg07145229 | 0.938 | 0.951 | 0.013 | 0.00062 | Chr8:99422528 |  |  |  |  | I | NIPAL2 (-115908), KCNS2 (-16721) |
| cg07086918 | 0.118 | 0.145 | 0.027 | 0.00062 | Chr5:176784688 |  | TSS200 |  | RGS14 | II | RGS14 (-155) |
| cg10929213 | 0.070 | 0.064 | -0.006 | 0.00062 | Chr11:66056787 | Island | TSS200 |  | YIF1A | I | TMEM151A (-2585), YIF1A (-150) |
| cg14791747 | 0.233 | 0.255 | 0.022 | 0.00062 | Chr16:20752902 | Island | 1stExon |  | THUMPD1 | II | ACSM1 (-50325), ACSM3 (-22409) |
| cg17196805 | 0.887 | 0.866 | -0.020 | 0.00063 | Chr17:37796442 | S_Shelf | 5'UTR |  | STARD3 | II | TCAP (-25156), STARD3 (+3110) |
| cg21045000 | 0.071 | 0.093 | 0.022 | 0.00063 | Chr12:118811074 | S_Shore | TSS1500 |  | TAOK3 | II | SUDS3 (-3283), TAOK3 (-325) |
| cg12363545 | 0.049 | 0.059 | 0.010 | 0.00063 | Chr3:107809973 | Island | TSS200 |  | CD47 | I | CD47 (-39) |
| cg27162392 | 0.765 | 0.708 | -0.057 | 0.00063 | Chr7:2208908 |  | Body |  | MAD1L1 | II | MAD1L1 (+63674), ELFN1 (+460111) |
| cg15847198 | 0.059 | 0.071 | 0.012 | 0.00064 | Chr11:106889318 | Island | TSS200 |  | GUCY1A2 | I | GUCY1A2 (-148) |
| cg22980293 | 0.635 | 0.592 | -0.043 | 0.00064 | Chr10:126158010 |  | Body |  | LHPP | II | LHPP (+7670), FAM53B (+274919) |
| cg18730023 | 0.154 | 0.201 | 0.047 | 0.00064 | Chr12:44199730 | Island | Body |  | TWF1 | II | TWF1 (+447) |
| cg20562220 | 0.933 | 0.918 | -0.015 | 0.00065 | Chr14:106068824 | S_Shore |  |  |  | I | TMEM121 (+75872), IGHE (+260637) |
| cg08917121 | 0.746 | 0.710 | -0.037 | 0.00066 | Chr5:176097773 |  |  |  |  | II | UNC5A (-139786), TSPAN17 (+23386) |
| cg16539675 | 0.084 | 0.104 | 0.019 | 0.00066 | Chr5:31532179 | Island | 5'UTR;1stExon;TSS200 |  | RNASEN;C5orf22 | I | DROSHA (+102) |
| cg06890152 | 0.862 | 0.887 | 0.025 | 0.00066 | Chr8:19800242 | S_Shelf | Body |  | LPL | II | LPL (+3661), SLC18A1 (+240474) |
| cg06293099 | 0.639 | 0.620 | -0.019 | 0.00067 | Chr11:67745999 |  |  |  |  | II | ALDH3B2 (-297315), UNC93B1 (+25593) |
| cg19984355 | 0.837 | 0.871 | 0.033 | 0.00067 | Chr5:1794232 |  |  |  |  | II | LPCAT1 (-270157), MRPL36 (+5723) |
| cg22827324 | 0.791 | 0.813 | 0.022 | 0.00067 | Chr2:33612876 |  | Body |  | LTBP1 | II | RASGRP3 (-126065), LTBP1 (+440508) |
| cg18766170 | 0.059 | 0.051 | -0.008 | 0.00068 | Chr6:30421655 | S_Shelf |  |  |  | I | HLA-E (-35527), TRIM39-RPP21 (+124568) |
| cg02824202 | 0.049 | 0.044 | -0.006 | 0.00068 | Chr18:6729810 | Island |  |  |  | I | L3MBTL4 (-314901), ARHGAP28 (-104621) |
| cg10583297 | 0.066 | 0.075 | 0.008 | 0.00069 | Chr15:45422095 | Island | TSS200 |  | DUOX1 | I | DUOX1 (-96), DUOXA1 (-39) |
| cg25719439 | 0.041 | 0.038 | -0.003 | 0.00070 | Chr16:89788035 | Island | TSS1500;5'UTR;1stExon |  | C16orf7;ZNF276 | I | C16orf7 (-642), ZNF276 (+84) |
| cg16073236 | 0.786 | 0.821 | 0.035 | 0.00070 | Chr7:107789943 |  | 3'UTR |  | NRCAM | II | LAMB4 (-19143), NRCAM (+90670) |
| cg02794434 | 0.582 | 0.522 | -0.060 | 0.00071 | Chr4:2341196 | Island | Body | rs2071680 | ZFYVE28 | II | MXD4 (-77458), ZFYVE28 (+79173) |
| cg13519194 | 0.734 | 0.704 | -0.030 | 0.00073 | Chr10:43539572 |  |  |  |  | I | RET (-32944), BMS1 (+261619) |
| cg11609366 | 0.060 | 0.083 | 0.023 | 0.00074 | Chr8:146018277 | Island | TSS1500 |  | RPL8 | II | RPL8 (-473) |
| cg19919692 | 0.121 | 0.147 | 0.027 | 0.00074 | Chr13:32421462 | S_Shore | Body |  | EEF1DP3 | II | FRY (-183974), RXFP2 (+107784) |
| cg16041550 | 0.788 | 0.819 | 0.031 | 0.00075 | Chr19:47633739 | N_Shore | TSS1500 |  | SAE1 | II | SAE1 (-340) |
| cg09901342 | 0.863 | 0.883 | 0.021 | 0.00075 | Chr13:64418337 | N_Shore |  |  |  | I | NONE |
| cg00844950 | 0.122 | 0.086 | -0.036 | 0.00075 | Chr19:1789060 | N_Shore | Body |  | ATP8B3 | II | ATP8B3 (+23138), ONECUT3 (+35399) |
| cg09172752 | 0.069 | 0.084 | 0.016 | 0.00076 | Chr15:77197536 | Island |  |  |  | I | RCN2 (-26425), SCAPER (-21320) |
| cg21294616 | 0.696 | 0.615 | -0.082 | 0.00076 | Chr7:143093848 |  | Body |  | EPHA1 | II | EPHA1 (+12136), ZYX (+15489) |
| cg18959169 | 0.885 | 0.855 | -0.030 | 0.00077 | Chr2:240239988 |  | Body |  | HDAC4 | II | HDAC4 (+82654), TWIST2 (+483316) |
| cg17780413 | 0.873 | 0.851 | -0.022 | 0.00077 | Chr17:703938 |  | 3'UTR |  | NXN | II | GLOD4 (-18368), NXN (+179059) |
| cg24974729 | 0.783 | 0.698 | -0.085 | 0.00077 | Chr11:67764320 | N_Shore | Body |  | UNC93B1 | I | ALDH3B2 (-315636), UNC93B1 (+7272) |
| cg11652597 | 0.831 | 0.861 | 0.030 | 0.00079 | Chr20:48521725 | N_Shore | 3'UTR |  | SPATA2 | II | SPATA2 (+10354), SLC9A8 (+92476) |
| cg23530850 | 0.078 | 0.064 | -0.014 | 0.00079 | Chr16:85647010 | Island | 5'UTR;Body |  | KIAA0182 | II | KIAA0182 (+87) |
| cg18976765 | 0.053 | 0.042 | -0.012 | 0.00079 | Chr13:101240690 | Island |  |  |  | II | A2LD1 (-54635), TMTC4 (+86412) |
| cg02286091 | 0.091 | 0.070 | -0.021 | 0.00079 | Chr5:78407678 |  | 5'UTR;1stExon |  | BHMT | I | BHMT (+75) |
| cg11534574 | 0.857 | 0.825 | -0.031 | 0.00079 | Chr22:41739529 | N_Shelf | Body |  | ZC3H7B | II | TEF (-38433), ZC3H7B (+41963) |
| cg19807694 | 0.863 | 0.888 | 0.025 | 0.00080 | Chr1:223835369 |  | Body |  | CAPN8 | II | SUSD4 (-297826), CAPN8 (+18066) |
| cg06353069 | 0.729 | 0.789 | 0.060 | 0.00081 | Chr2:238500081 |  | TSS1500 |  | RAB17 | II | RAB17 (-313) |
| cg09778229 | 0.162 | 0.145 | -0.017 | 0.00082 | Chr10:48354530 | Island | TSS1500 |  | ZNF488 | I | ZNF488 (-558) |
| cg14150280 | 0.074 | 0.088 | 0.014 | 0.00083 | Chr10:70660547 | Island | TSS1500 |  | DDX50 | I | DDX50 (-486) |
| cg11165357 | 0.095 | 0.108 | 0.013 | 0.00083 | Chr1:38274375 | Island | Body;TSS1500 |  | C1orf122;YRDC | II | YRDC (-511) |
| cg10140728 | 0.321 | 0.272 | -0.049 | 0.00083 | Chr15:63569646 | Island | TSS200 |  | APH1B | I | APH1B (-102) |
| cg14483771 | 0.821 | 0.866 | 0.046 | 0.00083 | Chr10:134998463 | N_Shore | Body |  | KNDC1 | II | UTF1 (-45314), KNDC1 (+24493) |
| cg22846582 | 0.775 | 0.812 | 0.037 | 0.00084 | Chr12:52848214 |  |  |  |  | II | KRT6B (-2305) |
| cg21514529 | 0.544 | 0.484 | -0.060 | 0.00084 | Chr5:168885285 |  |  |  |  | II | SLIT3 (-157153), CCDC99 (-125352) |
| cg20408147 | 0.089 | 0.111 | 0.023 | 0.00085 | Chr1:239551249 | Island |  |  |  | II | CHRM3 (-241123) |
| cg11828180 | 0.282 | 0.340 | 0.058 | 0.00085 | Chr1:41848985 | Island |  |  |  | I | SCMH1 (-141171), EDN2 (+101358) |
| cg18878150 | 0.564 | 0.503 | -0.060 | 0.00085 | Chr19:11594384 | Island | 3'UTR |  | ZNF653 | II | ELAVL3 (-2582) |
| cg19607983 | 0.077 | 0.071 | -0.006 | 0.00085 | Chr4:39528789 | Island | 5'UTR |  | UGDH | I | UGDH (+428) |
| cg21417399 | 0.068 | 0.075 | 0.008 | 0.00085 | Chr8:145598329 | Island | Body |  | ADCK5 | I | ADCK5 (+599) |
| cg01760475 | 0.067 | 0.079 | 0.012 | 0.00085 | Chr8:145104359 | Island |  |  |  | I | OPLAH (+11224), SPATC1 (+17778) |
| cg12585158 | 0.098 | 0.075 | -0.023 | 0.00086 | Chr3:121265726 | S_Shore | TSS1500 |  | POLQ | II | POLQ (-874) |
| cg05479657 | 0.773 | 0.817 | 0.044 | 0.00088 | Chr12:129790332 | S_Shelf | Body |  | TMEM132D | II | GLT1D1 (+452252), TMEM132D (+597879) |
| cg26790372 | 0.102 | 0.126 | 0.023 | 0.00089 | Chr18:5542879 | Island | 5'UTR |  | EPB41L3 | II | ZFP161 (-246686), EPB41L3 (+1106) |
| cg14287512 | 0.111 | 0.092 | -0.018 | 0.00090 | Chr1:228074806 | N_Shore |  |  |  | II | WNT9A (+60869), PRSS38 (+71389) |
| cg08080038 | 0.845 | 0.815 | -0.030 | 0.00090 | Chr3:61655044 |  | Body |  | PTPRG | II | PTPRG (+107802), FEZF2 (+704145) |
| cg14716734 | 0.725 | 0.691 | -0.035 | 0.00091 | Chr10:18549397 |  | TSS200;Body |  | CACNB2 | II | CACNB2 (+119792), NSUN6 (+391152) |
| cg07750914 | 0.106 | 0.093 | -0.013 | 0.00091 | Chr2:60983838 | Island | Body |  | PAPOLG | I | PAPOLG (+474) |
| cg15797527 | 0.543 | 0.494 | -0.049 | 0.00091 | Chr6:135814781 | N_Shelf | 5'UTR |  | AHI1 | II | AHI1 (+4121), MYB (+312329) |
| cg09197112 | 0.220 | 0.252 | 0.032 | 0.00091 | Chr10:46992448 | Island | TSS1500 |  | GPRIN2 | II | PPYR1 (-91085), SYT15 (-21848) |
| cg21894762 | 0.868 | 0.898 | 0.029 | 0.00092 | Chr14:73731131 |  | Body |  | PAPLN | II | PAPLN (+26927), NUMB (+194154) |
| cg02524205 | 0.476 | 0.539 | 0.063 | 0.00092 | Chr6:167559851 |  |  |  |  | I | GPR31 (+11467), CCR6 (+34557) |
| cg03801179 | 0.046 | 0.056 | 0.009 | 0.00093 | Chr2:20212423 | Island | 5'UTR;1stExon |  | MATN3 | I | MATN3 (+31) |

*Note.* Ranked by *p* value (p < 0.001). ∆β, difference in DNA methylation; DMPs, differentially methylated positions; GREAT, Genomic Regions Enrichment of Annotations Tool; Hg19, Human Genome build 19; SBE, single-base extension; SNP, single nucleotide polymorphism; TSS, transcription start site.

**Supplementary Table 6.** The top 100 ranked CpG sites which show changes in DNA methylation levels between age 5 and age 10 in the monozygotic twins discordant for age-12 psychotic symptoms

| **Probe ID** | **Affected Twin Mean** | **Co-Twin Mean** | **Mean ∆β** | **P value** | **Relation to CpG Island** | **Gene region feature category (UCSC)** | **SNPs in probe (+/- 10bp SBE)** | **Hg19** | **Illumina Gene Annotation** | **Probe Type** | **Gene Annotation from GREAT (Distance from TSS)** |
| --- | --- | --- | --- | --- | --- | --- | --- | --- | --- | --- | --- |
| cg15797527 | 0.044 | -0.040 | 0.084 | 4.30E-06 | N_Shelf | 5'UTR |  | Chr6:135814781 | AHI1 | II | AHI1 (+4121), MYB (+312329) |
| cg10052038 | 0.040 | -0.036 | 0.076 | 7.91E-06 | N_Shore | 5'UTR |  | Chr3:196293939 | WDR53 | II | FBXO45 (-1785) |
| cg27403609 | -0.016 | 0.028 | -0.044 | 1.14E-05 | Island |  |  | Chr2:11101403 |  | I | PQLC3 (-194136), KCNF1 (+49341) |
| cg11556416 | -0.034 | 0.041 | -0.074 | 1.35E-05 | Island | TSS1500 |  | Chr2:191879252 | STAT1 | I | STAT1 (-277) |
| cg13567282 | 0.023 | -0.043 | 0.066 | 1.40E-05 |  | 5'UTR |  | Chr6:149653214 | MAP3K7IP2 | II | TAB2 (+13779), ZC3H12D (+152933) |
| cg19599395 | 0.066 | -0.007 | 0.073 | 1.40E-05 | N_Shore | Body | rs72972973 | Chr19:3837031 | ZFR2 | II | MATK (-50617), ZFR2 (+31995) |
| cg26998693 | -0.024 | 0.013 | -0.036 | 1.54E-05 | S_Shore | TSS1500 |  | Chr6:132834590 | STX7 | II | STX7 (-254) |
| cg19050351 | 0.037 | -0.052 | 0.089 | 1.76E-05 |  | Body |  | Chr2:113820090 | IL1F5 | II | IL1F10 (-5456), IL36RN (+3406) |
| cg18260625 | -0.011 | 0.015 | -0.027 | 1.87E-05 | Island |  |  | Chr5:42951192 |  | I | SEPP1 (-139169), C5orf39 (+89254) |
| cg14297966 | -0.003 | -0.057 | 0.055 | 2.40E-05 | N_Shore | Body |  | Chr9:35101708 | STOML2 | II | PIGO (-5163), STOML2 (+1445) |
| cg02524205 | -0.038 | 0.054 | -0.091 | 2.51E-05 |  |  |  | Chr6:167559851 |  | I | GPR31 (+11467), CCR6 (+34557) |
| cg12730562 | -0.025 | 0.041 | -0.067 | 2.87E-05 |  | 5'UTR;TSS200 |  | Chr11:44927876 | TSPAN18 | II | TP53I11 (+44731), TSPAN18 (+141901) |
| cg22827324 | -0.017 | 0.022 | -0.039 | 3.31E-05 |  | Body |  | Chr2:33612876 | LTBP1 | II | RASGRP3 (-126065), LTBP1 (+440508) |
| cg26613742 | 0.015 | -0.053 | 0.068 | 3.42E-05 | N_Shelf | TSS200;Body |  | Chr19:14225000 | PRKACA | II | SAMD1 (-23769), PRKACA (+3558) |
| cg03359468 | 0.014 | -0.008 | 0.023 | 3.62E-05 | Island | TSS200 |  | Chr8:26240463 | BNIP3L | I | BNIP3L (-59) |
| cg25367206 | 0.038 | -0.020 | 0.059 | 3.82E-05 | S_Shore | TSS1500 |  | Chr8:142239055 | SLC45A4 | I | SLC45A4 (-383) |
| cg03916630 | 0.053 | -0.040 | 0.092 | 4.01E-05 |  |  |  | Chr10:45065415 |  | II | TMEM72 (-341348), CXCL12 (-184871) |
| cg00496455 | 0.004 | -0.011 | 0.016 | 5.25E-05 | Island | TSS200;Body |  | Chr2:220118770 | TUBA4A | I | TUBA4A (-133), TUBA4B (+806) |
| cg03964515 | 0.014 | -0.027 | 0.041 | 5.27E-05 |  | Body |  | Chr8:21588527 | GFRA2 | I | GFRA2 (+57818) |
| cg08862148 | 0.001 | 0.029 | -0.028 | 5.68E-05 | Island | Body;1stExon;Body |  | Chr6:29595315 | GABBR1 | II | GABBR1 (+5646), OR2H2 (+39633) |
| cg10656871 | 0.031 | -0.027 | 0.058 | 5.84E-05 |  |  |  | Chr11:45765692 |  | I | CHST1 (-78487), SLC35C1 (-59930) |
| cg23417011 | -0.032 | 0.030 | -0.062 | 5.95E-05 |  | Body;TSS200 |  | Chr5:93077375 | FAM172A;POU5F2 | I | POU5F2 (-67) |
| cg06888554 | -0.007 | 0.009 | -0.015 | 6.01E-05 | Island | TSS200 |  | Chr19:42580220 | ZNF574 | I | ZNF574 (-69) |
| cg03464229 | 0.054 | -0.055 | 0.109 | 6.56E-05 |  | Body;Body |  | Chr7:120646878 | C7orf58 | II | WNT16 (-322211), C7orf58 (+18128) |
| cg26403608 | -0.045 | 0.084 | -0.129 | 6.75E-05 |  | TSS1500;3'UTR |  | Chr17:2319719 | LOC284009;METT10D | II | MNT (-15462), METTL16 (+95480) |
| cg08548315 | 0.044 | -0.032 | 0.076 | 6.89E-05 |  |  |  | Chr1:81609992 |  | II | LPHN2 (-656089) |
| cg08359347 | -0.011 | 0.004 | -0.015 | 7.11E-05 | Island | TSS1500 |  | Chr11:86667175 | FZD4 | I | FZD4 (-736) |
| cg22327778 | 0.051 | -0.014 | 0.066 | 7.23E-05 |  | TSS1500;TSS200 |  | Chr11:1860218 | TNNI2 | II | TNNI2 (-1213) |
| cg13839439 | 0.025 | -0.030 | 0.055 | 7.28E-05 | N_Shore |  |  | Chr12:54144521 |  | II | HOXC13 (-188054), CALCOCO1 (-23215) |
| cg08838610 | -0.020 | 0.006 | -0.026 | 7.31E-05 | Island | TSS200 |  | Chr17:5322907 | RPAIN | I | RPAIN (-53), NUP88 (+151) |
| cg01084257 | -0.028 | 0.021 | -0.049 | 7.89E-05 |  | Body;TSS1500 |  | Chr8:129020419 | PVT1;MIR1206 | II | MYC (+272105) |
| cg09696939 | -0.020 | 0.017 | -0.037 | 7.94E-05 | N_Shore | TSS1500 |  | Chr10:60272079 | BICC1 | I | BICC1 (-824) |
| cg21211020 | -0.014 | 0.010 | -0.024 | 8.02E-05 | Island | TSS200 |  | Chr8:86132685 | C8orf59 | II | CA13 (-25030), E2F5 (+43067) |
| cg06992486 | 0.094 | -0.027 | 0.120 | 8.05E-05 |  |  |  | Chr15:96855353 |  | II | NR2F2 (-18757) |
| cg00947686 | 0.031 | -0.017 | 0.048 | 8.16E-05 | S_Shore |  | rs35684285 | Chr1:5774259 |  | II | NPHP4 (+278273) |
| cg18500286 | -0.010 | 0.005 | -0.015 | 8.62E-05 | Island | TSS1500 |  | Chr4:87855942 | AFF1 | I | AFF1 (-211) |
| cg04274199 | 0.043 | -0.045 | 0.088 | 9.09E-05 | Island | Body |  | Chr21:47334057 | PCBP3 | I | COL6A1 (-67605), PCBP3 (+64183) |
| cg24153044 | 0.003 | -0.006 | 0.009 | 9.34E-05 | Island | 1stExon |  | Chr1:6485201 | ESPN | I | ESPN (+354) |
| cg24438749 | -0.022 | 0.014 | -0.036 | 9.35E-05 |  | Body |  | Chr13:110929101 | COL4A1 | II | IRS2 (-490188), COL4A1 (+30394) |
| cg24557917 | 0.014 | -0.031 | 0.046 | 0.0001013 |  | TSS200 |  | Chr4:165980112 | TRIM75 | II | TRIM60 (+26962), TMEM192 (+53911) |
| cg13800209 | -0.024 | 0.007 | -0.031 | 0.0001029 | Island | 1stExon;Body;5'UTR |  | Chr15:37390284 | MEIS2 | I | MEIS2 (+2456) |
| cg13858054 | 0.002 | -0.016 | 0.017 | 0.0001049 | Island | 5'UTR;TSS1500 |  | Chr9:86595264 | HNRNPK;RMI1 | I | RMI1 (-372), HNRNPK (-81) |
| cg01370541 | -0.032 | 0.015 | -0.047 | 0.0001069 | Island |  |  | Chr4:41882812 |  | II | PHOX2B (-131826), TMEM33 (-54324) |
| cg18247223 | -0.023 | 0.036 | -0.059 | 0.0001076 |  | 5'UTR |  | Chr11:44908673 | TSPAN18 | II | TP53I11 (+63934), TSPAN18 (+122698) |
| cg15425527 | 0.037 | -0.034 | 0.071 | 0.0001093 | N_Shelf |  |  | Chr19:47849437 |  | II | DHX34 (-3100) |
| cg27356115 | 0.051 | -0.013 | 0.064 | 0.000111 |  |  |  | Chr15:53422311 |  | II | UNC13C (-882789), ONECUT1 (-340103) |
| cg18837542 | 0.011 | -0.025 | 0.036 | 0.000113 |  |  |  | Chr4:139610152 |  | I | SLC7A11 (-446650), CCRN4L (-326790) |
| cg18014842 | 0.034 | -0.022 | 0.056 | 0.000115 |  | Body |  | Chr11:126355874 | KIRREL3 | II | ST3GAL4 (+130335), KIRREL3 (+514891) |
| cg06352924 | -0.025 | 0.029 | -0.055 | 0.0001185 | Island | Body |  | Chr3:150321728 | SELT | I | SELT (+663) |
| cg24912419 | -0.032 | 0.016 | -0.048 | 0.0001206 | Island | Body |  | Chr9:130922817 | C9orf16 | II | C9orf16 (+279) |
| cg08732623 | -0.018 | 0.009 | -0.027 | 0.0001229 | Island | 1stExon;5'UTR |  | Chr10:127408269 | C10orf137 | I | C10orf137 (+186) |
| cg07322688 | 0.009 | -0.003 | 0.012 | 0.0001274 | Island | Body |  | Chr7:122526033 | CADPS2 | I | CADPS2 (+779) |
| cg22380353 | -0.034 | 0.025 | -0.059 | 0.0001302 | S_Shelf |  |  | Chr13:42537904 |  | II | KIAA0564 (-2684) |
| cg26281728 | 0.001 | -0.010 | 0.011 | 0.0001321 | Island | 1stExon;5'UTR |  | Chr15:85923949 | AKAP13 | I | AKAP13 (+79) |
| cg23666536 | 0.036 | -0.047 | 0.083 | 0.0001348 |  | 3'UTR |  | Chr1:114240177 | PHTF1 | II | PHTF1 (+61599), MAGI3 (+306703) |
| cg13145644 | 0.053 | -0.050 | 0.103 | 0.0001353 |  | TSS1500 |  | Chr3:39324326 | CX3CR1 | II | CX3CR1 (-1101) |
| cg26327442 | -0.019 | 0.017 | -0.036 | 0.0001354 | S_Shelf |  |  | Chr11:82447767 |  | II | PRCP (+163789) |
| cg23079217 | -0.034 | 0.022 | -0.057 | 0.0001363 |  | TSS200;TSS1500 |  | Chr12:79257730 | SYT1 | I | SYT1 (-181702) |
| cg05134426 | 0.059 | -0.030 | 0.089 | 0.0001389 |  | 1stExon |  | Chr5:124080479 | ZNF608 | II | ZNF608 (+385) |
| cg24748771 | 0.021 | -0.017 | 0.039 | 0.0001516 |  | TSS1500 |  | Chr21:43300770 | PRDM15 | II | PRDM15 (-1180) |
| cg23395165 | 0.007 | -0.010 | 0.017 | 0.0001526 | Island | Body;Body |  | Chr19:4483111 | HDGF2 | I | HDGFRP2 (+10857), PLIN4 (+34604) |
| cg12715136 | 0.054 | -0.041 | 0.095 | 0.0001642 |  | 3'UTR |  | Chr10:100004513 | C10orf28 | II | LOXL4 (+23493), C10orf28 (+110133) |
| cg07144713 | 0.030 | -0.041 | 0.071 | 0.0001657 |  | 3'UTR |  | Chr16:85009145 | ZDHHC7 | II | ZDHHC7 (+35995), CRISPLD2 (+155559) |
| cg04064828 | 0.052 | -0.003 | 0.055 | 0.0001673 | S_Shore | Body |  | Chr10:134002751 | DPYSL4 | I | DPYSL4 (+2338), STK32C (+118725) |
| cg14827481 | 0.031 | -0.033 | 0.064 | 0.0001674 | S_Shore | Body | rs57835447 | Chr10:134361889 | INPP5A | II | INPP5A (+10537), NKX6-2 (+237647) |
| cg02925268 | 0.020 | -0.024 | 0.044 | 0.000172 |  |  |  | Chr12:132301999 |  | II | MMP17 (-10941), SFSWAP (+106365) |
| cg05462570 | -0.024 | 0.022 | -0.046 | 0.0001753 | N_Shore | Body |  | Chr11:64814948 | NAALADL1 | II | SAC3D1 (+6573), NAALADL1 (+11060) |
| cg09956924 | 0.024 | -0.036 | 0.060 | 0.0001771 |  | Body |  | Chr8:143596713 | BAI1 | I | BAI1 (+51337), ARC (+99119) |
| cg02770097 | 0.036 | -0.041 | 0.077 | 0.0001821 |  | Body |  | Chr10:88445317 | LDB3 | II | BMPR1A (-71078), LDB3 (+16892) |
| cg09786257 | -0.036 | 0.029 | -0.065 | 0.0001834 | N_Shore | 1stExon |  | Chr5:95768695 | PCSK1 | II | PCSK1 (+289) |
| cg02720566 | -0.032 | 0.015 | -0.047 | 0.0001839 | S_Shore | Body |  | Chr19:18230196 | MAST3 | II | PIK3R2 (-33819), MAST3 (+21594) |
| cg27162392 | 0.045 | -0.043 | 0.088 | 0.0001841 |  | Body |  | Chr7:2208908 | MAD1L1 | II | MAD1L1 (+63674), ELFN1 (+460111) |
| cg12920882 | -0.006 | 0.011 | -0.017 | 0.0001853 | Island | Body;5'UTR |  | Chr2:69664317 | NFU1 | II | NFU1 (+442) |
| cg21793452 | 0.041 | -0.034 | 0.075 | 0.0001861 | N_Shore |  |  | Chr3:197182658 |  | II | DLG1 (-157212), BDH1 (+100199) |
| cg26670245 | -0.005 | 0.003 | -0.008 | 0.0001892 | Island | 5'UTR;5'UTR;TSS1500 |  | Chr2:170551352 | PHOSPHO2;KLHL23;C2orf77 | I | PHOSPHO2 (+389) |
| cg06895675 | 0.026 | -0.034 | 0.060 | 0.0001907 | S_Shore |  |  | Chr10:88137553 |  | II | GRID1 (-11304), WAPAL (+143987) |
| cg27533472 | 0.023 | -0.010 | 0.033 | 0.0001963 |  | 1stExon;5'UTR |  | Chr11:59856225 | MS4A2 | II | MS4A2 (+89) |
| cg18333694 | 0.028 | -0.015 | 0.042 | 0.0001977 | S_Shore |  |  | Chr11:357882 |  | II | IFITM3 (-36969), B4GALNT4 (-11912) |
| cg23497569 | 0.054 | -0.062 | 0.115 | 0.0002004 |  |  |  | Chr7:19417585 |  | II | FERD3L (-232542), TWISTNB (+331074) |
| cg14750743 | 0.018 | -0.049 | 0.068 | 0.0002087 | S_Shelf | Body |  | Chr6:32137294 | AGPAT1 | II | EGFL8 (+4939), AGPAT1 (+6621) |
| cg15907392 | 0.029 | -0.024 | 0.053 | 0.0002104 | Island |  |  | Chr12:34494852 |  | II | ALG10 (+319637) |
| cg01917016 | -0.017 | 0.013 | -0.031 | 0.0002123 | Island | Body;5'UTR;1stExon |  | Chr1:206730504 | RASSF5 | II | RASSF5 (+49626), EIF2D (+55399) |
| cg24885794 | -0.026 | 0.062 | -0.088 | 0.0002176 | Island | TSS1500;5'UTR |  | Chr7:94286086 | SGCE;PEG10 | II | SGCE (-566), PEG10 (+405) |
| cg27425612 | 0.001 | -0.006 | 0.008 | 0.0002184 | Island | TSS200 |  | Chr1:201368843 | LAD1 | I | LAD1 (-175) |
| cg20856064 | -0.033 | 0.036 | -0.069 | 0.0002287 | Island | TSS200 |  | Chr3:49027210 | P4HTM | I | P4HTM (-130) |
| cg17025730 | -0.022 | 0.027 | -0.049 | 0.0002287 | N_Shore |  |  | Chr5:133859798 |  | I | PHF15 (-1999) |
| cg05790658 | 0.029 | -0.047 | 0.076 | 0.0002291 |  | 5'UTR |  | Chr4:143748032 | INPP4B | II | INPP4B (+19571) |
| cg24649269 | -0.020 | 0.021 | -0.041 | 0.0002316 | S_Shore | Body |  | Chr6:7231857 | RREB1 | II | SSR1 (+81683), RREB1 (+123772) |
| cg05294300 | 0.036 | -0.064 | 0.099 | 0.0002338 | Island | Body |  | Chr19:17317009 | MYO9B | II | USE1 (-9145), MYO9B (+130419) |
| cg27578734 | -0.026 | 0.051 | -0.076 | 0.0002343 | N_Shore |  |  | Chr12:132972609 |  | I | P2RX2 (-222793), GALNT9 (-66705) |
| cg12872560 | -0.024 | 0.011 | -0.035 | 0.0002382 | Island | Body |  | Chr10:50340047 | FAM170B | II | VSTM4 (-16489), C10orf128 (+56359) |
| cg15278386 | 0.017 | -0.046 | 0.063 | 0.0002385 | N_Shelf | 5'UTR |  | Chr20:61924802 | COL20A1 | I | COL20A1 (+265) |
| cg15111554 | 0.014 | -0.048 | 0.061 | 0.0002389 |  | Body |  | Chr1:110172028 | AMPD2 | II | GSTM4 (-26669), AMPD2 (+8753) |
| cg13667739 | -0.007 | 0.055 | -0.062 | 0.0002518 | Island | Body |  | Chr14:105944604 | CRIP2 | II | CRIP1 (-8652), CRIP2 (+3474) |
| cg24137511 | -0.009 | -0.081 | 0.072 | 0.0002519 | Island | Body |  | Chr19:18260330 | MAST3 | I | PIK3R2 (-3685) |
| cg05982757 | -0.053 | 0.030 | -0.083 | 0.0002602 | Island | 5'UTR;Body |  | Chr12:54427528 | HOXC4;HOXC5 | II | HOXC5 (+697) |
| cg07066163 | 0.048 | -0.026 | 0.074 | 0.0002602 |  |  |  | Chr16:85635689 |  | II | KIAA0182 (-11234), KIAA0513 (+574280) |
| cg13393580 | 0.020 | -0.030 | 0.049 | 0.0002615 | S_Shelf | TSS1500 |  | Chr1:1295077 | MXRA8 | I | MXRA8 (-1163) |
| cg17692200 | -0.003 | 0.009 | -0.012 | 0.0002639 | Island | TSS200 |  | Chr7:35840513 | SEPT7 | I | SEPT7 (-82) |
| cg23466291 | -0.016 | 0.011 | -0.027 | 0.0002656 | Island |  | rs12450085 | Chr17:75243612 |  | II | SEPT9 (-33879), SEC14L1 (+106608) |
| cg11251349 | -0.013 | 0.005 | -0.018 | 0.000267 | Island | TSS1500 |  | Chr1:33721607 | ZNF362 | I | ZNF362 (-566) |
| cg24811069 | 0.009 | -0.046 | 0.055 | 0.0002673 |  |  |  | Chr8:49049137 |  | II | UBE2V2 (+128143), EFCAB1 (+598732) |
| cg07142668 | -0.013 | 0.005 | -0.018 | 0.0002764 | Island | TSS1500 |  | Chr1:32230251 | BAI2 | II | BAI2 (-604) |
| cg13644282 | 0.019 | -0.039 | 0.058 | 0.0002781 | N_Shelf |  |  | Chr12:132616666 |  | I | DDX51 (+12213), EP400 (+182202) |
| cg20285609 | 0.023 | -0.010 | 0.033 | 0.0002782 |  | 5'UTR;Body |  | Chr1:41622030 | SCMH1 | II | SLFNL1 (-134604), SCMH1 (+85784) |
| cg26771582 | -0.025 | 0.026 | -0.051 | 0.0002784 | N_Shore | Body |  | Chr16:87415899 | FBXO31 | II | FBXO31 (+1494), FOXL1 (+803785) |
| cg02831037 | 0.060 | -0.037 | 0.098 | 0.0002833 | Island |  |  | Chr16:798278 |  | II | MSLN (-12486), NARFL (-7282) |
| cg18887483 | 0.021 | -0.013 | 0.034 | 0.0002851 | Island | 5'UTR;TSS1500;TSS200 |  | Chr15:74422572 | ISLR2;LOC283731 | II | ISLR2 (-170) |
| cg21548940 | 0.023 | -0.024 | 0.047 | 0.0002887 |  |  |  | Chr5:162921557 |  | II | MAT2B (-11027), HMMR (+34041) |
| cg10324825 | -0.020 | 0.015 | -0.035 | 0.0002911 | S_Shore | Body |  | Chr1:158153227 | CD1D | II | CD1A (-70699), CD1D (+3491) |
| cg26821579 | -0.047 | 0.009 | -0.056 | 0.0002949 | S_Shore | TSS200 | rs12802292 | Chr11:22851416 | SVIP | I | SVIP (-35) |
| cg17259741 | -0.008 | 0.006 | -0.014 | 0.0003002 | Island | 5'UTR |  | Chr4:4291994 | ZNF509;LYAR | I | LYAR (-99), ZBTB49 (+71) |
| cg24552773 | -0.018 | 0.033 | -0.051 | 0.0003015 | Island | Body |  | Chr1:9100506 | SLC2A5;SLC2A5 | II | SLC2A7 (-14103), SLC2A5 (+29380) |
| cg18126791 | -0.017 | 0.007 | -0.024 | 0.000302 | Island | 5'UTR;1stExon |  | Chr21:33031960 | SOD1 | I | SOD1 (+26) |
| cg26476156 | 0.038 | -0.066 | 0.104 | 0.0003043 | S_Shore | TSS1500 |  | Chr10:46090807 | MARCH8 | II | MARCH8 (-59989), ZFAND4 (+77443) |
| cg00423675 | 0.016 | -0.031 | 0.047 | 0.0003054 | N_Shore |  |  | Chr9:138067680 |  | II | PPP1R26 (-303967), OLFM1 (+100592) |
| cg04660577 | 0.044 | -0.043 | 0.087 | 0.000306 | S_Shore | TSS1500 |  | Chr4:103267999 | SLC39A8 | II | SLC39A8 (-1345) |
| cg13523014 | 0.032 | -0.081 | 0.114 | 0.0003061 | N_Shelf | Body |  | Chr19:8618073 | MYO1F | I | ZNF414 (-39026), MYO1F (+24257) |
| cg04217778 | 0.025 | -0.023 | 0.048 | 0.0003125 | Island | Body |  | Chr7:5111916 | LOC389458 | I | RBAK (+26364), ZNF890P (+60281) |
| cg01139526 | -0.015 | 0.008 | -0.022 | 0.0003181 |  | TSS200 |  | Chr5:139682850 | PFDN1 | II | PFDN1 (-162) |
| cg16711612 | -0.020 | -0.001 | -0.019 | 0.0003222 | Island | Body;TSS200 |  | Chr14:79745592 | NRXN3 | I | NRXN3 (+875500), DIO2 (+932377) |
| cg06770877 | 0.023 | -0.029 | 0.052 | 0.0003267 |  | Body |  | Chr13:31897247 | B3GALTL | II | RXFP2 (-416431), B3GALTL (+123136) |
| cg27180315 | 0.005 | -0.008 | 0.012 | 0.0003296 | Island | TSS200 |  | Chr7:75831194 | SRRM3 | I | HSPB1 (-100680), MDH2 (+153802) |
| cg06852824 | 0.033 | -0.030 | 0.063 | 0.0003305 | S_Shelf | 5'UTR |  | Chr2:231582109 | CAB39 | I | ITM2C (-147511), CAB39 (+4553) |
| cg09446567 | -0.024 | 0.016 | -0.040 | 0.0003328 |  |  |  | Chr4:179734204 |  | II | NONE |
| cg08441633 | 0.029 | -0.026 | 0.055 | 0.0003351 | Island | Body | rs7201334 | Chr16:88765295 | RNF166 | II | SNAI3 (-12414), RNF166 (+7533) |
| cg10856605 | -0.007 | 0.005 | -0.011 | 0.0003364 |  | 5'UTR | rs58290790 | Chr6:28048756 | ZNF165 | I | ZNF165 (+275) |
| cg03799283 | 0.069 | -0.016 | 0.085 | 0.0003407 | Island |  |  | Chr15:96910011 |  | I | NR2F2 (+35901) |
| cg20461912 | -0.016 | 0.032 | -0.048 | 0.000349 | Island | 1stExon;3'UTR |  | Chr3:138664350 | FOXL2 | II | PIK3CB (-186166), FOXL2 (+1631) |
| cg26564606 | 0.049 | -0.066 | 0.114 | 0.0003506 | N_Shelf | 3'UTR |  | Chr5:660180 | TPPP | II | TPPP (+33329), CEP72 (+47776) |
| cg00429402 | 0.014 | 0.000 | 0.014 | 0.0003592 | Island | 1stExon;5'UTR |  | Chr10:94833658 | CYP26A1 | I | CYP26A1 (+12) |
| cg20562220 | 0.009 | -0.019 | 0.028 | 0.0003625 | S_Shore |  |  | Chr14:106068824 |  | I | TMEM121 (+75872), IGHE (+260637) |
| cg24347422 | 0.023 | -0.024 | 0.048 | 0.0003629 | N_Shelf | Body | rs73955766 | Chr2:133400321 | GPR39 | II | LYPD1 (+28159), GPR39 (+226175) |
| cg11787218 | 0.024 | -0.053 | 0.077 | 0.0003717 |  | Body |  | Chr6:312105 | DUSP22 | I | IRF4 (-79633), DUSP22 (+20005) |
| cg15346359 | 0.074 | -0.005 | 0.080 | 0.0003778 |  | 3'UTR |  | Chr11:47606064 | NDUFS3 | II | FAM180B (-2165) |
| cg24163575 | 0.023 | -0.019 | 0.042 | 0.0003838 | Island |  |  | Chr15:30261379 |  | I | TJP1 (-146674), CHRFAM7A (+424484) |
| cg17074213 | -0.057 | 0.033 | -0.090 | 0.000385 | Island | 1stExon;5'UTR |  | Chr1:92351695 | TGFBR3 | I | TGFBR3 (+140) |
| cg20126647 | -0.019 | 0.018 | -0.037 | 0.0003888 | N_Shore | TSS200 |  | Chr17:40714003 | COASY | I | COASY (-88) |
| cg18342183 | 0.014 | -0.047 | 0.061 | 0.0003899 |  |  |  | Chr1:186732915 |  | II | PTGS2 (-83357), PLA2G4A (-65116) |
| cg21659346 | 0.025 | -0.007 | 0.032 | 0.000394 |  |  | rs57186292 | Chr11:72280409 |  | II | CLPB (-134842), PDE2A (+105087) |
| cg20655369 | 0.044 | -0.021 | 0.065 | 0.0003954 | S_Shelf | Body |  | Chr15:89924975 | LOC254559 | II | POLG (-46950), RHCG (+114823) |
| cg14462686 | 0.019 | -0.035 | 0.054 | 0.0003955 |  | Body |  | Chr9:4226519 | GLIS3 | II | RFX3 (-700537), GLIS3 (+73515) |
| cg25485913 | -0.051 | 0.034 | -0.085 | 0.0003992 |  |  |  | Chr19:49568962 |  | II | NTF4 (-1844) |
| cg09055822 | 0.029 | -0.033 | 0.063 | 0.0003993 | N_Shelf | Body |  | Chr8:142180375 | DENND3 | II | PTK2 (-168964), SLC45A4 (+58297) |
| cg15447829 | -0.044 | 0.022 | -0.067 | 0.0004004 | N_Shore | Body |  | Chr21:34960242 | DONSON | II | DONSON (+1024), SON (+44893) |
| cg20113500 | -0.023 | 0.014 | -0.037 | 0.0004004 | S_Shore | TSS200 |  | Chr12:113773013 | SLC24A6 | I | SLC24A6 (-89) |
| cg15445958 | -0.027 | 0.027 | -0.054 | 0.0004065 |  |  |  | Chr6:110266125 |  | II | GPR6 (-34172), FIG4 (+253702) |
| cg20064122 | -0.030 | 0.009 | -0.039 | 0.0004107 | Island | TSS200 |  | Chr22:18593362 | TUBA8 | II | TUBA8 (-90) |
| cg07202054 | -0.016 | 0.064 | -0.080 | 0.0004124 | S_Shelf |  |  | Chr1:48452740 |  | II | LOC388630 (+9821), FOXD2 (+551052) |
| cg20162822 | 0.002 | -0.016 | 0.018 | 0.0004141 | S_Shore | 3'UTR |  | Chr17:1658265 | SERPINF2 | I | SERPINF1 (-6993), SERPINF2 (+12136) |
| cg17926940 | -0.026 | 0.012 | -0.038 | 0.0004146 | Island |  |  | Chr14:97685060 |  | II | VRK1 (+421377) |
| cg12448989 | -0.033 | 0.005 | -0.038 | 0.0004167 | Island | 1stExon |  | Chr13:77459329 | KCTD12 | I | KCTD12 (+1210) |
| cg05002305 | 0.036 | -0.040 | 0.076 | 0.0004174 | Island | Body |  | Chr11:1273661 | MUC5B | II | MUC5B (+29367), TOLLIP (+57230) |
| cg18736676 | 0.025 | -0.057 | 0.082 | 0.0004182 |  |  |  | Chr6:109053447 |  | I | ARMC2 (-116171), FOXO3 (+171379) |
| cg02796790 | 0.015 | -0.072 | 0.086 | 0.0004212 | Island | Body |  | Chr19:10823761 | QTRT1 | II | DNM2 (-4967) |
| cg02210149 | 0.028 | -0.032 | 0.060 | 0.0004219 | N_Shore | TSS1500;Body |  | Chr6:32096599 | ATF6B;FKBPL | II | ATF6B (-583) |
| cg00932808 | 0.005 | -0.011 | 0.016 | 0.000422 | Island |  | rs11757362 | Chr6:14211322 |  | I | CD83 (+93458) |
| cg19059495 | 0.025 | -0.021 | 0.046 | 0.0004224 | Island |  |  | Chr6:30095495 |  | II | TRIM31 (-14629), TRIM40 (-9014) |
| cg17350432 | 0.032 | -0.014 | 0.045 | 0.0004259 | N_Shore |  |  | Chr4:841569 |  | II | CPLX1 (-21625), GAK (+84604) |
| cg17882580 | 0.017 | -0.014 | 0.030 | 0.0004261 |  | 3'UTR |  | Chr7:47315063 | TNS3 | II | TNS3 (+306678) |
| cg08141342 | -0.018 | 0.005 | -0.023 | 0.000428 | Island | TSS1500 |  | Chr1:45308976 | PTCH2 | I | PTCH2 (-361) |
| cg09706512 | -0.003 | 0.005 | -0.008 | 0.0004282 | Island | TSS1500 |  | Chr3:88108503 | CGGBP1 | I | CGGBP1 (-358) |
| cg24634810 | -0.012 | 0.004 | -0.016 | 0.0004288 | Island | TSS200;TSS200 |  | Chr17:40307043 | RAB5C | II | RAB5C (+18) |
| cg13299436 | 0.013 | -0.056 | 0.069 | 0.0004313 |  | TSS1500 |  | Chr6:29140622 | OR2J2 | II | OR14J1 (-133844), OR2B3 (-85533) |
| cg01149683 | 0.056 | -0.028 | 0.084 | 0.0004344 |  |  |  | Chr3:66705803 |  | II | LRIG1 (-154959), SUCLG2 (+999234) |
| cg07675811 | 0.035 | -0.028 | 0.063 | 0.0004352 | N_Shore | Body |  | Chr7:5426831 | TNRC18 | II | TNRC18 (+36345), SLC29A4 (+104271) |
| cg07713135 | -0.014 | 0.041 | -0.055 | 0.0004372 |  | Body |  | Chr13:99093756 | FARP1 | I | RNF113B (-264236), STK24 (+135639) |
| cg25635840 | 0.004 | -0.008 | 0.012 | 0.0004402 | N_Shore | Body |  | Chr17:26898051 | PIGS | II | PIGS (+835) |
| cg03255741 | 0.005 | -0.004 | 0.008 | 0.0004485 | Island | 1stExon;5'UTR |  | Chr18:48556964 | SMAD4 | I | SMAD4 (+382) |
| cg05241143 | -0.025 | 0.013 | -0.038 | 0.0004528 | N_Shelf | Body |  | Chr7:157916636 | PTPRN2 | II | PTPRN2 (+463845), DNAJB6 (+786927) |
| cg13091883 | 0.007 | -0.097 | 0.104 | 0.0004578 | S_Shelf | Body | rs76135941 | Chr1:111892655 | C1orf88 | II | C1orf88 (+3461), OVGP1 (+77743) |
| cg05754402 | 0.030 | -0.031 | 0.061 | 0.0004603 |  |  |  | Chr8:49063039 |  | II | UBE2V2 (+142045), EFCAB1 (+584830) |
| cg04416981 | -0.033 | 0.042 | -0.074 | 0.0004629 |  | TSS1500 |  | Chr16:89043707 | CBFA2T3 | II | CBFA2T3 (-204) |
| cg27314324 | 0.032 | -0.021 | 0.053 | 0.0004641 |  | Body |  | Chr1:112476724 | KCND3 | I | KCND3 (+55052), DDX20 (+178535) |
| cg07307994 | -0.032 | 0.008 | -0.040 | 0.0004828 |  |  | rs35938927 | Chr2:3828216 |  | II | ALLC (+122431) |
| cg14338234 | -0.019 | 0.010 | -0.029 | 0.0004853 | S_Shore | Body;TSS1500 |  | Chr3:93782253 | NSUN3;DHFRL1 | II | DHFRL1 (-187), NSUN3 (+399) |
| cg11214889 | 0.008 | -0.013 | 0.020 | 0.0004863 |  | 5'UTR;1stExon |  | Chr18:597111 | CLUL1 | II | CLUL1 (-19588), CETN1 (+16743) |
| cg08418841 | 0.002 | -0.003 | 0.005 | 0.0004883 | Island |  |  | Chr19:1725758 |  | I | TCF3 (-75473), ONECUT3 (-27903) |
| cg09123773 | 0.003 | -0.002 | 0.006 | 0.0004892 | Island | TSS1500 |  | Chr16:48419537 | SIAH1 | I | SIAH1 (-309) |
| cg21688288 | 0.027 | -0.026 | 0.053 | 0.0004912 |  | Body |  | Chr21:44181258 | PDE9A | II | PDE9A (+107397), WDR4 (+118419) |
| cg21294812 | -0.015 | 0.019 | -0.035 | 0.0004917 | Island | TSS200 |  | Chr6:132722770 | MOXD1 | I | MOXD1 (-107) |
| cg16048517 | 0.004 | -0.002 | 0.007 | 0.0004949 | Island | TSS1500 |  | Chr5:11904442 | CTNND2 | I | CTNND2 (-333) |
| cg18389933 | -0.022 | 0.034 | -0.057 | 0.0004961 | N_Shore |  |  | Chr14:57263993 |  | II | OTX2 (+13190), C14orf101 (+217483) |
| cg18414618 | 0.008 | -0.015 | -0.023 | 0.000505 | Island | TSS1500;TSS200 |  | Chr14:50999710 | MAP4K5;ATL1 | I | MAP4K5 (-335), ATL1 (-89) |
| cg11998425 | 0.013 | -0.017 | -0.030 | 0.0005069 | N_Shore | TSS1500 | rs3813771 | Chr19:8213583 | FBN3 | II | FBN3 (-1203) |
| cg02939090 | 0.004 | -0.005 | -0.009 | 0.0005169 | Island | 1stExon |  | Chr11:77348733 | CLNS1A | I | CLNS1A (+117) |
| cg14719352 | -0.040 | 0.004 | 0.045 | 0.0005182 | S_Shore | TSS1500;5'UTR |  | Chr19:49258131 | FGF21;FUT1 | II | FGF21 (-1016), FUT1 (+515) |
| cg02602601 | 0.005 | -0.019 | -0.024 | 0.0005215 | Island | TSS200 |  | Chr12:56618057 | OBFC2B | I | RNF41 (-2305), OBFC2B (-67) |
| cg21386099 | -0.041 | 0.027 | 0.068 | 0.0005281 |  | 3'UTR |  | Chr3:37476831 | C3orf35 | II | ITGA9 (-16981), C3orf35 (+35864) |
| cg15770125 | -0.014 | 0.042 | 0.056 | 0.0005289 |  | TSS200 |  | Chr3:8693781 | C3orf32 | II | CAV3 (-81704), LMCD1 (+150271) |
| cg15699623 | 0.035 | -0.012 | -0.046 | 0.0005365 | N_Shelf | Body |  | Chr19:42633695 | POU2F2 | II | POU2F2 (+2929), ZNF574 (+53406) |
| cg09236819 | 0.018 | -0.033 | -0.051 | 0.0005394 | Island | Body |  | Chr11:64876055 | C11orf2 | II | TM7SF2 (-3285) |
| cg07080946 | -0.008 | 0.044 | 0.051 | 0.0005399 | N_Shore | TSS1500 |  | Chr16:280046 | LUC7L | II | ITFG3 (-4754), LUC7L (-598) |
| cg00658394 | 0.005 | -0.020 | -0.025 | 0.0005402 | S_Shore | Body;TSS200 |  | Chr3:11685410 | VGLL4 | II | VGLL4 (-13) |
| cg23889013 | 0.029 | -0.024 | -0.053 | 0.0005408 | S_Shelf |  |  | Chr19:48710006 |  | II | C19orf68 (+36058), CARD8 (+43097) |
| cg01852049 | -0.023 | 0.035 | 0.058 | 0.0005408 | N_Shore | TSS1500 |  | Chr15:91072061 | CRTC3 | II | CRTC3 (-1136) |
| cg04928875 | 0.010 | -0.005 | -0.015 | 0.0005409 | Island | TSS200 |  | Chr2:128568924 | WDR33 | I | WDR33 (-164) |
| cg21199659 | -0.025 | 0.038 | 0.062 | 0.0005413 |  | Body |  | Chr9:133366752 | ASS1;ASS1 | II | FUBP3 (-88207), ASS1 (+46659) |
| cg07023791 | -0.012 | 0.009 | 0.021 | 0.0005428 | Island | TSS1500 |  | Chr10:83633980 | NRG3 | II | NRG3 (-1089) |
| cg05230854 | -0.061 | 0.014 | 0.075 | 0.0005447 |  |  |  | Chr8:8162989 |  | II | SGK223 (+76267), LOC100132396 (+379131) |
| cg14791747 | 0.035 | -0.006 | -0.041 | 0.0005464 | Island | 1stExon |  | Chr16:20752902 | THUMPD1 | II | ACSM1 (-50325), ACSM3 (-22409) |
| cg06614044 | -0.029 | 0.028 | 0.057 | 0.0005528 | Island | Body |  | Chr18:42324631 | SETBP1 | II | SLC14A2 (-468315), SETBP1 (+63769) |
| cg09648702 | 0.024 | -0.039 | -0.063 | 0.0005534 | Island |  |  | Chr11:20184779 |  | II | DBX1 (-2910) |
| cg06621900 | 0.016 | -0.016 | -0.031 | 0.0005553 |  | Body |  | Chr2:240239815 | HDAC4 | II | HDAC4 (+82827), TWIST2 (+483143) |
| cg14616584 | 0.031 | -0.008 | -0.040 | 0.0005639 |  | Body |  | Chr1:37388124 | GRIK3 | II | CSF3R (-439210), GRIK3 (+111719) |
| cg19862235 | -0.050 | 0.040 | 0.091 | 0.0005643 |  | 3'UTR |  | Chr20:7863971 | HAO1 | II | HAO1 (+57121) |
| cg15117681 | -0.021 | 0.039 | 0.060 | 0.0005645 | N_Shore | 3'UTR |  | Chr17:80202426 | CSNK1D | II | SLC16A3 (+16145), CSNK1D (+29167) |
| cg04387237 | 0.008 | -0.015 | -0.023 | 0.0005685 | Island | Body |  | Chr19:14640867 | TECR | II | TECR (+489) |
| cg21509821 | 0.015 | -0.012 | -0.028 | 0.0005696 | N_Shore | TSS1500 |  | Chr12:83079551 | TMTC2 | II | TMTC2 (-1382) |
| cg18016138 | -0.041 | 0.022 | 0.063 | 0.0005699 | N_Shore | TSS200 |  | Chr11:278421 | NLRP6 | I | NLRP6 (-148) |
| cg24028798 | 0.040 | -0.033 | -0.073 | 0.0005712 | S_Shelf | Body |  | Chr21:47322452 | PCBP3 | II | COL6A1 (-79210), PCBP3 (+52578) |
| cg16093537 | -0.032 | 0.030 | 0.062 | 0.0005738 |  | Body |  | Chr10:81924880 | ANXA11 | II | PLAC9 (+32623), ANXA11 (+40447) |
| cg08219700 | 0.064 | -0.047 | -0.111 | 0.0005755 | Island |  |  | Chr8:58056026 |  | II | FAM110B (-851086), IMPAD1 (-149597) |
| cg06781608 | 0.002 | -0.069 | -0.072 | 0.0005861 | N_Shore | Body |  | Chr7:157361040 | PTPRN2 | II | DNAJB6 (+231331) |
| cg07551054 | -0.003 | 0.003 | 0.007 | 0.0005924 | Island | Body |  | Chr8:140744200 | TRAPPC9 | I | KCNK9 (-28902), TRAPPC9 (+724477) |
| cg05886537 | 0.021 | -0.020 | -0.040 | 0.000598 | S_Shelf | Body |  | Chr11:132936428 | OPCML | II | OPCML (-123392), SPATA19 (+778963) |
| cg13678939 | 0.006 | -0.011 | -0.017 | 0.0005986 | Island | 1stExon |  | Chr11:62414063 | GANAB | I | GANAB (+40) |
| cg06549802 | -0.026 | 0.014 | 0.040 | 0.0006073 | N_Shore | 5'UTR;TSS1500 |  | Chr11:125756052 | HYLS1 | II | HYLS1 (+2544), PUS3 (+17063) |
| cg25725280 | 0.038 | -0.008 | -0.045 | 0.0006077 | Island | Body |  | Chr1:33625382 | TRIM62 | I | TRIM62 (+22288), ADC (+78669) |
| cg09529871 | -0.069 | 0.030 | 0.099 | 0.0006084 |  | TSS200 |  | Chr19:14911130 | OR7C1 | II | OR7C1 (-183) |
| cg22057372 | -0.015 | 0.040 | 0.055 | 0.0006099 |  | Body |  | Chr7:157711557 | PTPRN2 | II | DNAJB6 (+581848), PTPRN2 (+668924) |
| cg02056809 | -0.034 | 0.022 | 0.055 | 0.0006112 |  |  |  | Chr16:33605185 |  | II | BC068290 (-179325), TP53TG3B (+343066) |
| cg14224452 | -0.033 | 0.007 | 0.040 | 0.0006121 |  | 3'UTR |  | Chr10:103311489 | BTRC | II | POLL (+36537), BTRC (+197665) |
| cg10763059 | 0.017 | -0.024 | -0.041 | 0.0006131 | N_Shore | TSS1500 |  | Chr7:77165728 | PTPN12 | I | PTPN12 (-1044) |
| cg14098470 | -0.030 | 0.055 | 0.085 | 0.0006191 |  | Body |  | Chr6:29573253 | GABBR1 | II | OR2H2 (+17571), GABBR1 (+27708) |
| cg15382568 | 0.046 | -0.035 | -0.081 | 0.0006193 | N_Shore |  |  | Chr22:25800078 |  | II | ADRBK2 (-160782), LRP5L (-22535) |
| cg13728604 | -0.022 | 0.026 | 0.048 | 0.0006216 | N_Shelf | Body | rs72757203 | Chr9:132627095 | USP20 | II | USP20 (+29400), FNBP1 (+178377) |
| cg23687971 | 0.023 | -0.041 | -0.064 | 0.000629 | N_Shore | TSS1500 |  | Chr20:32262772 | NECAB3 | II | NECAB3 (-509) |
| cg11766468 | 0.036 | 0.113 | 0.077 | 0.0006307 | N_Shelf | Body |  | Chr19:7923041 | EVI5L | I | LRRC8E (-30348), EVI5L (+11660) |
| cg20406374 | 0.006 | -0.012 | -0.018 | 0.0006308 | Island | 5'UTR;Body |  | Chr5:179248352 | SQSTM1 | II | SQSTM1 (+511) |
| cg14114804 | 0.021 | -0.008 | -0.029 | 0.0006318 | Island | 5'UTR |  | Chr14:77606990 | ZDHHC22 | II | IRF2BPL (-111957), ZDHHC22 (+1143) |
| cg04090412 | 0.073 | -0.028 | -0.101 | 0.0006333 | Island | 3'UTR |  | Chr4:183721565 | ODZ3 | II | DCTD (+117064), ODZ3 (+476467) |
| cg20534694 | -0.039 | 0.032 | 0.071 | 0.0006334 |  | Body;TSS1500 |  | Chr7:151512686 | PRKAG2 | II | RHEB (-295677), PRKAG2 (+61629) |
| cg14674720 | 0.018 | -0.024 | -0.043 | 0.0006354 | Island |  |  | Chr2:219827930 |  | II | CDK5R2 (+3533), FEV (+22448) |
| cg09143221 | -0.044 | 0.017 | 0.061 | 0.0006365 | N_Shore | TSS1500;5'UTR |  | Chr17:3794657 | CAMKK1 | II | CAMKK1 (-621) |
| cg09670175 | 0.008 | -0.023 | -0.031 | 0.0006369 | S_Shelf | 3'UTR;1stExon |  | Chr12:4923468 | KCNA6 | II | KCNA1 (-95604), KCNA6 (+5127) |
| cg03323397 | -0.034 | 0.013 | 0.047 | 0.0006399 | N_Shelf | Body |  | Chr6:30519312 | GNL1 | II | PRR3 (-5173), HLA-E (+62130) |
| cg05193407 | -0.025 | 0.025 | 0.050 | 0.0006399 |  | TSS200 |  | Chr4:57036298 | KIAA1211 | II | AASDH (+217339), CEP135 (+221262) |
| cg01988480 | 0.038 | -0.025 | -0.063 | 0.0006522 | Island | TSS1500;5'UTR |  | Chr11:93474146 | C11orf54;TAF1D | I | C11orf54 (-646), TAF1D (+556) |
| cg08711281 | -0.039 | 0.023 | 0.062 | 0.0006554 |  | TSS1500 |  | Chr6:30908302 | DPCR1 | II | DPCR1 (-474) |
| cg16016036 | -0.028 | 0.019 | 0.046 | 0.0006572 |  | TSS200;TSS1500 |  | Chr2:1417109 | TPO | II | TPO (-123) |
| cg13650687 | 0.016 | -0.026 | -0.042 | 0.0006583 |  | Body |  | Chr10:71655974 | COL13A11 | II | H2AFY2 (-156382), COL13A1 (+94331) |
| cg01832757 | 0.031 | -0.041 | -0.072 | 0.00066 | N_Shelf |  |  | Chr6:159547894 |  | II | TAGAP (-81711), FNDC1 (-42534) |
| cg19791379 | -0.017 | 0.009 | 0.026 | 0.0006602 |  | Body |  | Chr12:1555160 | ERC1 | II | FBXL14 (+148170), ERC1 (+454757) |
| cg04015907 | -0.009 | 0.017 | 0.025 | 0.000664 | Island | Body;1stExon |  | Chr20:825489 | FAM110A | I | FAM110A (+11134), ANGPT4 (+71470) |
| cg21637015 | -0.008 | 0.005 | 0.013 | 0.000667 |  | Body |  | Chr3:129275285 | PLXND1 | I | H1FOO (+13229), PLXND1 (+50296) |
| cg06401614 | -0.048 | 0.019 | 0.066 | 0.00067 |  | Body;1stExon |  | Chr11:44927976 | TSPAN18;TSPAN18 | II | TP53I11 (+44631), TSPAN18 (+142001) |
| cg26335251 | -0.032 | 0.035 | 0.067 | 0.0006707 |  |  |  | Chr17:75539921 |  | I | TNRC6C (-460396), SEPT9 (+262430) |
| cg21915639 | 0.021 | -0.010 | -0.031 | 0.0006715 | S_Shore | TSS1500;TSS200 |  | Chr1:46769308 | LRRC41;UQCRH | I | UQCRH (-71) |
| cg25104124 | 0.013 | -0.018 | -0.031 | 0.0006719 | Island |  |  | Chr10:74021022 |  | II | ASCC1 (-45156), DDIT4 (-12654) |
| cg03152785 | -0.014 | 0.010 | 0.024 | 0.0006747 | Island | TSS1500 |  | Chr1:110753723 | KCNC4 | I | KCNC4 (+388) |
| cg14987769 | 0.035 | -0.009 | -0.044 | 0.0006763 | S_Shore | Body |  | Chr2:220197576 | RESP18 | II | RESP18 (+322) |
| cg19107120 | -0.033 | 0.031 | 0.064 | 0.0006765 | N_Shore | 3'UTR |  | Chr17:42430376 | GRN | II | GRN (+7886), FAM171A2 (+10858) |
| cg03368399 | 0.000 | -0.020 | -0.020 | 0.0006803 | Island | TSS200 |  | Chr8:22102556 | POLR3D;MIR320A | I | POLR3D (-62) |
| cg07608033 | -0.023 | 0.021 | 0.044 | 0.0006834 | S_Shelf |  |  | Chr10:36056930 |  | II | FZD8 (-126569) |
| cg27135125 | 0.024 | -0.013 | -0.037 | 0.0006834 | S_Shore | TSS200 |  | Chr7:23510082 | IGF2BP3 | I | IGF2BP3 (-88) |
| cg13484614 | -0.031 | 0.031 | 0.062 | 0.0006848 | S_Shore | TSS1500 |  | Chr9:127178389 | PSMB7 | II | PSMB7 (-669) |
| cg07841529 | 0.024 | -0.060 | -0.084 | 0.0006893 | N_Shore |  |  | Chr17:47269182 |  | II | ABI3 (-18406), B4GALNT2 (+58854) |
| cg07717903 | 0.010 | 0.082 | 0.072 | 0.0006922 | N_Shore | Body;TSS200 |  | Chr5:140753536 | PCDHGA4 | II | PCDHGC5 (-115271), TAF7 (-53186) |
| cg00045190 | 0.005 | -0.003 | -0.007 | 0.0006928 | Island |  |  | Chr6:33216612 |  | I | VPS52 (+23049), RING1 (+40327) |
| cg00083262 | 0.013 | -0.040 | -0.054 | 0.0006952 |  |  |  | Chr1:157125526 |  | II | ETV3 (-17144), FCRL5 (+396783) |
| cg08495020 | -0.031 | 0.024 | 0.055 | 0.0007023 |  | TSS200 |  | Chr19:17571888 | NXNL1 | II | NXNL1 (-164) |
| cg09859456 | 0.008 | -0.014 | -0.022 | 0.0007072 | S_Shore | 5'UTR;Body |  | Chr12:72234313 | TBC1D15 | I | TBC1D15 (+827) |
| cg22837512 | -0.005 | 0.011 | 0.016 | 0.0007093 | Island | TSS200 |  | Chr11:108093386 | ATM;NPAT | I | ATM (-172), NPAT (-22) |
| cg05730365 | 0.004 | -0.038 | -0.042 | 0.0007154 | Island | 5'UTR |  | Chr5:37838563 | GDNF | II | GDNF (-2635) |
| cg09719850 | -0.074 | 0.043 | 0.116 | 0.0007157 |  |  |  | Chr12:6993936 |  | II | LRRC23 (-19960), SPSB2 (-11488) |
| cg10859966 | 0.009 | -0.011 | -0.020 | 0.0007159 | Island | TSS1500;TSS200 |  | Chr14:24898992 | CBLN3;KHNYN | I | CBLN3 (-262), KHNYN (-148) |
| cg25630594 | -0.043 | 0.032 | 0.075 | 0.000718 |  | TSS1500 |  | Chr3:44914870 | TGM4 | I | TGM4 (-1227) |
| cg09730361 | -0.008 | 0.005 | 0.013 | 0.0007183 | S_Shore | TSS200 |  | Chr9:20622599 | MLLT3 | II | MLLT3 (-86) |
| cg00123072 | -0.045 | 0.017 | 0.062 | 0.0007183 | N_Shore | TSS1500 |  | Chr5:79330259 | THBS4 | II | THBS4 (-910) |
| cg10177795 | 0.041 | -0.014 | -0.055 | 0.0007221 |  | Body |  | Chr2:242331545 | FARP2 | II | FARP2 (+35882), STK25 (+116488) |
| cg06402590 | -0.023 | 0.037 | 0.061 | 0.0007272 | S_Shelf | Body |  | Chr16:2820155 | SRRM2 | II | TCEB2 (+7141), SRRM2 (+17826) |
| cg26457569 | -0.028 | 0.057 | 0.085 | 0.0007282 | Island | Body |  | Chr19:11280837 | KANK2 | II | C19orf80 (-69457), SPC24 (-14354) |
| cg00940891 | -0.005 | 0.004 | 0.008 | 0.0007374 | Island | 5'UTR;TSS1500 |  | Chr4:1006016 | FGFRL1 | I | FGFRL1 (+257) |
| cg00175987 | -0.030 | 0.027 | 0.057 | 0.0007377 |  | TSS1500 |  | Chr18:72342044 | ZNF407 | II | ZNF407 (-878) |
| cg19717216 | -0.034 | 0.053 | 0.086 | 0.0007414 | N_Shore | Body |  | Chr19:4171754 | CREB3L3 | I | SIRT6 (+10841), CREB3L3 (+18126) |
| cg06179039 | -0.017 | 0.026 | 0.043 | 0.0007414 | S_Shelf | 3'UTR |  | Chr16:67263524 | FHOD1 | II | TMEM208 (+2509), FHOD1 (+17900) |
| cg19522185 | -0.045 | 0.029 | 0.073 | 0.0007426 | N_Shelf |  |  | Chr10:81103810 |  | II | PPIF (-3409) |
| cg04291430 | 0.028 | -0.016 | -0.044 | 0.0007437 | N_Shore | 5'UTR |  | Chr17:48206283 | SAMD14 | II | PPP1R9B (+21593), PDK2 (+33645) |
| cg12927270 | 0.007 | -0.008 | -0.015 | 0.0007447 | Island | TSS200 |  | Chr3:94656950 | LOC255025 | I | DHFRL1 (-874884) |
| cg12658052 | -0.062 | 0.005 | 0.067 | 0.0007473 |  |  |  | Chr1:1078295 |  | II | TTLL10 (-30990), C1orf159 (-26560) |
| cg05045702 | -0.027 | 0.026 | 0.054 | 0.0007488 | Island | Body |  | Chr16:30616285 | ZNF689 | II | ZNF785 (-19194), ZNF689 (+5396) |
| cg05925971 | 0.004 | -0.014 | -0.019 | 0.0007514 | Island | TSS200 |  | Chr19:36485966 | SDHAF1 | I | SDHAF1 (-123) |
| cg07817400 | 0.012 | -0.009 | -0.022 | 0.000753 | Island | Body |  | Chr6:33172817 | HSD17B8 | II | RXRB (-4386), RING1 (-3468) |
| cg05173373 | 0.003 | -0.021 | -0.023 | 0.0007533 | S_Shore | TSS1500 |  | Chr12:15942933 | EPS8 | II | EPS8 (-424) |
| cg00642468 | -0.005 | 0.004 | 0.009 | 0.000761 | Island | TSS1500;TSS200 |  | Chr13:111364924 | ING1 | I | ING1 (-2434) |
| cg27659478 | -0.016 | 0.004 | 0.020 | 0.0007665 | N_Shore | Body |  | Chr17:73891062 | TRIM65 | I | TRIM47 (-16407), TRIM65 (+1991) |
| cg05769790 | 0.008 | -0.005 | -0.013 | 0.0007686 | Island | TSS200 |  | Chr6:126661238 | C6orf173 | II | CENPW (-14) |
| cg02567788 | 0.010 | -0.013 | -0.023 | 0.0007692 | N_Shore | Body |  | Chr10:71992279 | PPA1 | II | PPA1 (+910) |
| cg21613693 | -0.047 | 0.022 | 0.069 | 0.0007725 | Island | Body |  | Chr17:73501181 | CASKIN2 | II | CASKIN2 (+10445), KIAA0195 (+48518) |
| cg04212651 | -0.034 | 0.023 | 0.057 | 0.0007738 |  | Body |  | Chr6:112671523 | RFPL4B | II | RFPL4B (+2992) |
| cg22249386 | -0.038 | 0.018 | 0.056 | 0.000779 |  | Body |  | Chr17:10212450 | MYH13 | II | GAS7 (-110583), MYH13 (+63871) |
| cg27147114 | -0.013 | 0.028 | 0.041 | 0.0007817 |  | Body |  | Chr15:79358128 | RASGRF1 | II | CTSH (-120709), RASGRF1 (+25086) |
| cg07150629 | -0.007 | 0.008 | 0.015 | 0.0007829 | S_Shore | 5'UTR;TSS1500 |  | Chr4:40059484 | N4BP2;LOC344967 | II | LOC344967 (-666), N4BP2 (+961) |
| cg00817367 | 0.009 | -0.020 | -0.030 | 0.0007861 | Island | Body |  | Chr12:52401214 | GRASP | I | GRASP (+467) |
| cg01017689 | 0.040 | -0.018 | -0.058 | 0.0007871 | S_Shelf | Body;5'UTR |  | Chr5:76376266 | SNORA47;ZBED3 | II | ZBED3 (+6763), AGGF1 (+50057) |
| cg21368481 | -0.027 | 0.048 | 0.076 | 0.0007896 |  | Body |  | Chr6:31747210 | VARS | II | VWA7 (-2103) |
| cg04330884 | 0.039 | -0.008 | -0.047 | 0.0007916 |  | Body |  | Chr10:126339578 | FAM53B | I | FAM53B (+93351), LHPP (+189238) |
| cg25121621 | 0.017 | -0.044 | -0.060 | 0.0007918 | N_Shore | TSS1500 |  | Chr15:45926780 | SQRDL | II | SQRDL (-475) |
| cg14496375 | -0.025 | 0.027 | 0.053 | 0.0007971 | S_Shore | TSS200 |  | Chr19:51872365 | CLDND2 | II | CLDND2 (-109) |
| cg04106390 | -0.018 | 0.011 | 0.028 | 0.0007997 | N_Shelf | Body |  | Chr16:1224897 | CACNA1H | I | CACNA1H (+21657), TPSG1 (+50356) |
| cg04535902 | -0.065 | 0.095 | 0.160 | 0.0008055 | Island | Body |  | Chr1:92947332 | GFI1 | II | GLMN (-182767), GFI1 (+4295) |
| cg10805039 | 0.024 | -0.055 | -0.079 | 0.0008071 |  | Body |  | Chr17:750241 | NXN | II | GLOD4 (-64671), NXN (+132756) |
| cg05550276 | 0.011 | 0.000 | -0.011 | 0.0008104 | Island | 5'UTR;Body |  | Chr16:52580524 | TOX3 | I | TOX3 (+281) |
| cg26060667 | 0.027 | -0.077 | -0.103 | 0.0008105 | N_Shore |  |  | Chr1:247681242 |  | II | OR2C3 (+15898), OR2W5 (+26873) |
| cg27081107 | -0.014 | 0.024 | 0.038 | 0.0008123 |  | Body |  | Chr10:438661 | DIP2C | I | ZMYND11 (+212728), DIP2C (+296946) |
| cg19192280 | 0.031 | -0.074 | -0.105 | 0.0008126 | Island | 3'UTR |  | Chr6:32116893 | PRRT1 | I | PPT2 (-4335), PRRT1 (+2826) |
| cg00387551 | -0.061 | 0.037 | 0.098 | 0.0008128 |  | Body |  | Chr1:3011304 | PRDM16 | II | ARHGEF16 (-359842), PRDM16 (+25563) |
| cg04554564 | -0.053 | 0.037 | 0.090 | 0.0008173 |  | TSS200 |  | Chr17:19990302 | CYTSB | II | SPECC1 (+77654), LGALS9B (+380545) |
| cg12643226 | 0.027 | -0.015 | -0.041 | 0.0008174 |  | Body |  | Chr1:115829262 | NGF | I | TSPAN2 (-197142), NGF (+51594) |
| cg27376437 | 0.005 | -0.018 | -0.023 | 0.0008187 | N_Shore | Body |  | Chr17:56064565 | VEZF1 | II | MRPS23 (-137133), VEZF1 (+1049) |
| cg13676706 | 0.010 | -0.018 | -0.028 | 0.0008189 | S_Shelf |  |  | Chr12:127944448 |  | II | TMEM132C (-807499) |
| cg05982929 | 0.003 | -0.018 | -0.021 | 0.0008203 | Island | TSS1500;Body |  | Chr6:32163523 | GPSM3;NOTCH4 | I | GPSM3 (-224) |
| cg21173803 | 0.042 | -0.013 | -0.054 | 0.0008215 |  |  |  | Chr22:27299879 |  | II | CRYBA4 (+281952), MN1 (+897606) |
| cg10202994 | 0.042 | -0.021 | -0.064 | 0.0008226 | N_Shore | TSS1500 |  | Chr2:220071050 | ZFAND2B | II | ZFAND2B (-487) |
| cg00739667 | -0.028 | 0.036 | 0.064 | 0.0008254 |  | Body |  | Chr16:81731648 | CMIP | I | PLCG2 (-81250), CMIP (+252874) |
| cg22823236 | -0.045 | 0.057 | 0.101 | 0.0008272 | N_Shelf | 5'UTR |  | Chr14:70652710 | SLC8A3 | II | SLC8A3 (+3076), SMOC1 (+306597) |
| cg17330838 | -0.056 | 0.031 | 0.087 | 0.0008353 | Island | Body |  | Chr11:57267101 | SLC43A1 | I | SLC43A1 (+15256), RTN4RL2 (+38763) |
| cg01249202 | -0.021 | 0.027 | 0.047 | 0.0008361 |  |  |  | Chr13:78410079 |  | II | EDNRB (+83823), SCEL (+300271) |
| cg19879479 | 0.007 | -0.019 | -0.027 | 0.0008447 | N_Shore | 5'UTR;5'UTR |  | Chr6:80246572 | LCA5 | I | LCA5 (+574) |
| cg23013958 | 0.043 | -0.082 | -0.125 | 0.0008456 | N_Shore | TSS1500 |  | Chr1:65612865 | AK3L1 | II | AK4 (-366) |
| cg02508664 | 0.040 | -0.022 | -0.061 | 0.0008465 | N_Shore | TSS1500 | rs828363 | Chr6:87646738 | HTR1E | II | HTR1E (-285) |
| cg14452650 | 0.007 | -0.007 | -0.013 | 0.0008499 | N_Shore | TSS1500 |  | Chr2:42396274 | EML4 | I | EML4 (-215) |
| cg25730717 | 0.015 | -0.020 | -0.035 | 0.0008512 | Island | TSS1500 |  | Chr7:148726076 | PDIA4 | I | PDIA4 (-295) |
| cg08313539 | 0.084 | 0.005 | -0.079 | 0.0008512 |  |  |  | Chr6:127094710 |  | II | RSPO3 (-345337), CENPW (+433458) |
| cg09847284 | 0.005 | -0.025 | -0.030 | 0.000858 | Island | TSS200 |  | Chr11:45907071 | MAPK8IP1 | I | MAPK8IP1 (+25) |
| cg01112527 | -0.027 | 0.045 | 0.072 | 0.0008585 | N_Shore | Body |  | Chr2:1479810 | TPO | II | TPO (+62578), PXDN (+268480) |
| cg06466917 | -0.008 | 0.015 | 0.023 | 0.0008585 | N_Shore | Body;TSS1500 |  | Chr5:17216259 | LOC285696;BASP1 | II | BASP1 (-1490) |
| cg14964512 | 0.042 | -0.033 | -0.075 | 0.0008591 | Island |  |  | Chr19:53832493 |  | I | ZNF845 (-4508) |
| cg13842154 | 0.039 | -0.023 | -0.062 | 0.00086 | Island | Body |  | Chr14:103390247 | AMN | II | AMN (+1255), CDC42BPB (+133494) |
| cg08027943 | -0.047 | 0.020 | 0.067 | 0.0008607 |  |  |  | Chr14:53833982 |  | II | DDHD1 (-213937), BMP4 (+587287) |
| cg12650685 | -0.019 | 0.009 | 0.028 | 0.000863 |  | Body |  | Chr6:3284109 | SLC22A23 | II | TUBB2B (-56142), SLC22A23 (+172683) |
| cg03004426 | 0.007 | -0.011 | -0.019 | 0.0008632 | Island | TSS200 |  | Chr16:53537293 | AKTIP | II | AKTIP (-124) |
| cg18923635 | -0.020 | 0.038 | 0.058 | 0.0008665 | N_Shore |  |  | Chr18:48083994 |  | II | MAPK4 (-2489) |
| cg24377285 | -0.038 | 0.027 | 0.065 | 0.0008667 | Island | Body |  | Chr19:38943724 | RYR1;RYR1 | I | RYR1 (+19385), MAP4K1 (+164918) |
| cg15589930 | 0.015 | -0.011 | -0.026 | 0.0008679 | S_Shore | TSS1500 |  | Chr1:94344944 | DNTTIP2 | II | DNTTIP2 (-183) |
| cg08169950 | -0.028 | 0.072 | 0.101 | 0.0008722 | N_Shelf |  |  | Chr1:247799571 |  | II | OR2G3 (+30684), OR13G1 (+36771) |
| cg24789562 | 0.024 | -0.019 | -0.044 | 0.0008736 |  |  |  | Chr11:27842648 |  | II | BDNF (-120049), KIF18A (+287097) |
| cg01412524 | 0.003 | -0.008 | -0.011 | 0.0008749 | Island | Body | rs72890727 | Chr1:46860243 | FAAH | I | FAAH (+305) |
| cg17474545 | -0.019 | 0.046 | 0.066 | 0.0008775 | S_Shore |  |  | Chr19:56061873 |  | II | SGK110 (-4965) |
| cg04043957 | -0.018 | 0.051 | 0.070 | 0.000879 |  | TSS1500;TSS200 |  | Chr11:1860215 | TNNI2 | I | TNNI2 (-1216) |
| cg24842354 | -0.056 | 0.058 | 0.114 | 0.0008831 | N_Shore | TSS1500;Body |  | Chr1:2004057 | PRKCZ | II | SKI (-156076), PRKCZ (+22149) |
| cg05938623 | 0.019 | 0.000 | -0.019 | 0.000884 | Island |  |  | Chr1:223254899 |  | II | TLR5 (+61724), DISP1 (+266469) |
| cg20053381 | 0.002 | -0.014 | -0.017 | 0.0008862 | S_Shore | TSS200;TSS200 |  | Chr2:220363572 | GMPPA | I | GMPPA (-14) |
| cg13580027 | -0.034 | 0.057 | 0.091 | 0.0008876 | N_Shore |  |  | Chr10:134972063 |  | II | KNDC1 (-1907) |
| cg14711428 | 0.015 | -0.003 | -0.018 | 0.000888 | Island | TSS200;5'UTR |  | Chr7:112090348 | IFRD1 | I | C7orf53 (-30559), IFRD1 (+27150) |
| cg23399257 | 0.022 | 0.068 | 0.046 | 0.0008911 |  |  |  | Chr2:394481 |  | II | FAM150B (-106174), TMEM18 (+282957) |
| cg11832544 | 0.015 | -0.021 | -0.036 | 0.0008914 |  | Body |  | Chr1:161094035 | DEDD | II | PFDN2 (-6170), DEDD (+8220) |
| cg21937169 | 0.006 | -0.025 | -0.031 | 0.0008938 | S_Shore | Body |  | Chr7:65541137 | ASL | II | ASL (+362) |
| cg17623882 | -0.019 | 0.009 | 0.028 | 0.0008952 | N_Shore | Body |  | Chr6:41773611 | USP49 | I | TOMM6 (+19112), USP49 (+89487) |
| cg12293132 | 0.018 | -0.012 | -0.031 | 0.0008952 | Island | Body |  | Chr7:23720668 | C7orf46 | II | STK31 (-29169), CCDC126 (+83671) |
| cg05658771 | -0.040 | 0.032 | 0.072 | 0.0008988 | S_Shore | TSS1500 |  | Chr16:89884179 | FANCA | I | FANCA (-1115) |
| cg16442298 | 0.028 | -0.043 | -0.071 | 0.0009015 | N_Shore |  |  | Chr8:1403050 |  | II | C8orf42 (-907720), DLGAP2 (-46518) |
| cg15975990 | 0.046 | -0.038 | -0.084 | 0.0009016 | Island | TSS200 |  | Chr2:180871849 | CWC22 | I | CWC22 (-70) |
| cg13890706 | 0.005 | -0.018 | -0.023 | 0.0009027 | Island | 1stExon;5'UTR;TSS1500 |  | Chr10:118033115 | GFRA1 | II | GFRA1 (+10) |
| cg10281478 | -0.030 | 0.028 | 0.058 | 0.0009069 | S_Shore | 5'UTR |  | Chr7:95402528 | DYNC1I1 | II | DYNC1I1 (+711) |
| cg01948724 | -0.012 | 0.058 | 0.070 | 0.0009073 |  |  |  | Chr16:3038035 |  | II | CLDN9 (-24421), PKMYT1 (-7496) |
| cg17024952 | 0.002 | -0.033 | -0.035 | 0.0009092 | S_Shore | Body |  | Chr6:116422486 | NT5DC1 | II | NT5DC1 (+488) |
| cg12231340 | -0.026 | 0.052 | 0.078 | 0.0009107 | Island | 1stExon |  | Chr12:52685221 | KRT81 | II | KRT81 (+77) |
| cg07107628 | -0.026 | 0.025 | 0.051 | 0.0009113 |  |  |  | Chr6:27210934 |  | II | PRSS16 (-4567) |
| cg07654559 | 0.021 | -0.017 | -0.039 | 0.0009125 | S_Shore | Body;TSS1500 |  | Chr2:74710618 | TTC31;CCDC142;TTC31 | I | CCDC142 (-262), TTC31 (+419) |
| cg24674304 | 0.035 | -0.026 | -0.061 | 0.0009177 | Island | TSS1500 |  | Chr3:44903063 | MIR564;TMEM42 | I | TMEM42 (-344) |
| cg22076495 | 0.009 | -0.009 | -0.019 | 0.0009237 | Island | TSS1500 |  | Chr5:32174716 | GOLPH3 | II | GOLPH3 (-292) |
| cg02101876 | 0.023 | -0.026 | -0.049 | 0.0009245 |  |  |  | Chr13:40765110 |  | II | FOXO1 (+475623), COG6 (+535347) |
| cg09186897 | 0.010 | -0.006 | -0.016 | 0.0009294 | Island | 5'UTR |  | Chr1:169862509 | SCYL3 | I | SCYL3 (+566) |
| cg25936902 | 0.013 | -0.063 | -0.076 | 0.0009307 | S_Shore |  |  | Chr13:30982971 |  | II | KATNAL1 (-101809), HMGB1 (+57109) |
| cg08532057 | -0.041 | 0.029 | 0.070 | 0.0009356 | Island | TSS1500 |  | Chr13:25875436 | NUPL1 | II | NUPL1 (-229) |
| cg25064029 | -0.036 | 0.033 | 0.069 | 0.0009358 |  | 3'UTR |  | Chr4:113362371 | ALPK1 | II | NEUROG2 (+74956), ALPK1 (+143873) |
| cg09231514 | 0.006 | -0.005 | -0.011 | 0.0009436 | Island | 5'UTR;TSS1500 |  | Chr19:5681323 | HSD11B1L;C19orf70 | I | C19orf70 (-413), HSD11B1L (+289) |
| cg14543966 | 0.014 | -0.036 | -0.051 | 0.0009472 |  | Body |  | Chr9:123537218 | FBXW2 | II | MEGF9 (-60454), FBXW2 (+18521) |
| cg20668450 | -0.045 | 0.026 | 0.070 | 0.0009483 | N_Shelf | 3'UTR |  | Chr11:64812208 | SAC3D1 | II | SAC3D1 (+3833), NAALADL1 (+13800) |
| cg21594651 | -0.027 | 0.034 | 0.061 | 0.0009501 |  | Body |  | Chr4:79214428 | FRAS1 | II | ANXA3 (-258313), FRAS1 (+235705) |
| cg26250400 | 0.030 | -0.066 | -0.096 | 0.0009528 | S_Shelf |  |  | Chr19:15921959 |  | II | OR10H1 (-3024) |
| cg04998447 | 0.038 | -0.022 | -0.060 | 0.0009559 | Island | Body |  | Chr15:90209223 | PLIN1 | II | KIF7 (-10542), PLIN1 (+13424) |
| cg12667002 | -0.025 | 0.050 | 0.075 | 0.000958 | N_Shore | Body |  | Chr13:114812302 | RASA3 | II | GAS6 (-245257), RASA3 (+85792) |
| cg02401132 | 0.007 | -0.016 | -0.022 | 0.0009594 | S_Shore |  |  | Chr6:19805404 |  | II | ID4 (-32212) |
| cg25058261 | 0.063 | -0.082 | -0.144 | 0.0009618 | N_Shore | 3'UTR |  | Chr12:115109452 | TBX3 | II | TBX5 (-265564), TBX3 (+12516) |
| cg16909783 | -0.007 | 0.016 | 0.022 | 0.0009659 | Island |  |  | Chr7:150869917 |  | I | GBX1 (-5051), ASB10 (+15001) |
| cg08580032 | -0.026 | 0.026 | 0.051 | 0.000969 | Island |  |  | Chr18:77562500 |  | II | KCNG2 (-61167), CTDP1 (+122700) |
| cg17614974 | -0.016 | 0.008 | 0.024 | 0.0009716 | Island |  |  | Chr3:44039919 |  | II | C3orf23 (-340024), ABHD5 (+307545) |
| cg06719334 | 0.017 | -0.023 | -0.040 | 0.0009729 |  | Body |  | Chr16:7501308 | A2BP1 | II | RBFOX1 (+118558) |
| cg14478589 | 0.035 | -0.043 | -0.078 | 0.0009799 |  | Body |  | Chr9:124086862 | GSN | II | GSN (+24784), STOM (+45682) |
| cg23334298 | -0.041 | 0.024 | 0.064 | 0.0009807 |  | Body |  | Chr4:78692537 | CNOT6L | II | CNOT6L (+48006), CXCL13 (+259631) |
| cg06142142 | -0.038 | 0.025 | 0.062 | 0.0009809 | N_Shore | TSS1500 |  | Chr10:115938512 | TDRD1 | II | TDRD1 (-516) |
| cg19526568 | 0.036 | -0.009 | -0.045 | 0.0009835 | Island |  |  | Chr13:100643087 |  | II | PCCA (-98181), ZIC2 (+9062) |
| cg25004270 | -0.005 | 0.018 | 0.024 | 0.0009876 | Island | TSS1500;1stExon |  | Chr1:1550864 | MIB2 | I | MIB2 (+70) |
| cg26665082 | 0.007 | -0.011 | -0.018 | 0.0009883 | Island | TSS200 |  | Chr1:78444860 | FUBP1 | I | FUBP1 (-84) |
| cg14060757 | -0.043 | 0.007 | 0.050 | 0.0009887 | Island | Body |  | Chr21:46715144 | LOC642852 | I | COL18A1 (-160279), POFUT2 (-7334) |
| cg02898665 | -0.017 | 0.065 | 0.082 | 0.0009982 | N_Shore |  |  | Chr8:55368762 |  | II | SOX17 (-1732) |
| cg08886036 | -0.014 | 0.008 | 0.022 | 0.0009986 | S_Shelf | Body |  | Chr1:1118934 | TTLL10 | I | TTLL10 (+9649), TNFRSF18 (+23154) |

*Note.* Ranked by *p* value. ∆β, difference in DNA methylation; DMPs, differentially methylated positions; GREAT, Genomic Regions Enrichment of Annotations Tool; Hg19, Human Genome build 19; SBE, single-base extension; SNP, single nucleotide polymorphism; TSS, transcription start site.

**Supplementary Table 7.** Gene Ontology (GO) enrichment analysis of longitudinal DMPs between age 5 and age 10

| **GO Accession ID** | **GO Function** | **Ontology** | **P value** |
| --- | --- | --- | --- |
| GO:0042734 | presynaptic membrane | CC | 0.00317 |
| GO:0043204 | perikaryon | CC | 0.00347 |
| GO:0060993 | kidney morphogenesis | BP | 0.00597 |
| GO:0005783 | endoplasmic reticulum | CC | 0.00623 |
| GO:0031941 | filamentous actin | CC | 0.00658 |
| GO:0016525 | negative regulation of angiogenesis | BP | 0.00666 |
| GO:0072075 | metanephric mesenchyme development | BP | 0.00732 |
| GO:0003338 | metanephros morphogenesis | BP | 0.00897 |
| GO:0072074 | kidney mesenchyme development | BP | 0.01068 |
| GO:0042157 | lipoprotein metabolic process | BP | 0.01089 |
| GO:0000050 | urea cycle | BP | 0.01104 |
| GO:0060231 | mesenchymal to epithelial transition | BP | 0.01224 |
| GO:0019627 | urea metabolic process | BP | 0.01226 |
| GO:0045921 | positive regulation of exocytosis | BP | 0.01306 |
| GO:0010870 | positive regulation of receptor biosynthetic process | BP | 0.01336 |
| GO:0071941 | nitrogen cycle metabolic process | BP | 0.01397 |
| GO:0051219 | phosphoprotein binding | MF | 0.01424 |
| GO:0032799 | low-density lipoprotein receptor particle metabolic process | BP | 0.01456 |
| GO:0035020 | regulation of Rac protein signal transduction | BP | 0.01675 |
| GO:0010984 | regulation of lipoprotein particle clearance | BP | 0.01706 |
| GO:0072215 | regulation of metanephros development | BP | 0.01725 |
| GO:0032839 | dendrite cytoplasm | CC | 0.01985 |
| GO:0006004 | fucose metabolic process | BP | 0.02103 |
| GO:0006497 | protein lipidation | BP | 0.02146 |
| GO:0009084 | glutamine family amino acid biosynthetic process | BP | 0.0218 |
| GO:0071385 | cellular response to glucocorticoid stimulus | BP | 0.02222 |
| GO:0016226 | iron-sulfur cluster assembly | BP | 0.02232 |
| GO:0031163 | metallo-sulfur cluster assembly | BP | 0.02232 |
| GO:0071384 | cellular response to corticosteroid stimulus | BP | 0.02246 |
| GO:0051016 | barbed-end actin filament capping | BP | 0.02251 |
| GO:0016601 | Rac protein signal transduction | BP | 0.026 |
| GO:0042158 | lipoprotein biosynthetic process | BP | 0.02623 |
| GO:0043604 | amide biosynthetic process | BP | 0.02646 |
| GO:0065003 | macromolecular complex assembly | BP | 0.02702 |
| GO:0031984 | organelle subcompartment | CC | 0.03066 |
| GO:0045879 | negative regulation of smoothened signaling pathway | BP | 0.03228 |
| GO:0003337 | mesenchymal to epithelial transition involved in metanephros morphogenesis | BP | 0.03284 |
| GO:0006461 | protein complex assembly | BP | 0.0332 |
| GO:0008083 | growth factor activity | MF | 0.03345 |
| GO:0070271 | protein complex biogenesis | BP | 0.03374 |
| GO:0034383 | low-density lipoprotein particle clearance | BP | 0.03382 |
| GO:0010869 | regulation of receptor biosynthetic process | BP | 0.03463 |
| GO:0009163 | nucleoside biosynthetic process | BP | 0.03466 |
| GO:1901659 | glycosyl compound biosynthetic process | BP | 0.03466 |
| GO:0036092 | phosphatidylinositol-3-phosphate biosynthetic process | BP | 0.03638 |
| GO:0015758 | glucose transport | BP | 0.0365 |
| GO:0061299 | retina vasculature morphogenesis in camera-type eye | BP | 0.03672 |
| GO:0008645 | hexose transport | BP | 0.03712 |
| GO:0051259 | protein oligomerization | BP | 0.03724 |
| GO:0015749 | monosaccharide transport | BP | 0.03867 |
| GO:0006525 | arginine metabolic process | BP | 0.0393 |
| GO:0005758 | mitochondrial intermembrane space | CC | 0.03952 |
| GO:0003382 | epithelial cell morphogenesis | BP | 0.04079 |
| GO:0030857 | negative regulation of epithelial cell differentiation | BP | 0.04085 |
| GO:0032314 | regulation of Rac GTPase activity | BP | 0.04242 |
| GO:0071634 | regulation of transforming growth factor beta production | BP | 0.04323 |
| GO:0007269 | neurotransmitter secretion | BP | 0.04344 |
| GO:0004675 | transmembrane receptor protein serine/threonine kinase activity | MF | 0.04352 |
| GO:0005024 | transforming growth factor beta-activated receptor activity | MF | 0.04352 |
| GO:1902931 | negative regulation of alcohol biosynthetic process | BP | 0.0443 |
| GO:0072088 | nephron epithelium morphogenesis | BP | 0.04443 |
| GO:0010827 | regulation of glucose transport | BP | 0.04607 |
| GO:0019433 | triglyceride catabolic process | BP | 0.04607 |
| GO:0010894 | negative regulation of steroid biosynthetic process | BP | 0.04676 |
| GO:0000578 | embryonic axis specification | BP | 0.04714 |
| GO:0001895 | retina homeostasis | BP | 0.04838 |
| GO:0032800 | receptor biosynthetic process | BP | 0.04924 |

*Note.* BP, Biological Processes; CC, Cellular; DMPs, differentially methylated positions; MF, Molecular Function.
